# The NADH recycling enzymes TsaC and TsaD regenerate reducing equivalents for Rieske oxygenase chemistry

**DOI:** 10.1016/j.jbc.2023.105222

**Published:** 2023-09-09

**Authors:** Jiayi Tian, David G. Boggs, Patrick H. Donnan, Gage T. Barroso, Alejandro Arcadio Garcia, Daniel P. Dowling, Joshua A. Buss, Jennifer Bridwell-Rabb

**Affiliations:** 1 Department of Chemistry, University of Michigan, Ann Arbor, Michigan, USA; 2 Department of Chemistry, University of Massachusetts Boston, Boston, Massachusetts, USA

**Keywords:** Rieske oxygenase, enzyme catalysis, enzyme degradation, short-chain dehydrogenase reductase, enzyme kinetics, NAD, catabolism, pollutants, X-ray crystallography

## Abstract

Many microorganisms use both biological and nonbiological molecules as sources of carbon and energy. This resourcefulness means that some microorganisms have mechanisms to assimilate pollutants found in the environment. One such organism is *Comamonas testosteroni*, which metabolizes 4-methylbenzenesulfonate and 4-methylbenzoate using the TsaMBCD pathway. TsaM is a Rieske oxygenase, which in concert with the reductase TsaB consumes a molar equivalent of NADH. Following this step, the annotated short-chain dehydrogenase/reductase and aldehyde dehydrogenase enzymes TsaC and TsaD each regenerate a molar equivalent of NADH. This co-occurrence ameliorates the need for stoichiometric addition of reducing equivalents and thus represents an attractive strategy for integration of Rieske oxygenase chemistry into biocatalytic applications. Therefore, in this work, to overcome the lack of information regarding NADH recycling enzymes that function in partnership with Rieske non-heme iron oxygenases (Rieske oxygenases), we solved the X-ray crystal structure of TsaC to a resolution of 2.18 Å. Using this structure, a series of substrate analog and protein variant combination reactions, and differential scanning fluorimetry experiments, we identified active site features involved in binding NAD^+^ and controlling substrate specificity. Further *in vitro* enzyme cascade experiments demonstrated the efficient TsaC- and TsaD-mediated regeneration of NADH to support Rieske oxygenase chemistry. Finally, through in-depth bioinformatic analyses, we illustrate the widespread co-occurrence of Rieske oxygenases with TsaC-like enzymes. This work thus demonstrates the utility of these NADH recycling enzymes and identifies a library of short-chain dehydrogenase/reductase enzyme prospects that can be used in Rieske oxygenase pathways for *in situ* regeneration of NADH.

Rieske non-heme iron oxygenases (Rieske oxygenases) are key enzymes in microorganisms that are often involved in the assimilation of aromatic and polyaromatic hydrocarbons found in the environment (1, 2). These molecules arise from a combination of biological and nonbiological sources (3). Biological aromatic hydrocarbons are produced by plants and bacteria, and nonbiological aromatic hydrocarbons, or xenobiotics, are deposited into the environment from fuel, chemical, plastic, and industrial waste (3). Despite these different origins, both biological and nonbiological aromatic hydrocarbons are attractive sources of carbon and energy for the estimated 10^8^ to 10^12^ bacteria that inhabit each gram of soil (4, 5). As nonbiological hydrocarbons are typically more reduced than cellular organic material, degradative routes generally draw on oxygenase chemistry to oxidize, activate, and destabilize these molecules prior to subsequent degradation (Fig. 1) (2, 6).

**Figure 1:**
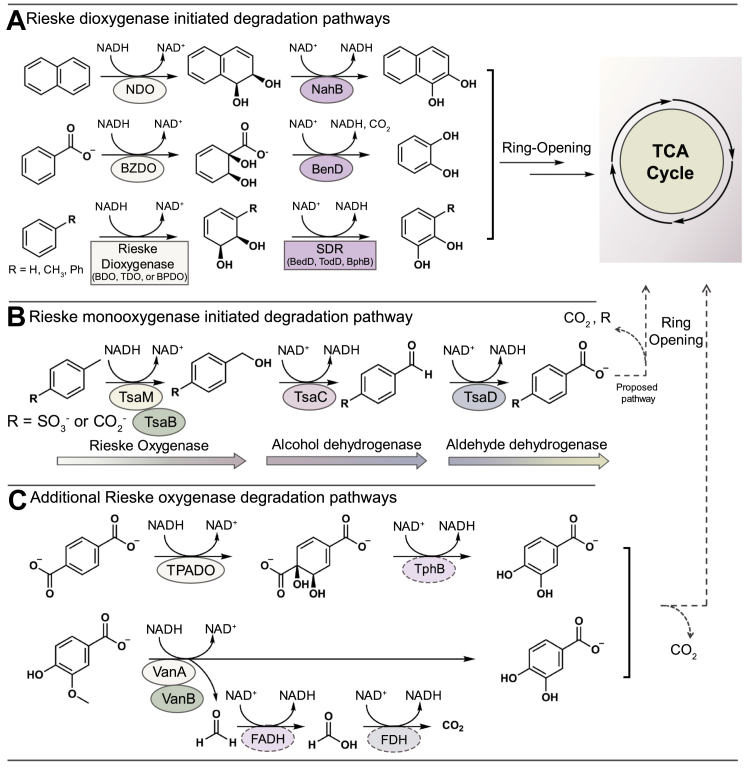
**Rieske oxygenases and SDR enzymes work together in a range of pathways that organisms use to degrade xenobiotic compounds found in the environment.***A*, Rieske dioxygenase–initiated pathways are used to degrade naphthalene, benzoate, benzene, toluene, and biphenyl. These pathways use Rieske oxygenases to generate *cis*-diols and SDR enzymes to form aromatic catechol intermediates that are subsequently cleaved and further metabolized in the TCA cycle (15, 16, 17, 18, 19, 20, 21). *B*, enzymes encoded in the *tsaMBCD* operon of *Comamonas testosteroni* are used to degrade 4-methylbenzenesulfonate and 4-methylbenzoate (7, 8, 9). This pathway starts with monooxygenation chemistry that is catalyzed by the Rieske oxygenase TsaM. The resultant alcohol-containing species is converted by two NAD^+^-dependent enzymes and eventually converted into protocatechuate. A series of additional steps allow for this molecule to eventually be degraded as described in (*A*). *C*, additional degradation pathways initiated by the Rieske oxygenases terephthalate dioxygenase (TPADO) and vanillate *O*-demethylase (VanA) metabolize terephthalic acid and vanillate, respectively. The TPADO-catalyzed reaction is similar to those described in (*A*) but instead employs an NAD^+^-dependent Zn^2+^-containing dehydrogenase (TphB), rather than an SDR enzyme (14). The VanA reaction has been successfully coupled with formaldehyde hydrogenase (FADH) and formate dehydrogenase (FDH) to regenerate NADH for additional Rieske oxygenase chemistry (11). SDR, short-chain dehydrogenase/reductase; TCA, tricarboxylic acid.

For example, a large number of Rieske oxygenases, in collaboration with a partner reductase protein, initiate degradation of aromatic compounds by performing a dioxygenation reaction on the aromatic ring (Fig. 1*A*) (2, 6). Other Rieske oxygenases involved in the degradation of alkyl arene compounds instead initiate catabolism by performing a monooxygenation reaction on the alkyl substituent to form an alcohol product (7, 8, 9). One such Rieske oxygenase is found in the organism *Comamonas testosteroni*, as part of the TsaMBCD pathway, which is involved in metabolizing 4-methylbenzenesulfonate and 4-methylbenzoate into protocatechuate (7, 8, 9). The first step in this catabolic pathway is a TsaM-catalyzed oxidation that transforms 4-methylbenzenesulfonate and 4-methylbenzoate into 4-(hydroxymethyl)benzenesulfonate and 4-(hydroxymethyl)benzoate, respectively (7, 8, 9) (Fig. 1*B*). This step requires two reducing equivalents that are delivered by the NADH-dependent reductase TsaB (7, 8, 9) (Fig. 1*B*). TsaC, an annotated short-chain dehydrogenase/reductase (SDR) enzyme, and TsaD, an annotated aldehyde dehydrogenase, are the next enzymes in the pathway. These proteins have been suggested to perform iterative NAD^+^-dependent oxidation reactions on the primary alcohol products of TsaM to produce 4-sulfobenzoate and 1,4-benzenedicarboxylate, respectively (7). Importantly, TsaC and TsaD also recycle the reducing equivalents used by TsaM and TsaB (Fig. 1*B*).

This elegant design, which integrates NADH-consuming and NADH-producing enzymes into a single pathway, eliminates the need for the addition of stoichiometric reducing equivalents, a currently expensive bottleneck in enzyme engineering (10, 11, 12). It is also a design that is emulated in other Rieske oxygenase–containing pathways involved in funneling diesel oils (13) and various aromatic xenobiotics, including the synthetic polymer poly(ethylene terephthalate) (14), into primary metabolism (Fig. 1). NAD(P)^+^-dependent alcohol and aldehyde dehydrogenases have also been proposed to operate alongside a Rieske oxygenase involved in alkane oxidation, but these proteins are yet to be characterized (13). Five additional SDR enzymes are known to function as *cis*-dihydrodiol dehydrogenases and participate in the degradation of naphthalene, benzoate, benzene, toluene, and biphenyl (15, 16, 17, 18, 19, 20, 21). These proteins are encoded in the *nah*, *ben*, *bed*, *tod*, and *bph* operons together with the Rieske oxygenases naphthalene dioxygenase (NDO), benzoate dioxygenase, benzene dioxygenase, toluene dioxygenase, and biphenyl 2,3-dioxygenase (BPDO) (15, 16, 17, 18, 19, 20, 21) (Fig. 1*A*). Likewise, although it performs a chemically distinct reaction from TsaC, a zinc- and NAD^+^-dependent dehydrogenase reductively transforms the dioxygenated *cis*-dihydrodiol product of the Rieske oxygenase terephthalate dioxygenase into protocatechuate (14) (Fig. 1*C*). Finally, related work has successfully demonstrated that formaldehyde dehydrogenase and formate dehydrogenase can be coupled to the VanA- and VanB-catalyzed *O*-demethylation of vanillate for NAD(P)H regeneration (11) (Fig. 1*C*).

Accordingly, enzymes like TsaC and TsaD provide an intriguing strategy from nature for applications aimed at using engineered microorganisms for environmental remediation. Our laboratory recently demonstrated the ability to engineer TsaM to catalyze divergent chemical transformations (22). However, to date, the low sequence identity among annotated SDR enzymes and the dearth of structurally characterized SDR enzymes that work in cooperation with Rieske oxygenases leave open questions regarding the utility of TsaC and TsaD, and other SDR enzymes in these types of Rieske oxygenase centered biocatalytic applications (23, 24). Similarly, the low percentage of structurally and experimentally characterized Rieske oxygenases (1, 25, 26, 27) only further complicates functional annotation of co-occurring SDR enzymes, and our ability exploit the chemistry of these naturally occurring pathways. Therefore, in this work, we undertook a structure–function analysis of TsaC. We solved a 2.18 Å resolution X-ray crystal structure of TsaC and determined that TsaC has a classical SDR fold that facilitates the oxidation of a broad range of substrates. Using this structure, differential scanning fluorimetry, and light scattering experiments, we identified key features involved in the TsaC-catalyzed reaction. By making rational active site mutations, we identified residues that dictate the substrate specificity of TsaC, and through *in vitro* reconstitution of the Tsa catabolic pathway, we show that NADH is efficiently recycled by TsaC and TsaD for oxygenase chemistry. Finally, through robust bioinformatic analyses of the SDR protein family, we identified a library of enzyme prospects that can be exploited in Rieske oxygenase pathways.

## Results

### TsaC is a classical SDR enzyme

**Figure 2:**
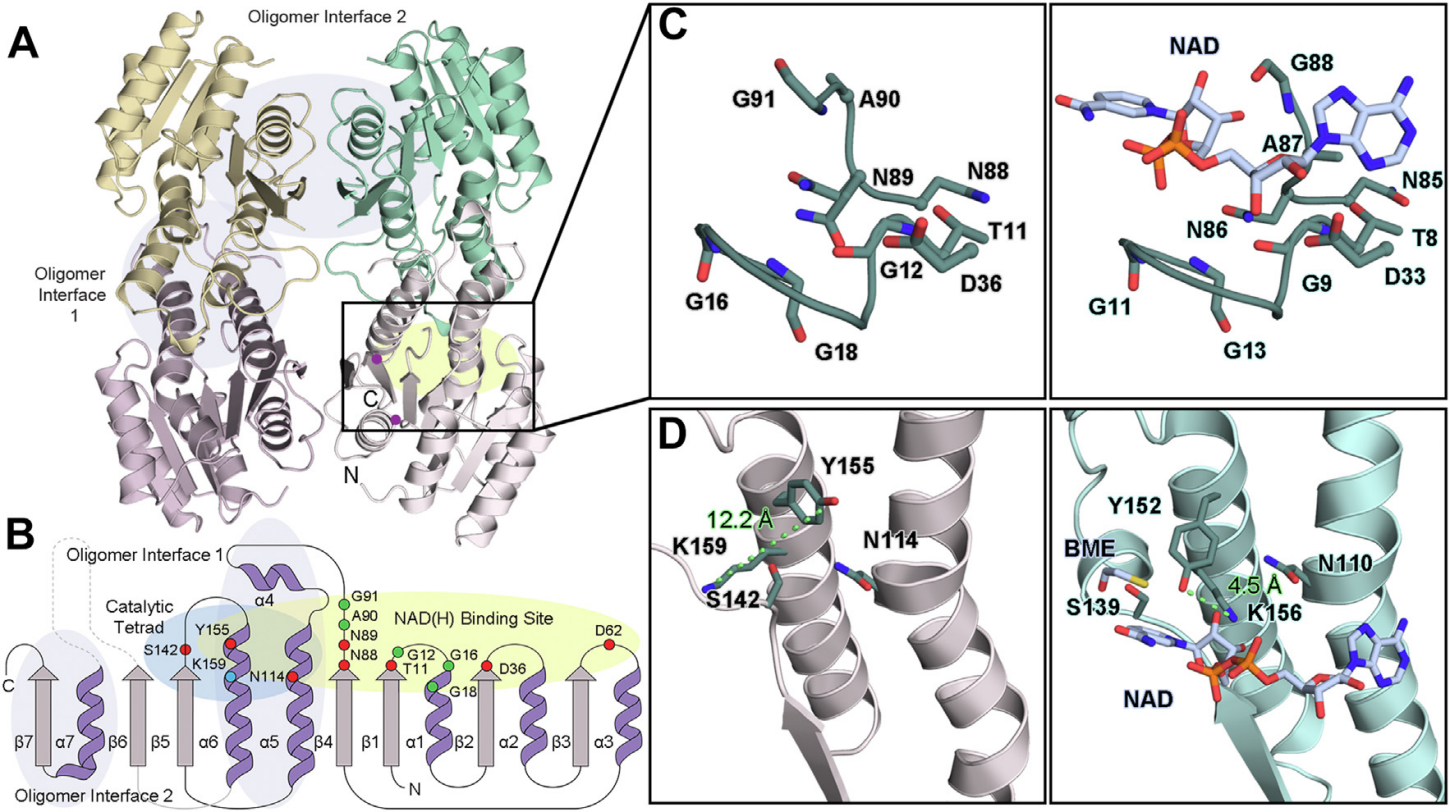
**TsaC has a classical SDR protein fold.***A*, TsaC has a tetrameric architecture. Each protomer of TsaC has a Rossmann fold. Two protomers of TsaC are found in the asymmetric unit (*yellow* and *pink*). Importantly, the dimeric interface of these two molecules maps to the previously identified oligomeric interface of *meso*-2,3-butanediol dehydrogenase (BDH) (28), which is formed predominantly between two long helices α5 and α6 of adjacent subunits. The second oligomeric interface is formed predominantly between α7 and β7 of adjacent subunits. *B*, a topology diagram of a monomeric unit of TsaC reveals the location of the catalytic tetrad residues (Asn114, Ser142, Tyr155, and Lys159) and the location of the residues involved in binding NAD^+^. This topology diagram also illustrates the secondary structure that forms the oligomeric interfaces and can be compared with (*A*). *C*, the TGX_3_GXG and NNAG motifs are used to bind NAD^+.^ These residues are found in TsaC (*left panel*) and can be compared with the equivalent residues in BDH (*right panel*, PDB ID: 1GEG (28)). *D*, the catalytic tetrad residues in TsaC (*left*) do not assume a catalytically competent orientation. In contrast, the structure of BDH, which contains both NAD^+^ and a β-mercaptoethanol (BME) inhibitor, is poised for chemistry to occur (*right*) (28). Regions that terminate because of disorder are indicated by *circles* in (*A*). BDH, *meso*-2,3-butanediol dehydrogenase; PDB, Protein Data Bank.

To determine the structure of TsaC, the enzyme was recombinantly expressed in *Escherichia coli* and isolated from cell lysate using affinity and gel filtration chromatography (Fig. S1). The crystal structure of TsaC was determined to a resolution of 2.18 Å using the structure of *meso*-2,3-butanediol dehydrogenase (BDH) as a model for molecular replacement (Figs. 2 and S2; Table S1) (28). In the solved structure, there are two copies of TsaC in the asymmetric unit, each of which showcases a Rossmann fold and contains a central seven-stranded β-sheet and seven α-helices (Fig. 2, *A* and *B*). Based on an approximate interface of 2837 Å^2^ (16.2% of the total monomeric surface area), these two subunits form a dimer, and like that observed in BDH, this dimer and two symmetry-related molecules assemble into a tetramer (Figs. 2*A* and S3). This homotetrameric oligomeric state is consistent with that observed using gel filtration chromatography (Fig. S1, *B* and *C*).

Similar to the structure of BDH (28), and as described for other SDR enzymes that are considered classical members of the family, within the Rossmann fold, TsaC contains the expected sequence motifs for binding a nicotinamide coenzyme (23, 24, 29). Namely, the coenzyme binding TGX_3_GXG motif residues between β1 and α1 are arranged in space as expected to provide room for binding either NAD(H) or NADP(H) (Figs. 2, *B* and *C*, S2, and S4). However, decorating this binding site, and following β2, is residue Asp36. This residue is positioned similarly to other negatively charged residues in SDR enzymes that are credited with favoring the use of NAD(H) (Figs. 2, *B* and *C* and S4) (30). Likewise, Asp62 is found to assume a similar spatial position to an Asp residue that in BDH and other SDR enzymes stabilizes NAD(H) binding through an interaction with the adenine ring (Fig. S4) (28, 29). The other classical motif, the NNAG motif, is also present in the structure of TsaC (Fig. 2*B*). It follows β4 and has been previously suggested to stabilize water in an otherwise hydrophobic pocket and to mediate proton transfer between bulk solvent and the catalytic tetrad of residues, Asn114, Ser142, Tyr155, and Lys159 (29).

Based on their traditional roles in other SDR enzymes, in this tetrad, Asn114, like the NNAG motif residues, also often participates in proton relay to the catalytically important Tyr155 residue (31). Ser142, on the other hand, should orient substrate in the active site for hydride transfer (Figs. 2*D*, S2, S5, and S6) (29). The remaining two residues, Tyr155 and Lys159, which are found in a YX_3_K sequence motif, should participate in acid–base chemistry. Tyr155 should function as a catalytic base, and its p*K*_a_ should be lowered though an electrostatic interaction with Lys159 (Figs. 2*D*, S2, S5, and S6) (29). However, in both chains of TsaC, the hydroxyl and amino functional groups of the Tyr155 and Lys159 side chains are located approximately 10 Å apart and do not appear to be catalytically competent (Figs. 2*D* and S6). This inactive conformation is a hallmark of other SDR enzymes that, like TsaC, do not have a nicotinamide coenzyme bound in the active site (Fig. 2*D*). In fact, a comparison with the active orientation of the catalytic tetrad residues in BDH (28) reveals that a substantial conformational change involving residues Ser142, Tyr155, Lys159, and the loop that connects β5 to α6 in TsaC would be required to accommodate NAD(H) (Figs. 2*B* and S7).

In agreement with the observed inactive state of the catalytic tetrad, approximately 30 residues between β6 and α7 are too disordered to build and are therefore missing from both monomers in the refined structure of TsaC (Fig. 2, *A* and *B*). This region maps to the so-called substrate-binding loop, which in other SDR enzymes typically folds into two antiparallel helices that lie adjacent to the catalytic tetrad when NAD(H) and substrate are present in the active site (Figs S8–S10) (21, 32). The inactive orientation of the catalytic residues and inability to model the substrate-binding loop in TsaC is thus consistent with the absence of NAD(H) and substrate. It also tracks with the proposed ordered “bi–bi” mechanistic proposal for SDR enzymes: NAD(H) binds to the protein first, substrate binds second, and the substrate-binding loop orders or closes to sequester chemistry away from solvent (Fig. S9) (23, 32). The ability to sample such open and closed states has been shown to rely on Pro residues that provide the needed rigidity for the substrate-binding loop to hinge toward and away from the active site (33). Indeed, a search of the Protein Data Bank (PDB) using the DALI server (34) identified several structures that superimpose well with TsaC but show marked disparities in the conformations of their substrate-binding loops (Fig. S9). A similar ensemble of substrate-binding loop orientations has been identified in biphenyl dehydrogenase (BphB): the loop is ordered when NAD^+^ and product are bound, disordered without product bound, and can even adopt an intermediate state (21). The equivalent of the hinge residues in TsaC are Pro185 and Pro221, which bookend the unresolved loop (Fig. S9). Pro185 is the last residue of β6 and is found in a PMX_3_T sequence of residues that is reminiscent of the previously reported PGX_3_T motif typically found in other classical SDR enzymes, and Pro221 is the first residue of α7 (Figs. S2, S9 and S10) (29). Aside from the substrate-binding loops, the proteins identified by the DALI server all showcase the characteristic classical TGX_3_GXG and NNAG motifs, and intriguingly, represent proteins that use NAD(H) or NAD(P)H in their reactions (Figs. S9 and S10).

### TsaC performs chemistry on 4-(hydroxymethyl)benzenesulfonate and 4-(hydroxymethyl)benzoate

**Figure 3:**
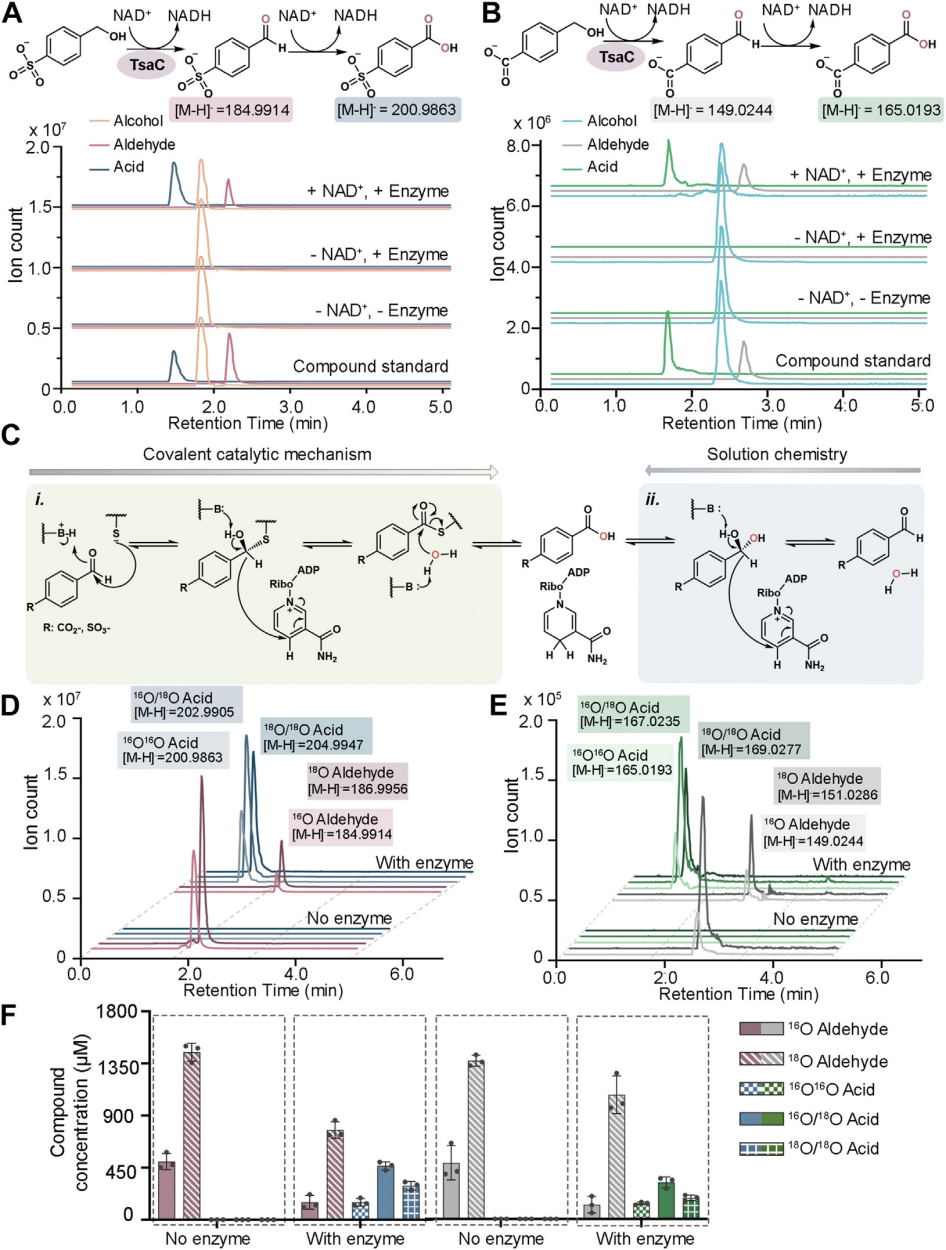
**TsaC catalyzes the NAD**^**+**^**-dependent formation of aldehyde (4-formylbenzenesulfonate and 4-formylbenzoate) and carboxylic acid (4-sulfobenzoate and 1,4-benzenedicarboxylate) products.***A*, LC–MS reveals that TsaC catalyzes the conversion of 4-(hydroxymethyl)benzenesulfonate into 4-formylbenzenesulfonate and 4-sulfobenzoaote in an NAD^+^-dependent manner. *B*, TsaC also accepts 4-(hydroxymethyl)benzoate as a substrate and mediates its conversion into 4-formylbenzoate and 1,4-benzenedicarboxylate. *C*, two proposals regarding how the 4-sulfobenzoate and 1,4-benzenedicarboxylate products are formed. The first (i) proposal is enzyme mediated and requires an active site Cys residue to form a thiohemiacetal intermediate. The second (ii) proposal involves the formation of the aldehyde product, its conversion into an aldehyde hydrate, and a TsaC- and NAD^+^-dependent oxidation. *D*, incubation of 4-formylbenzenesulfonate in ^18^O-labeled water allows for LC–MS detection of labeled 4-formylbenzenesulfonate (no enzyme). Similarly, performing the TsaC reaction in ^18^O-labeled water results in a distribution of labeled and unlabeled aldehyde and carboxylic acid products that can be detected using LC–MS. *E*, like that described in (*D*), the addition of ^18^O-labeled water to 4-formylbenzoate results in the nonenzymatic incorporation of ^18^O into 4-formylbenzoate. Labeling is also observed in the aldehyde and carboxylic acid products when TsaC catalyzes its reaction in ^18^O-labeled water. *F*, a bar graph of the data from (*D*) reveals the different concentrations of the products generated when the TsaC-catalyzed reactions are performed in ^18^O-labeled water. These data suggest that the aldehyde hydrate made in solution is also a substrate of TsaC. Here, the amounts of 4-formylbenzenesulfonate, 4-sulfobenzoate, 4-formylbenzoate, and 1,4-benzenedicarboxylate produced in the reactions are illustrated in *salmon*, *blue*, *gray*, and *green colors*, respectively. In this panel, data were measured using n = 3 independent experiments and are presented as the mean value ± SD of these measurements. For (*D* and *E*), a reaction scheme that details how the different species of ^18^O-labeled compounds are generated is included in Fig. S16.

The activity of the recombinantly expressed and purified TsaC was tested against both reported (7, 8, 9) native substrates, 4-(hydroxymethyl)benzenesulfonate and 4-(hydroxymethyl)benzoate, using LC–MS experiments at a range of pH values (Fig. S11). To our surprise, it was determined that the combination of TsaC with NAD^+^ and 4-(hydroxymethyl)benzenesulfonate results in formation of two molecules that have the same mass and retention time as commercially purchased standards of the expected aldehyde (4-formylbenzenesulfonate) product as well as an additional carboxylic acid (4-sulfobenzoate) species (Figs. 3*A* and S12). Similar formation of two products is also observed when TsaC is incubated with NAD^+^ and 4-(hydroxymethyl)benzoate (Figs. 3*B* and S13). As expected based on the structural motifs described previously, no formation of 4-formylbenzenesulfonate, 4-sulfobenzoate, 4-formylbenzoate, or 1,4-benzenedicarboxylate is detected when NADP^+^ is used rather than NAD^+^, and no activity is observed when a Y155F variant of TsaC is exchanged for the wild-type enzyme in the reactions (Figs. 2*C*, S4, S14, and S15; Table S2).

To investigate the origin of the detected 4-sulfobenzoate and 1,4-benzenedicarboxylate products, additional experiments on TsaC were performed. Typically, in biology, aldehyde compounds like 4-formylbenzenesulfonate and 4-formylbenzoate are converted into carboxylic acid–containing compounds such as 4-sulfobenzoate and 1,4-benzenedicarboxylate by aldehyde dehydrogenase enzymes like TsaD (35). Members of the aldehyde dehydrogenase enzyme class contain an active site Cys residue that initiates catalysis *via* formation of a thiohemiacetal intermediate. Formation of this species facilitates hydride transfer from the aldehyde substrate to NAD^+^ (35) (Fig. 3*C*). In lieu of TsaC containing such an active site Cys residue, it was hypothesized, based on the well-known ability of aldehydes to react with water (36, 37, 38), that the 4-sulfobenzoate and 1,4-benzenedicarboxylate products were formed using a combination of solution and enzyme-mediated chemistry. Specifically, the addition of water to 4-formylbenzenesulfonate and 4-formylbenzoate produces aldehyde hydrates that are oxidized in an NAD^+^- and TsaC-dependent reaction (Fig. 3*C*). This type of water-mediated reaction has been previously described for horse liver alcohol dehydrogenase, which converts both ethanol and octanol into their corresponding aldehyde and carboxylic acid compounds with similar efficiencies (36).

To evaluate whether TsaC similarly performs this type of water-mediated sequential oxidation reaction to produce 4-sulfobenzoate and 1,4-benzenedicarboxylate, the identity of the reaction products following incubation in ^18^O-labeled water was investigated. Here, it was first determined that some nonenzymatic incorporation of ^18^O into 4-formylbenzenesulfonate and 4-formylbenzoate could be detected following incubation in ^18^O-labeled water (Figs. 3 and S16). Likewise, combination of TsaC, NAD^+^, and 4-(hydroxymethyl)benzenesulfonate or 4-(hydroxymethyl)benzoate in ^18^O-labeled water leads to formation of differentially labeled aldehyde- and carboxylic acid–containing products (Figs. 3, *D*–*F* and S16). These molecules presumably arise from the equilibrium between the aldehyde and aldehyde hydrate and suggest that, as described for horse liver alcohol dehydrogenase, the active site of TsaC is able to capture and oxidize the aldehyde hydrate forms of 4-formylbenzenesulfonate and 4-formylbenzoate (Fig. S16).

Based on this finding, the competence of TsaC in catalyzing these two different oxidation reactions was compared by measuring total turnover numbers (TTNs) and steady-state kinetic parameters (Figs. S17–S19 and Table 1). For both the sulfonate- and carboxylate-containing substrates, higher turnover numbers are observed with the alcohol, relative to the aldehyde, species (Fig. S17). Analogously, for both 4-(hydroxymethyl)benzenesulfonate and 4-(hydroxymethyl)benzoate, the measured *K*_*M*_ values are lower than that measured with 4-formylbenzenesulfonate and 4-formylbenzoate, meaning that the catalytic efficiency of TsaC with the alcohol substrates is greater than that observed with the aldehyde compounds (Figs. S18 and S19; Table 1).

**Table 1:** Summary of kinetic parameters for TsaC and TsaD with the two reported native substrates

Substrate	*K*_*M*_ (μM)	*k*_cat_ (min^−1^)	*V*_max_ (μM/min)	*k*_cat_/*K*_*M*_ (M^−1^ s−^1^)
TsaC and 4-(hydroxymethyl)benzenesulfonate	13 ± 2.0	6.0 ± 0.24	12 ± 0.49	7900 ± 1300
TsaC and 4-formylbenzenesulfonate	20 ± 3.5	7.5 ± 0.35	15 ± 0.71	6200 ± 1100
TsaC and 4-(hydroxymethyl)benzoate	18 ± 2.8	5.9 ± 0.22	12 ± 0.44	5400 ± 870
TsaC and 4-formylbenzoate	23 ± 4.7	6.7 ± 0.40	13 ± 0.81	4800 ± 1000
TsaD and 4-formylbenzenesulfonate	16 ± 2.5	6.7 ± 0.31	13 ± 0.63	6900 ± 1100
TsaD and 4-formylbenzoate	21 ± 6.9	6.3 ± 0.59	13 ± 1.2	5000 ± 1700

**Figure 4:**
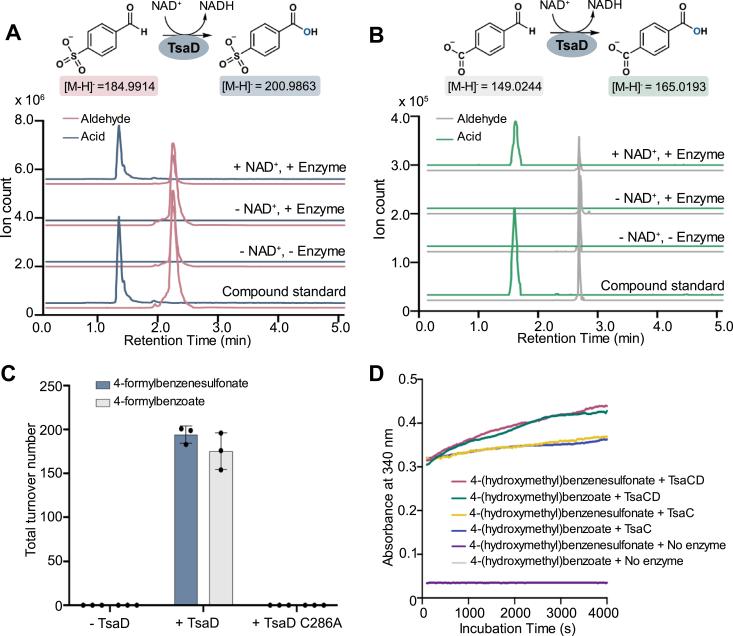
**TsaD catalyzes the NAD**^**+**^**-dependent conversion of 4-formylbenzenesulfonate and 4-formylbenzoate into 4-sulfobenzoate and 1,4-benzenedicarboxylate, respectively.***A*, LC–MS reveals that TsaD is active and converts 4-formylbenzenesulfonate into 4-sulfobenzoate. This reaction requires NAD^+^. *B*, TsaD also accepts and catalyzes an NAD^+^-dependent oxidation of 4-formylbenzoate. *C*, a C286A variant of TsaD is not active on either of the reported (7, 8, 9) native substrates. In this panel, data were measured using n = 3 independent experiments and are presented as the mean value ± SD of these measurements. *D*, as indicated by an increase in the absorbance at 340 nm, NADH is produced in the TsaC- and TsaD-catalyzed transformation of 4-(hydroxymethyl)benzenesulfonate and 4-(hydroxymethyl)benzoate into 4-sulfobenzoate and 1,4-benzenedicarboxylate, respectively. In the measured time frame, the amount of NADH produced in this enzyme cascade is more than that produced in the TsaC-containing reactions on the same substrates. Control reactions with no enzyme overlap just above baseline in (*D*).

To further investigate the relevance of the TsaC-mediated production of 4-sulfobenzoate and 1,4-benzenedicarboxylate, TsaD, which is proposed to catalyze the *in vivo* formation of these compounds, was recombinantly expressed and purified (Fig. S20). Once produced, the ability of TsaD to catalyze formation of 4-sulfobenzoate and 1,4-benzenedicarboxylate was probed. Here, it was determined that TsaD also transforms 4-formylbenzenesulfonate and 4-formylbenzoate into 4-sulfobenzoate and 1,4-benzenedicarboxylate, respectively, using an NAD^+^-dependent reaction mechanism (Figs. 4, *A* and *B*, S21, and S22). The catalytic efficiency of the TsaD-catalyzed reactions is similar, albeit slightly greater, than that observed with TsaC (Figs. 4*C*, S23, and S24; Table 1). However, TsaD should not need to rely on solution chemistry to produce the carboxylic acid products, unlike TsaC. Instead, TsaD should proceed using a catalytic Cys residue (Figs. 4*C* and S25). Indeed, a C286A variant of TsaD is incapable of performing chemistry on either 4-formylbenzenesulfonate or 4-formylbenzoate (Figs. 4*C* and S26; Table S2). Collectively, these results suggest that although TsaC can generate a population of 4-sulfobenzoate and 1,4-benzenedicarboxylate products, TsaD is more proficient in making these molecules. Likewise, the endpoint amount and rate of NADH produced in reactions that contain both TsaC and TsaD is amplified relative to that observed in reactions that contain only TsaC (Fig. 4*D*).

### Identification of residues that dictate the substrate specificity of TsaC

**Figure 5:**
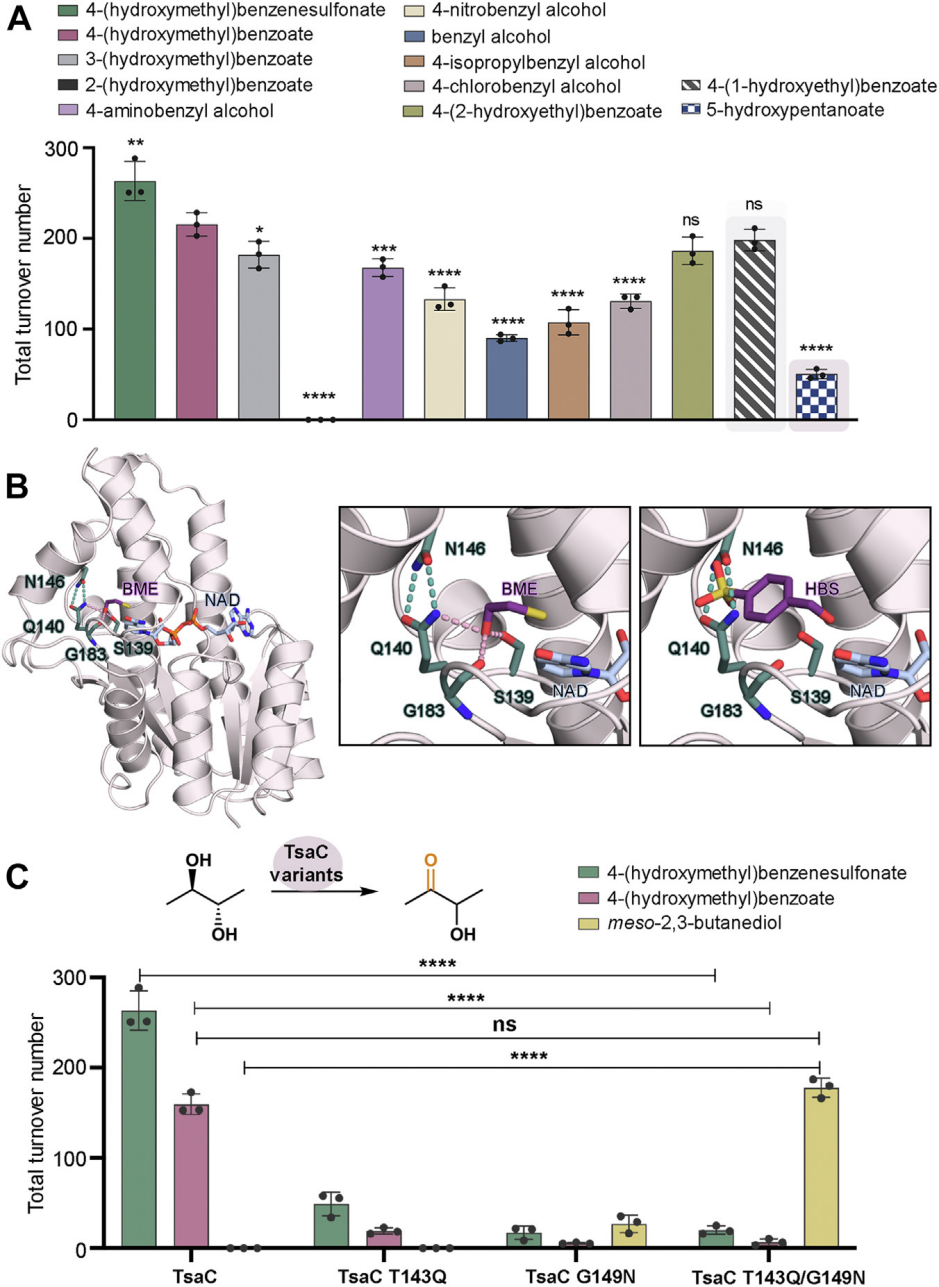
**TsaC oxidizes a variety of substrates and can be engineered to perform chemistry on *meso*-2,3-butanediol.***A*, the total turnover number of TsaC was measured with a plethora of substrates. This analysis revealed that TsaC has a broad substrate scope. The substrates highlighted in *gray* and *pink* are the tested secondary and linear alcohol substrates. *B*, the active site of BDH contains a competitive inhibitor (β-mercaptoethanol, BME). BME makes several key interactions in the active site, including one with Gln140. The position of BME was used to model how 4-(hydroxymethyl)benzenesulfonate (HBS) would sit in the active site of BDH. This modeling experiment reveals that the larger aromatic substrate of TsaC clashes with the “gating” residues Gln140 and Asn146. *C*, the equivalent residues in TsaC, Thr143, and Gly149 were mutated into their BDH counterparts. The resultant single and double variants of TsaC have decreased activity on the native 4-(hydroxymethyl)benzenesulfonate and 4-(hydroxymethyl)benzoate substrates. At the same time, these variants showcase a significantly higher level of activity on *meso*-2,3-butanediol. In (*A* and *C*), data were measured using n = 3 independent experiments and are presented as the mean value ± SD of these measurements. In this figure, ∗∗∗∗*p* < 0.0001, ∗∗∗*p* < 0.001, ∗∗*p* < 0.01, and ns indicates no significant difference from an ordinary one-way ANOVA Dunnett analysis (*A*) and two-way ANOVA Tukey analysis (*C*). BDH, *meso*-2,3-butanediol dehydrogenase.

Inspired by the similar kinetic parameters measured for TsaC in the presence of 4-(hydroxymethyl)benzenesulfonate and 4-(hydroxymethyl)benzoate substrates, an investigation into the range of substrates that TsaC could accept was launched (Table S3). Here, it was investigated whether TsaC could oxidize substrates that contain a hydroxymethyl functional group at the C3 position or C2 position of the aromatic ring rather than at the C4 position (3-(hydroxymethyl)benzoate and 2-(hydroxymethyl)benzoate) (Figs. S27 and S28). In addition, it was investigated whether TsaC could oxidize substrates that contain different functional groups at the C1 position (4-aminobenzyl alcohol, 4-nitrobenzyl alcohol, benzyl alcohol, 4-isopropylbenzyl alcohol, and 4-chlorobenzyl alcohol) or a longer carbon chain at the C4 position (4-(2-hydroxyethyl)benzoate) (Figs. S29–S34). The ability of TsaC to perform chemistry on a secondary (4-(1-hydroxyethyl)benzoate), rather than a primary, alcohol substrate, and a linear (5-hydroxypentanoate), rather than an aromatic substrate, was also probed (Figs. 5*A*, S35, and S36). In these experiments, it was determined that whereas TsaC accepts and oxidizes 3-(hydroxymethyl)benzoate, it shows no activity on 2-(hydroxymethyl)benzoate (Figs. 5*A*, S27, and S28). This result agrees with previous studies that indicate the upstream Rieske oxygenase, TsaM, only performs mono-oxygenation chemistry on 3-methylbenzoate and not 2-methylbenzoate (22, 39). In addition, it was determined that the active site of TsaC accommodates aromatic substrates with a variety of functional groups at C1, albeit with a slight preference for 4-aminobenzyl alcohol relative to the other provided options (Figs. 5*A* and S29). Quite surprisingly, the TTNs of TsaC on 4-(2-hydroxyethyl)benzoate and 4-(1-hydroxyethyl)benzoate show no significant difference relative to those observed with a 4-(hydroxymethyl)benzoate substrate (Figs. 5*A*, S34, and S35). In contrast, the lowest detectable TTN of TsaC is observed with 5-hydroxypentanoate, which suggests that the active site of TsaC is built to house aromatic, rather than linear, substrates (Figs. 5*A* and S36).

Indeed, despite the scope of disparate substrates that are oxidized by TsaC, it was determined that the small four-carbon substrate of BDH, *meso*-2,3-butanediol, is not oxidized by TsaC (Figs. 5 and S37). To better understand at the molecular level the inability of TsaC to transform *meso*-2,3-butanediol into 3-hydroxy-2-butanone, the crystal structure of BDH with a competitive inhibitor, β-mercaptoethanol, bound in the active site was analyzed (28). In this structure, the thiol moiety of β-mercaptoethanol interacts with the Ser and Tyr residues from the catalytic tetrad. The hydroxyl group, on the other hand, interacts with the side chain and backbone of Gln140 and Gly182, respectively (Figs. 5*B* and S38) (28). Gln140, in addition to interacting with the BDH substrate, also forms a salt bridge with Asn146 that appears to gate the active site and lock β-mercaptoethanol into place (Fig. S38). In this binding pose, the thiol group of β-mercaptoethanol mimics the C2 position of *meso*-2,3-butanediol and clearly displays how the substrate must be oriented for oxidation (28). Interaction of C2 with the catalytic Tyr residue allows for deprotonation and subsequent hydride transfer to NAD^+^ (Figs. 5*B* and S38).

Using the position of β-mercaptoethanol as a guide, both reported (7, 8, 9) native substrates of TsaC were superimposed into the active site of BDH (Figs. 5*B* and S39). This analysis revealed that the side chains of the “gating” residues, Gln140 and Asn146, which are located between β5 and α7 directly following the catalytic Ser residue, severely clash with the large sizes of the aromatic TsaC substrates (Figs. 5*B* and S39). As previous studies have indicated that Gln140 and Asn146 are important dictators of substrate specificity in BDH (28, 40), the roles of the equivalent residues in TsaC, Thr143, and Gly149, were investigated. To evaluate whether Thr143 and Gly149 govern the substrate specificity of TsaC, three variants (T143Q, G149N, and T143Q/G149N) of TsaC were made using site-directed mutagenesis (Table S2). These variants were expressed, purified, and assayed using 4-(hydroxymethyl)benzenesulfonate, 4-(hydroxymethyl)benzoate, and *meso*-2,3-butanediol substrates (Figs. 5*C* and S40). In this venture, it was determined that relative to wild-type TsaC, all three variants had significantly reduced activity on 4-(hydroxymethyl)benzenesulfonate and 4-(hydroxymethyl)benzoate (Fig. 5*C*). For the non-native *meso*-2,3-butanediol substrate, activity was monitored using GC–MS, rather than LC–MS. These GC–MS experiments revealed that whereas the T143Q variant shows no ability to oxidize *meso*-2,3-butanediol, both the G149N and T143Q/G149N variants of TsaC show a significantly improved ability to transform *meso*-2,3-butanediol into 3-hydroxy-2-butanone (Figs. 5*C* and S37). Notably, the double variant with *meso*-2,3-butanediol shows no significant difference in TTN relative to that measured for wild-type TsaC with a 4-(hydroxymethyl)benzoate substrate (Figs. 5*C* and S37). This result shows that the rational residue switch made in TsaC allows for *meso*-2,3-butanediol to bind in the active site and be oxidized. Furthermore, these results suggest that the residues in the “gating” positions are intricately linked to the substrate specificity of TsaC.

### Alterations in the thermal stability of TsaC are consistent with a substrate-mediated conformational change

Previous studies have revealed that the binding of a ligand to a protein can induce measurable changes in thermal stability, and other studies indicate that SDR enzymes undergo conformational changes in response to substrate and coenzyme binding (21, 41, 42, 43, 44). In this work, it was investigated whether the melting temperature (*T*_m_), measured using differential scanning fluorimetry experiments, could be used to probe the existence of such structural changes, or ordering of the protein (Fig. 5 and Table 2). As a baseline for these experiments, and for comparison to that previously measured for TsaM (53.7 °C ± 0.5 deg. C), the thermal stability of TsaC was measured (22). Here, it was determined that wild-type TsaC has a *T*_m_ of 54.7 °C ± 0.02 deg. C (Fig. S41 and Table S4). This *T*_m_ value is consistent within the concentration range of 5 to 50 μM but is decreased (49.0 °C ± 0.5 deg. C) at a higher concentration of 500 μM. This change, as described later, based on size-exclusion multiangle light scattering (SEC–MALS) and dynamic light scattering (DLS) experiments, is likely because of the reversible formation of higher-order oligomeric species (Figs. S41 and S42).

**Table 2:** Melting temperatures (*T*_m_) of Y155F TsaC determined using thermal shift assays

All variant Δ*T*_m_ values were calculated relative to the values measured for Y155F TsaC, and T143Q/G149N TsaC highlighted in *gray*.

Using the collected information about concentration as a starting point, a titration of NAD^+^ into 10 μM TsaC was performed (Fig. S41*B*). In this experiment, it was determined that the *T*_m_ remains relatively constant as the amount of NAD^+^ added to TsaC is increased (Fig. S41). Subsequent NAD^+^ titration measurements performed on TsaC in the presence of a 100-fold excess of the 4-(hydroxymethyl)benzoate substrate instead reveal that the observed *T*_m_ amplifies as NAD^+^ concentration is increased (Fig. S41 and Table S4). This result indicates that the combination of NAD^+^ and 4-(hydroxymethyl)benzoate has a stabilizing effect on the structure of TsaC and is consistent with the proposed ordered “bi–bi” mechanism for SDR enzymes (23). Specifically, formation of a more ordered substrate-binding loop following binding of NAD^+^ and 4-(hydroxymethyl)benzoate in the TsaC active site is presumably linked to the observed increase in thermal stability (Fig. S41). However, in these experiments, since wild-type TsaC can perform chemistry on 4-(hydroxymethyl)benzoate, the measured *T*_m_ values likely reflect an ensemble of conformational states encountered during catalysis (Table S4).

As such, a comparable set of *T*_m_ values were also measured using the inactive Y155F TsaC variant, for which 4-(hydroxymethyl)benzoate and NAD^+^ can be considered as binding ligands, rather than substrates (Figs. S43–S45 and Table 2). This variant has a *T*_m_ of 51.7 °C ± 0.7 deg. C. Similar to that described for the wild-type enzyme, increasing amounts of NAD^+^ do not markedly influence this value (Fig. S43 and Table 2). In contrast, the combined addition of NAD^+^ and 4-(hydroxymethyl)benzoate or 4-(hydroxymethyl)benzenesulfonate to this variant results in an approximate 4 °C increase in *T*_m_. Again, this result supports the notion that the substrate-binding loop transitions into a more ordered state or closed state in the presence of NAD^+^ and substrate (Figs. S43 and S44; Table 2). In both sets of experiments performed on the native substrates, the *T*_m_ drops back to that of the apo-Y155F TsaC variant in the presence of NAD^+^ and 4-formylbenzeneseulfonate, 4-formylbenzoate, 4-sulfobenzoate, and 1,4-benzenedicarboxylate (Figs. S43 and S44; Table 2). The same 3 to 4 °C increase in *T*_m_ is observed when Y155F TsaC is incubated with NAD^+^ and 4-aminobenzyl alcohol, benzyl alcohol, 4-(2-hydroxyethyl)benzoate, or 4-(1-hydroxyethyl)benzoate substrate, which all support TsaC activity (Figs. 5, S43, and S44; Table 2).

In a separate experiment, it was determined that the use of 2-(hydroxymethyl)benzoate, a molecule that is not oxidized by TsaC, in the assay did not elicit an increase in the thermal stability of the Y155F TsaC variant in the presence of NAD^+^ (Figs. 5 and S45; Table 2). A similar lack of improved thermal stability is also observed when wild-type TsaC is combined with *meso*-2,3-butanediol and NAD^+^ (Fig. S46 and Table S4). The 4-chlorobenzyl alcohol and 5-hydroxypentanoate molecules also do not perturb the *T*_m_ of Y155F TsaC, but these substrates are oxidized into their corresponding products (Figs. 5, and S45; Table 2). A similar phenomenon is also observed in the combinations that include the T143Q/G149N variant of TsaC, NAD^+^, and *meso*-2,3-butanediol, and Y155F TsaC with NAD^+^ and either 4-formylbenzenesulfonate and 4-formylbenzoate substrates, which in this work, we have shown can be hydrated and subsequently oxidized by TsaC (Figs. 3, S43, and S44; Table 2).

Additional thermal shift data were also measured in the presence of NADP^+^ as well as the reduced coenzyme NADH and a small selection of substrates or products. In alignment with the structural results and activity measurements that show NADP^+^ is unable to support TsaC activity, it was determined that the *T*_m_ of Y155F TsaC when incubated with NADP^+^ and either of the native substrates is unchanged (Figs. 2*B*, S4, S14, S39, and S47). Notably, incubation of wild-type TsaC with NADH elicits a 12.4 °C increase in thermal stability of the protein (Figs. S41 and S47; Table S4). Using these data as a foundation, the *T*_m_ of the inactive Y155F TsaC variant in the presence of NADH, NADH 4-(hydroxymethyl)benzoate, NADH and 4-formybenzoate, or NADH and 1,4-benzenedicarboxylate was subsequently measured. Interestingly, the *T*_m_ values measured for mixtures that contained Y155F TsaC with NADH alone or with NADH and 4-(hydroxymethyl)benzoate or 1,4-benzenedicarboxylate were approximately 18 °C higher than that measured for the sample that contained only Y155F TsaC (Table 2). Intriguingly, incubation with NADH and 4-formylbenzoate decreases the *T*_m_ back to that of the enzyme alone (Fig. S47 and Table 2). Collectively, these results demonstrate that NADH imparts thermal stability to TsaC unless the partner product of NADH, 4-formylbenzoate, is also present.

To further assess if the changes in thermal stability are because of substrate-mediated conformational changes rather than potential changes in oligomeric state brought about by the binding of substrate, product, or coenzyme, SEC–MALS and DLS experiments were conducted (Fig. S48 and Table S5). The SEC–MALS experiments were run with 90 μM of the Y155F TsaC variant in the presence of either NAD^+^ or NADH and reveal the presence of multiple species that each have a molar mass that is most consistent with a tetramer (Table S5). These species presumably correspond to different tetrameric conformations that interact with the column differently. Protein eluting at the apex of the largest peak has a calculated concentration of approximately 0.3 μM, which is lower than the concentrations used for the enzyme assays (5–10 μM) and *T*_m_ analysis (10 μM), suggesting that the oligomeric state will be consistently tetrameric in all examined conditions. To supplement these experiments and calculate hydrodynamic radius (*R*_h_) values, cuvette-based DLS experimental data were collected with different concentrations of Y155F TsaC, and in the presence of NAD^+^, NAD^+^ and substrate, or product and NADH (Fig. S48). This set of experiments also reveals that under the tested conditions, and until concentrations of at least 200 μM, the tetramer remains as the predominant oligomer with an *R*_h_ value around 53 Å (Fig. S48).

### TsaC and TsaD regenerate NADH to support Rieske oxygenase chemistry

**Figure 6:**
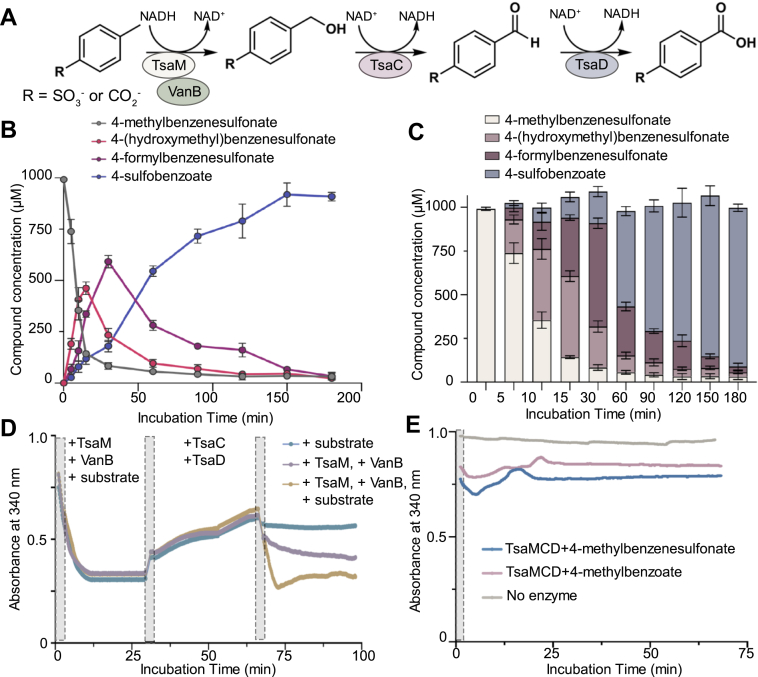
**The combination of TsaM, VanB, TsaC, and TsaD with 4-methylbenzenesulfonate results in the iterative formation of 4-(hydroxymethyl)benzenesulfonate, 4-formylbenzenesulfonate, and 4-sulfobenzoate.***A*, reaction scheme that details the TsaM-, VanB-, TsaC-, and TsaD-mediated degradation of 4-methylbenzenesulfonate and 4-methylbenzoate. *B*, in this enzyme cascade experiment, 4-methylbenzenesulfonate is consumed over time and iteratively converted into the corresponding alcohol, aldehyde, and carboxylic acid products. *C*, the data from (*B*) are plotted as a bar graph to reveal the mass balance of the different products formed over time. Additional mass balance data for the movement of 4-methylbenzoate through the pathway is shown in Fig. S52. *D*, NADH is consumed in assays that contain TsaM, VanB, NADH, and 4-methylbenzenesulfonate. Addition of TsaC and TsaD to this NADH-consuming reaction allows for NADH production (TsaC and TsaD were injected at the time point marked by the *second gray rectangle*). Likewise, addition of 4-methylbenzenesulfonate, TsaM and VanB, or TsaM, VanB, and 4-methylbenzenesulfonate at the 60 min mark promotes NADH consumption. *E*, compared with *D*, a reaction that contains 4-methylbenzenesulfoante or 4-methylbenzoate with TsaM, VanB, TsaC, and TsaD shows only minor fluctuations of NADH through the duration of the experiment. In (*D* and *E*), the *gray rectangles* indicate when different reaction components are added. The data in (*B* and *C*) were measured using n = 3 independent experiments and are plotted as the average ± SD of those data points.

To evaluate whether the above described production of NADH in the TsaC- and TsaD-catalyzed reactions could support continual activity of TsaM, both TsaM and the reductase protein VanB were purified (Fig. S49). VanB was purified for use in these experiments because we previously discovered that VanB supports a significantly higher level of TsaM activity than the native reductase TsaB (22, 39). Here, it was determined that the combination of TsaM, VanB, TsaC, TsaD, NADH, and either 4-methylbenzenesulfonate or 4-methylbenzoate allows for the iterative detection of the expected alcohol (4-(hydroxymethyl)benzenesulfonate or 4-(hydroxymethyl)benzoate), aldehyde (4-formylbenzenesulfonate or 4-formylbenzoate), and carboxylic acid (4-sulfobenzoate or 1,4-benzenedicarboxylate) products using LC–MS (Fig. 6, *A*–*C*, S50, and S51). In these assays, using a mass balance experiment, it was determined that whereas the 4-methylbenzenesulfonate substrate is essentially consumed within 90 min, the 4-methylbenzoate substrate persists (Figs. 6*C* and S52). This result indicates that whereas TsaC and TsaD do not appear to have a strong preference for one of the proposed native substrates over the other, as previously reported (22), TsaM and VanB consume more 4-methylbenzenesulfonate than 4-methylbenzoate (Figs. 6*C* and S52).

In complementary experiments, NADH consumption, rather than product formation, was monitored at 340 nm for 100 min (Figs. 6*D*, S53, and S54). In reactions that contain TsaM, VanB, NADH, and either 4-methylbenzenesulfonate or 4-methylbenzoate, a sharp decrease in NADH availability occurs in the first 30 min of the reaction (Figs. 6*D*, S53, and S54). Again, despite reaching saturation at a similar time point, more NADH is consumed in assays that contain 4-methylbenzenesulfonate rather than 4-methylbenzoate (Figs. 6*D*, S53, and S54). Nevertheless, for both experiments, at the 30 min time point, addition of both TsaC and TsaD into the enzyme cascade results in regeneration of NADH (Figs. 6*D*, S53, and S54). The amount of NADH produced in this step, in agreement with the measured kinetic parameters for TsaC and TsaD, again shows less of a dependence on substrate identity. Furthermore, as expected, the amount of NADH recycled is amplified in the presence of both TsaC and TsaD relative to TsaC alone (Figs. 6*D*, S53, and S54). Supplementation of additional substrate, TsaM and VanB, or TsaM, VanB, and substrate into the reaction mixture at the 60 min time point allows for reuse of NADH to different extents (Figs. 6*D* and S54). The highest level of NADH reuse occurs when all three components, TsaM, VanB, and substrate, are added into the reactions, and the lowest reuse of NADH occurs with just the addition of substrate. Finally, in an experiment where all four proteins, NADH, and either 4-methylbenzenesulfonate or 4-methylbenzoate are incubated together for the duration of the experiment, following an initial decrease in the amount of NADH, the fluctuation in NADH concentration during the catalytic cycle is approximately unchanged (Fig. 6*E*). This result suggests that the cycle becomes redox neutral: NADH that is consumed by TsaM and VanB is recycled by TsaC and TsaD and subsequently reused. In whole, this cycle supports the TsaM-, VanB-, TsaC-, and TsaD-catalyzed catabolism of the xenobiotic 4-methylbenzenesulfonate as well as 4-methylbenzoate.

### Bioinformatic analyses reveal a wealth of SDR enzymes that co-occur with Rieske oxygenases

**Figure 7:**
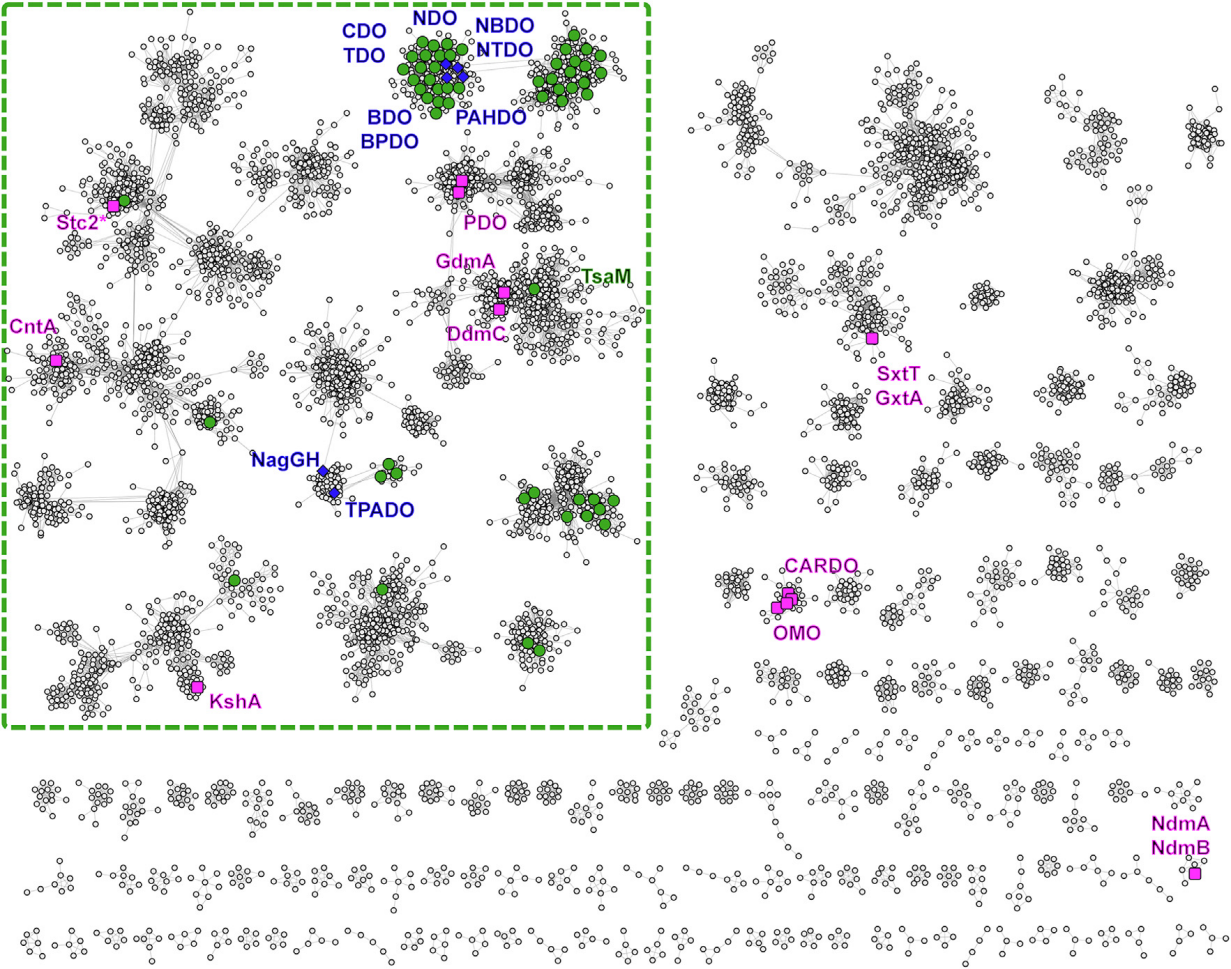
**SDR enzymes co-occur with a variety of Rieske oxygenases.** Here, a UniRef50 sequence similarity network (SSN) of the Rieske oxygenase enzyme class shows that Rieske oxygenases co-occurring with SDR enzymes are present throughout the Rieske oxygenase superfamily. In this figure, *green circles* indicate nodes containing at least one Rieske oxygenase that co-occurs with an SDR enzyme. *Pink squares* indicate nodes with at least one structurally characterized Rieske oxygenase, and *blue diamonds* indicate nodes with both at least one co-occurring Rieske oxygenase and a structurally characterized Rieske oxygenase. All clusters that contain a co-occurrence node are within the *green dashed-line square*. Here, co-occurrence is defined as a Rieske oxygenase encoding gene occurring within three genes upstream or downstream of the SDR enzyme in a genome neighborhood diagram. BDO, benzene dioxygenase; BPDO, biphenyl dioxygenase; CARDO, carbazole 1,9a-dioxygenase; CDO, cumene dioxygenase; CntA, carnitine monooxygenase; DdmC, dicamba monooxygenase; GdmA, guaiacol *O-*demethylase; KshA, 3-ketosteroid 9α-hydroxylase; NagGH, salicylate 5-hydroxylase; NBDO, nitrobenzene dioxygenase; NdmA and NdmB, caffeine demethylases; NDO, naphthalene dioxygenase; NTDO, 3-nitrotoluene dioxygenase; OMO, 2-oxoquinoline-8-monooxygenase; PAHDO, PAH-hydroxylating dioxygenase; PDO, phthalate dioxygenase; Stc2, stachydrine demethylase; SxtT and GxtA, saxitoxin biosynthetic enzymes; TDO, toluene dioxygenase; TPADO, terephthalate dioxygenase. In this figure, clusters from the generated SSN with three or fewer nodes are not shown. SDR, short-chain dehydrogenase/reductase.

As a means to identify a library of SDR enzymes that function similarly to TsaC or co-occur with a Rieske oxygenase, a UniRef50 sequence similarity network (SSN) (45, 46, 47, 48, 49) of the SDR enzyme family was constructed (Fig. S55*A*). This SSN contains 138 nodes with structurally characterized SDR enzymes. At an alignment score of 67, TsaC is found in the largest cluster of nodes in the SSN. Of the nodes in the TsaC-containing cluster, 67 contain structurally characterized enzymes, including the node that contains TsaC (Fig. S55*A*). To gain insight into whether additional sequences in this cluster work similarly to TsaC, a phylogenetic tree was constructed to understand the evolutionary relationships among these structurally represented enzymes (Fig. S55*B*). The phylogenetic tree reveals that the structurally characterized enzymes found in the TsaC-containing cluster can generally be classified by clades containing enzymes that operate on similar substrates (Fig. S55*B*). The smallest clade contains TsaC. Within this clade, the only other enzyme for which the substrate has been identified is BDH (28). This result indicates that TsaC is an outlier in this major cluster of the SDR enzyme SSN (Fig. S55*B*). As this structure-based analysis did not highlight a collection of SDR enzymes that work on xenobiotic substrates like TsaC in the major cluster of nodes, a subsequent investigation into the universality of co-occurrence between SDR enzymes and Rieske oxygenases was undertaken. For this venture, genome neighborhood diagrams (45, 46, 47, 48, 49) of nodes in the SDR enzyme SSN were generated and analyzed for all clusters that contain more than ten nodes. This analysis identified 27 nodes that contain SDR enzymes that co-occur with Rieske oxygenases. The corresponding Rieske oxygenase sequences were subsequently mapped onto a second SSN (22) (Fig. 7).

In this Rieske oxygenase SSN, 62 nodes in seven clusters were identified as co-occurrence nodes. A logo diagram analysis of the co-occurring SDR enzymes reveals the presence of common sequence motifs (Fig. S56). Specifically, the TGX_3_GXG (97.3%) and NNAG (86.3%) motifs are found in most of the co-occurring SDR proteins. As these motifs define classical SDR enzymes (23, 24, 29), this analysis reveals that the majority of SDR enzymes found in pathways with a Rieske oxygenase, like TsaC, are classical. The analogous negatively charged residue to Asp36 in TsaC, which is proposed to select for NAD(H) over NADP(H), is found in 95.5% of the sequences in the alignment (Fig. S56). Quite interestingly, this result stands in stark contrast to that described for the majority of classical SDR enzymes, which typically show a preference for employing NADP(H) over NAD(H) in their catalytic cycles (24, 50). With regard to the catalytic tetrad, the Tyr and Lys residues are 100% conserved, the Ser residue is 95% conserved, and the Asn residue is only conserved in 58.9% of the analyzed sequences. These latter Ser and Asn residues are most often substituted by Pro and Asp or His residues, respectively. Finally, the residues that align with the TsaC “gating” residues Thr143 and Gly149 show variability. The Thr143-aligned residues are found primarily as Ile (34%), Asn (34%), or Val (16%) residues, whereas the Gly149-aligned residues are found mainly as Gly (41%), Asn (18%), or Ala (15%) residues. This variation in residue identity is not unexpected. Previous reports (24, 29) indicate that the substrate-binding loop and the loop where the “gating” residues are located typically showcase the most extreme variability with regard to the SDR fold (Figs. S9 and S39).

Returning to the Rieske oxygenase SSN, a large majority of the 62 identified co-occurrence nodes are assembled into a single cluster that contains the structurally characterized Rieske oxygenases BPDO (51), cumene dioxygenase (52), toluene dioxygenase (53), NDO (54), nitrobenzene dioxygenase (55), 3-nitrotoluene dioxygenase (56), and PAH-hydroxylating dioxygenase (57). In contrast to TsaC, which oxidizes a primary alcohol substrate, the SDR enzymes associated with these Rieske oxygenases catalyze rearomatization of their substrates (Fig. 1) (16, 19, 58, 59, 60, 61). Since most co-occurrence nodes are found in this cluster, the SSN suggests that, with our current understanding, an apparent majority of the annotated co-occurring SDR enzymes will facilitate rearomatization chemistry.

Interestingly, the limited set of crystal structures for SDR enzymes that co-occur with a Rieske oxygenase are generally found to work with enzymes in this cluster. Specifically, biphenyl dehydrogenase (BphB) and naphthalene dehydrogenase (NahB) work in pathways with BPDO and NDO, respectively, and catabolize aromatic substrates like TsaC (21, 62). These enzymes, as described previously, like TsaC and BDH, both have the classical SDR motifs and the characteristic Asp for selective binding of NAD(H) over NADP(H). In terms of “gating” residues, both BphB and NahB contain Gly residues that align in space with TsaC “gating” residue Gly146 (Fig. S39). Another SDR enzyme that co-occurs with a Rieske oxygenase is cymene dehydrogenase (CymB). This SDR enzyme catalyzing a remarkably similar reaction to TsaC:CymB transforms 4-isopropylbenzyl alcohol into 4-isopropylbenzaldehyde (16, 63). A sequence alignment of this protein with TsaC again reveals the presence of a small Ala residue in the analogous position to TsaC “gating” residue Gly146 (Fig. S57). As described for TsaC, we hypothesize that a small residue in this “gate” position provides space and confers these SDR-type enzymes the ability to oxidize large aromatic substrates, rather than molecules like the smaller linear *meso*-2,3-butanediol substrate of BDH.

Outside this cluster, eight of the 62 co-occurrence nodes are identifiable in four clusters of the SSN that also have structurally characterized Rieske oxygenases. TsaM is found in a cluster of the SSN that contains dicamba monooxygenase (DdmC, (64)) and guaiacol *O-*demethylase (GdmA) (65). Other co-occurring Rieske oxygenase enzymes are present in the cluster of the SSN that houses stachydrine demethylase (Stc2, (66)) and carnitine monooxygenase (CntA, (67)). One co-occurring Rieske oxygenase node is in the cluster encompassing 3-ketosteroid 9 α-hydroxylase (KshA, (68)). Several co-occurrences are also identifiable in the cluster that contains terephthalate dioxygenase and salicylate 5-hydroxylase (NagGH) (14, 69). The remaining SDR enzymes that co-occur with a Rieske oxygenase occupy three clusters of the SSN, which, to date, have no enzymes that have been structurally characterized. The widespread nature of the co-occurrence nodes and the variability of the identified “gating” residues suggests that other SDR enzymes, like TsaC, will catalyze divergent reactions from the *cis*-dihydrodiol dehydrogenases.

## Discussion

Curiously, despite the vast landscape of chemical diversity that is encountered in nature, only a handful of known chemical entities are used to mediate redox transformations in biological systems. For instance, more than 1000 different redox reactions performed in bacteria require the catalytic power of NAD(H) and NADP(H) (70). The ubiquity of these molecules, which supply a soluble pool of reducing equivalents for biological processes in every kingdom of life (70), presumably stems from their low redox potentials and ability to transfer electrons, both reductively or oxidatively, to substrates, cofactors, and metallocenters (71). In accordance with the widespread biological importance of NAD(H) and NADP(H), and an ever-increasing interest in integrating enzymes in industrial processes, both NAD(H) and NADP(H) are common low-potential redox cofactors that are employed in biocatalysis (10). However, the use of NAD(H) and NADP(H) in such applications does not come without its challenges. In biology, typical metabolic pathways are complicated networks of interconnected reactions, and the concentrations of NAD(H) and NADP(H) are continually managed using NAD^+^ biosynthetic or salvage pathways (70). Understanding the complexities regarding redox balance in cells is important for *in situ* biocatalytic applications. Likewise, elucidating mechanisms to regenerate NAD(H) and NADP(H) at rates that do not limit an enzyme activity of interest for *in vitro* applications are imperative. Previous work in these areas has established that electrochemistry, photochemistry, and other enzyme-mediated chemistry can each be employed to regenerate NAD(H) and NADP(H) (10, 71).

Salient to the work presented here are the NAD(H)- and NADP(H)-dependent SDR and aldehyde dehydrogenase enzymes found in metabolic pathways with Rieske oxygenases (Fig. 1). In addition to their attractive role as redox neutral partner enzymes in catalysis, SDR enzymes are prime candidates on their own for engineering based on the SDR family substrate scope and inherent chiral properties (23). Rieske oxygenases, on the other hand, have received attention for their remarkable ability to initiate the removal of aromatic and polyaromatic hydrocarbons that are low in solubility and high in chemical stability from the environment (72). The natural coupling of these enzyme types in a single pathway, like the other strategies described previously, is attractive for biocatalytic applications that are hindered by the need for cost-prohibitive reducing equivalents such as NAD(P)H and the possible inhibitory effects of accumulating NAD(P)^+^ (10, 71).

To advance our understanding of enzymes that regenerate the NAD(P)^+^ produced by Rieske oxygenase chemistry, in this work, we characterized the SDR enzyme TsaC using X-ray crystallography, enzyme kinetics, light scattering, and differential scanning fluorimetry experiments. From the structure and the presence of residue Asp36, we identified that TsaC is built to favor binding of NAD^+^ over NADP^+^ (Fig. 2) (30). Quite interestingly, whereas this result places TsaC in the minority of SDR proteins, our bioinformatics analyses revealed it is nearly standard among SDR proteins that co-occur with a Rieske oxygenase (Fig. 7). This finding agrees with the primary involvement of NADH, rather than NADPH in catabolic reactions, and also aligns with the noted preference of many experimentally characterized Rieske oxygenase partner reductases for NADH rather than NADPH (11, 14, 67, 73, 74, 75, 76, 77, 78). The NAD^+^ coenzyme requirement of TsaC was verified using activity measurements, and thermal shift data further highlight that combination of NAD^+^ and substrate or NADH alone with TsaC has a stabilizing effect that is not elicited by NADP^+^ (Tables 2 and S4). The enhanced *T*_m_ in the presence of NADH is reminiscent of studies that showed NADH can induce ordering of the substrate-binding loop to a greater extent than NAD^+^ and binds with higher affinity in the SDR enzyme 3α-hydroxysteroid dehydrogenase (44). The consistent tetrameric state of TsaC observed by light scattering experiments further places TsaC in the subgroup of SDR enzymes that appear to remain tetrameric throughout their catalytic cycles or in response to substrate, coenzyme, or product binding (Table S5 and Fig. S48) (79).

The thermal shift data measured for each of the different combinations of TsaC, substrate, and pyridine nucleotides also agree with our inability to model the β6 to α7 loop into the structure of TsaC (Fig. 2; Tables 2 and S4). Testing of our substrate panel reveals three sets of different substrate–enzyme interactions in the measured *T*_m_ data. One class of compounds do not support TsaC activity and correspondingly do not show a measurable increase in the *T*_m_ of TsaC. In contrast, most substrates on which TsaC is active show elevated responses in *T*_m_, which is consistent with a model in which substrate binding induces a conformational shift that shields the active site and sequesters hydride transfer away from bulk solvent (23, 32). In line with this model, the last set of compounds support TsaC activity, without a corresponding change in *T*_m_. Substrates in this category include 4-formylbenzenesulfonate and 4-formylbenzoate, which we further show TsaC can transform into 4-sulfobenzoate and 1,4-benzenedicarboxylate, respectively (Fig. 3). Yet, we also indicate that this chemistry appears to rely on solution chemistry to first produce aldehyde hydrates, a feat that would be possible in an open solvent accessible active site. The relevance of such a water-mediated mechanism to *in vivo* Tsa pathway chemistry, at this point, is unclear. However, given that TsaD is catalytically superior in forming 4-sulfobenzoate and 1,4-benzenedicarboxylate, relative to TsaC, and does not rely on solution chemistry, it is likely the TsaC chemistry can be thought of as an off-pathway *in vitro* side reaction.

In accordance with this proposal, further analysis of TsaC with both products of its native reaction, 4-formylbenzoate and NADH, also supports other data collected in this work that suggest TsaC, like other SDR enzymes (31), follows the ordered “bi–bi” mechanism. Specifically, our data reveal that addition of 4-formylbenzoate to a mixture of TsaC and NADH negates any stabilizing effect of NADH on the protein. This result suggests that the reported native oxidized product promotes dissociation of the coenzyme from TsaC (Tables 2 and S4; Figs. S9 and S47). This last class of substrates also includes 5-hydroxypenanoate and 4-chlorobenzyl alcohol. Considering the lack of conservation of the loop region displayed in our bioinformatic analysis, and its known role in dictating the substrate specificity of BphB, NahB, and BDH, we posit that these molecules bind in the TsaC active site but are unable to trigger the typical loop-closing conformational change (Fig. S39) (21, 28, 62). A similar uncoupling of catalysis from thermal stability was also measured for the double variant in the presence of *meso*-2,3-butanediol. This variant shows high levels of activity on *meso*-2,3-butanediol because of the rational changes, informed by the crystal structure, that we made in the TsaC active site (Fig. 5). These changes were made at the identified “gate” residues, which we anticipated would play a key role in controlling the size of accepted substrate. Indeed changing these residues into their BDH counterparts (T143Q/G149N) is hypothesized to decrease the size of the TsaC active site and, at the same time, allow *meso*-2,3-butanediol to form interactions that support catalysis. Therefore, we posit that we can think of the residues in these “gating” positions as fingerprints that are correlated with the identity and scope of substrates accepted by SDR proteins. Future engineering efforts to analyze how cooperative mutations in both the active site and on the loop coordinate to dictate specificity and catalytic activity of SDR enzymes are paramount.

As co-occurrence pathways can allow microorganisms to recoup reducing equivalents and mitigate the energetic cost of Rieske oxygenase chemistry, the final experiments in this work were focused on demonstrating that the NADH recycled by TsaC and TsaD could support the Rieske oxygenase chemistry of TsaM (Fig. 6). Notably, our kinetic analysis shows that even though TsaC mediates conversion of 4-formylbenzenesulfonate and 4-formylbenzoate to 4-sulfobenzoate and 1,4-benzenedicarboxylate, respectively, TsaD has a higher catalytic efficiency for this chemistry. Furthermore, the overall measured kinetic parameters of TsaC on its native substrates, 4-(hydroxymethyl)benzenesulfonate and 4-(hydroxymethyl)benzoate, and TsaD on its native substrates, 4-formylbenzenesulfonate and 4-formylbenzoate, are comparable. Together, the data suggest that, *in vivo*, the Rieske oxygenase chemistry is the flux-limiting enzyme in the Tsa pathway. Combined with our rational engineering of TsaC, and our previous work establishing design principles for engineering TsaM (22), we posit these data are strongly supportive of the notion that SDR enzymes that co-occur with Rieske oxygenases are promising for exploring and engineering self-sustained systems that can be used for *in vitro* applications or *in vivo* applications.

## Experimental procedures

### Site-directed mutagenesis

For the variants used in this work, mutated plasmids were either produced using an Agilent QuikChange Lightning Site-Directed Mutagenesis Kit or a New England Biolabs Q5 Site-Directed Mutagenesis Kit. Regardless of the kit used, primers were synthesized by IDT, and the sequences of these primers are listed in Table S2. All PCR was performed using a Bio-Rad C1000 Thermocycler. To verify the incorporation of desired mutations, Sanger sequencing (Genewiz) was employed. For the Agilent QuikChange Lightning Site-Directed Mutagenesis Kit, 100 ng of dsDNA template (pET-28a(+)-tobacco etch virus (TEV)-*tsaC*) and 125 ng of each oligonucleotide primer were used in the reaction mixture. Following PCR, reaction mixtures were digested with 1 μl of DpnI restriction enzyme for approximately 15 min at 37 °C. The digested mixtures were transformed into XL10-Blue ultracompetent cells and plated on lysogeny broth (LB)—Miller formulation agar with 50 μg/ml kanamycin antibiotic. For the New England Biolabs Q5 Site-Directed Mutagenesis Kit, 15 ng of dsDNA template (pET-28a(+)-TEV-*tsaC* or pMCSG9-*tsaD*) and 10 μM of each oligonucleotide primer were used in the reaction mixture. Following PCR, amplified products were digested and ligated using a kit-supplied kinase–ligase–digest mixture for 5 min at room temperature. The digested mixtures were transformed into NEB5α-competent cells and plated on LB agar with 50 μg/ml kanamycin (*tsaC*) or 100 μg/ml ampicillin (*tsaD*).

### Protein expression of wild-type TsaC and TsaC variants

A codon-optimized version of the gene encoding an N-terminally His_6_-tagged TsaC was synthesized and cloned into a pET-28a(+)-TEV plasmid (Genscript). Using the standard heat shock protocol, the plasmid was then transformed into C41(DE3) chemically competent *E. coli* cells (Novagen). The transformed cells were plated onto LB agar with kanamycin (50 μg/ml) and incubated overnight at 37 °C. From the overnight growth, a single colony containing the pET-28a(+)-TEV-*tsaC* plasmid was used to inoculate 8 ml of LB media with kanamycin (50 μg/ml). This starter culture was incubated with shaking (200 rpm) overnight at 37 °C. The overnight culture was then inoculated into 1 l of LB media with kanamycin (50 μg/ml). The culture was shaken at 200 rpm and incubated at 37 °C. The absorbance of the culture was monitored by measuring the absorbance at 600 nm until a value between 0.6 and 0.8 was reached. After reaching the target cell density, the cell cultures were removed from the shakers and allowed to cool to 20 °C. Once cooled, the cultures were induced with 0.5 mM IPTG, and the cultures were shaken at 160 rpm and incubated at 25 °C overnight. The overnight-incubated cultures were harvested by centrifugation at 5000 rpm for 20 min at 4 °C, producing a cell pellet after decanting the supernatant. The typical pellet wet mass for a 1 l culture of wild-type TsaC was 6 g. The TsaC variants produced a typical pellet wet mass of 4 g per 1 l culture.

### Protein purification of wild-type TsaC and TsaC variants

Typically, two collected cell pellets containing the His_6_-tagged TsaC were resuspended in 60 ml of lysis buffer (50 mM Hepes [pH 8.0] and 200 mM NaCl). The resuspended cells were lysed *via* sonication on ice using a Fisherbrand Model 120 Sonic Dismembrator. A 10 min program at 30% amplitude for cell lysis was cycled between steps of being on for 5 s and off for 10 s. The lysed solution was then centrifuged for 45 min at 12,000 rpm at 4 °C. The clarified solution was transferred to a new container and applied to a pre-equilibrated HisTrap HP column (Cytiva). The column was washed with five column volumes of buffer A (50 mM Hepes [pH 8.0], 200 mM NaCl, and 10 mM imidazole), followed by five column volumes of 10% buffer B (50 mM Hepes [pH 8.0], 200 mM NaCl, and 200 mM imidazole), and five column volumes of 20% buffer B. The tagged protein was then eluted from the HisTrap column with eight column volumes of 100% buffer B. The protein in the eluted fractions was identified by SDS-PAGE and concentrated to a volume of 1 ml using a 10 kDa molecular weight cutoff (MWCO) centrifugal filter (Amicon). The concentrated fractions were loaded onto a HiPrep 16/60 Sephacryl S200-HR (Cytiva) gel filtration column that was pre-equilibrated with buffer C (50 mM Hepes [pH 8.0], 200 mM NaCl, and 5% glycerol). From this column, the eluted tetrameric TsaC protein was collected and concentrated to 200 μM. The concentrated protein was split into 100 μl aliquots and flash frozen using liquid nitrogen prior to storage at −80 °C.

### Protein expression conditions of wild-type TsaD and the C286A TsaD variant

A codon-optimized version of the gene encoding an N-terminally maltose-binding protein (MBP)-His_6_-tagged TsaD was synthesized and cloned into a pMCSG9 plasmid (Genscript). The protein expression protocol followed the TsaC expression protocol described previously, except that ampicillin (100 μg/ml), rather than kanamycin, was used every time that antibiotics were introduced. The typical pellet wet mass for a 1 l culture of wild-type TsaD was 4 g. The TsaD variants produced a typical pellet wet mass of 1.5 g per 1 l culture.

### Protein purification of wild-type TsaD and the C286A TsaD variant

Typically, two collected cell pellets containing the MBP-His_6_-tagged TsaD were resuspended in 60 ml of lysis buffer (50 mM Hepes [pH 8.0] and 200 mM NaCl). The resuspended cells were lysed and centrifuged as described for TsaC. The clarified solution was transferred to a new container. The supernatant was applied to a pre-equilibrated MBPTrap column (Cytiva). The column was washed with eight column volumes of buffer A (50 mM Hepes [pH 8.0], 200 mM NaCl, and 1 mM DTT) and then eluted with eight column volumes of 100% buffer B (50 mM Hepes [pH 8.0], 200 mM NaCl, 1 mM DTT, and 10 mM maltose). The eluted fractions were identified by SDS-PAGE, and the fractions containing the target-tagged TsaD were pooled together. The tagged protein was buffer exchanged into 50 mM Hepes (pH 8.0), 200 mM NaCl, 1 mM DTT, and 5% glycerol using SnakeSkin dialysis tubing (Thermo Scientific) with a 3.5 kDa MWCO, and the tag was simultaneously cleaved by incubation with approximately 3 mg of TEV overnight. The dialyzed and digested sample was applied to a pre-equilibrated HisTrap HP column in which the flow-through was collected and analyzed by SDS-PAGE to identify fractions containing the untagged TsaD. The untagged TsaD protein was concentrated using a 50 kDa MWCO centrifugal filter unit. Approximately 1 ml of concentrated TsaD was loaded onto a HiPrep 16/60 Sephacryl S200-HR gel filtration column that was pre-equilibrated with buffer C (50 mM Hepes [pH 8.0], 200 mM NaCl, and 5% glycerol). The eluted dimeric TsaD protein from this column was collected and concentrated to 100 μM. The concentrated protein was split into 50 μl aliquots and flash frozen using liquid nitrogen prior to storage at −80 °C.

### Protein expression and purification conditions for wild-type TsaM

To produce wild-type TsaM protein samples, a similar protocol to that previously described was followed (22). In short, single colonies of C41(DE3) *E. coli* cells containing the pET-28a(+)-TEV-*tsaM* plasmid (Genscript) were grown as described for TsaC. At induction, 0.1 mM IPTG, 0.2 mg/ml ferric ammonium citrate, and 0.4 mg/ml ferrous ammonium sulfate hexahydrate were added to the 1 l cultures. The large-scale cultures were incubated for an additional 18 h at 18 °C with shaking at 160 rpm. The average pellet for a 1 l culture was 6 g of wet cell mass. The purification of wild-type TsaM protein samples adhered to a similar protocol as previously described (22). Briefly, the collected pellets from two 1 l cultures were resuspended in 60 ml of lysis buffer (50 mM Tris–HCl [pH 7.2] and 250 mM NaCl). The resuspended pellets were lysed, clarified, and purified as described for TsaC. The concentrated (118 μM or 10 mg/ml) trimeric protein fractions from gel filtration chromatography were aliquoted into 200 μl fractions and flash frozen using liquid nitrogen and stored at −80 °C.

### Protein expression and purification conditions for VanB

The expression and purification methods for VanB were previously described and followed here (80). Briefly, single colonies of BL21(DE3) *E. coli* cells containing the pMCSG7-TEV-*vanB* plasmid (Genscript) were expressed as described for TsaD, except cultures were shaken for 18 h at 18 °C following induction. The cells containing the target protein were harvested by centrifugation.

For purification of VanB, the cell pellets from two 1 l growths were resuspended in 60 ml of lysis buffer (50 mM Tris–HCl [pH 7.2], 250 mM NaCl, 10 mM imidazole, and 100 μM flavin adenine dinucleotide). The resuspended cells were lysed *via* sonication and purified following the same method described for TsaM. The eluted fractions of VanB were collected, concentrated to 200 μM, aliquoted into 200 μl fractions, and flash frozen using liquid nitrogen prior to storage at −80 °C. The incorporation of flavin adenine dinucleotide was assessed by using the characteristic UV–Vis absorbance maximum at 450 nm (extinction coefficient of 11,300 M^−1^ cm^−1^) (81).

### CD experiments

CD experiments were performed as previously described (82). Stock solutions containing 200 μM of wild-type TsaC and its variants were diluted to 2.5 μM using 2.5 mM Hepes (pH 8.0) and 10 mM NaCl. TsaD and the C286A variant were treated similarly. A 350 μl sample of diluted protein was used for the measurements. The data shown are an average of four spectra of each sample. Experiments were performed using a Jasco J-1500 CD spectrometer.

### Iron quantification for isolated TsaM

The iron content of isolated TsaM was assessed by ferrozine analysis following the published protocol (83). The number of iron ions incorporated for isolated TsaM was determined to be 2.6 per monomer.

### Thermal shift assays

To measure the thermal stability of TsaC and TsaD, thermal shift assays were conducted using a QuantStudio5 real-time PCR following previously described protocols (22, 84). Enzyme samples (10 μM unless otherwise noted) were combined with an eightfold dilution of Protein Thermal Shift Dye in thermal shift buffer in a 96-well plate. Different concentrations of NAD^+^, substrate, or product compounds were added to a final volume of 20 μl. Mixtures were then subject to melting over a temperature range of 25 to 99 °C at a ramp rate of 0.05 °C/s. The *T*_m_ was determined by fluorescence curves at 570 nm using Applied Biosystems Protein Thermal Shift Software, version 1.4. Each sample was measured in triplicate.

### Light scattering

To assess changes in the oligomeric state of TsaC, in-line SEC–MALS and DLS experiments were conducted. SEC–MALS experiments were conducted using an Agilent 1260 Infinity II HPLC equipped with a 4 °C chilled multisampler and an AdvanceBio 300A 2.7 μm 4.6 × 300 mm (Agilent) SEC column. The SEC–MALS column was pre-equilibrated in buffer consistent with the individual experimental condition (50 mM Hepes [pH = 8.2], 200 mM NaCl, 5% [v/v] glycerol, and either NAD^+^ or NADH). The experiment was initiated with a 15 μl injection of 90 μM protein, and the column was run at room temperature and 0.25 ml/min. SEC–MALS data were collected as the protein migrated out of the SEC column on a Wyatt Neon DAWN ambient and analyzed using Astra Software, version 8.2. Concentrations were measured using both a 1260 Infinity II multiwavelength detector monitoring absorbance at 280 nm and the OptiLab Refractive Index Detector (Wyatt). DLS experiments were conducted with 5 μM to 1 mM enzyme, 1 mM to 2 mM coenzyme, and 1 mM to 2 mM substrate–product as indicated. DLS data were collected at 25 °C using a Wyatt microvolume disposable cuvette on a Wyatt DynaPro NanoStar II, with three data segments collected per sample with a 1-min incubation between each segment. DLS data were analyzed using Dynamics Software, version 8.2 (Waters, Wyatt Technology).

### Protein crystallization

Crystallization of TsaC was accomplished by combining 10 to 20 mg/ml of protein containing 2 mM NAD^+^ and a crystallization mother solution of 0.2 M KCl, 20% polyethylene glycol 3350, and 10% glycerol, equilibrated against the mother solution using vapor diffusion with hanging drops. The trays were incubated at 293 K, and, when formed, crystals were harvested at room temperature, soaked in a cryoprotectant of 20% PEG 3350, and frozen in liquid nitrogen. Despite much effort, crystals providing structures with convincing electron density for bound NAD^+^ or substrate were not produced.

### X-ray diffraction data collection, processing, and structure determination

X-ray diffraction data were collected at the Life Sciences Collaborative Access Team beamline 21-ID-F at the Advanced Photon Source, Argonne National Laboratory. The dataset was collected at a temperature of 100 K and a wavelength of 0.97872 Å. The diffraction data were indexed, integrated, and scaled using the X-ray Detector Software (XDS) program package (85). Statistics from XDS and Pointless were used to determine the identity of the space group to be P 3_2_ 2 1 (space group 154) (86). Further work toward structural determination was conducted using the Phenix suite, beginning with Xtriage (87). Molecular replacement was performed with Phaser, using BDH (PDB ID: 1GEG (28)) as a search model. This structure was chosen as it was identified as the structure in the PDB with the most similar primary sequence to TsaC using the BLAST (88). This initial solution contained two protein chains per asymmetric unit, with a solvent content of approximately 41%. Following simulated annealing, and reservation of 5% of the reflections for an *R*_free_ test dataset, refinement of the structure was performed using both Coot and the Refine program within Phenix (89). The quality of the structure at each step was established using MolProbity (90). The model of TsaC contains all protein residues except the N-terminal His_6_ tag and TEV protease cleavage site (residues -20 to -1), the native initial methionine residue (residue 1), residues 188 to 220, and the four C-terminal residues 249 to 252 in chain A. Chain B is missing all the same residues plus residues 145 to 150 and residue 187.

The final structure was refined to a resolution of 2.18 Å, and the final processing and refinement statistics are reported in Table S1. Figures of the TsaC structure were made using PyMOL (The PyMOL Molecular Graphics System, version 2.5.4; Schrödinger, LLC). Structural biology applications used in this project were compiled and configured by SBGrid (91). Buried surface area analysis was calculated using PyMOL and the PISA software (92). Substrate geometry parameters used to model 4-(hydroxymethyl)benzoate and 4-(hydroxymethyl)benzenesulfonate into the structure of BDH were calculated using the eLBOW program within the Phenix software suite (93). The protein coordinates of TsaC have been deposited in the PDB with the accession code 8SY8.

### Bioinformatics

An SSN of the SDR family (InterPro (94) IPR002347) was generated using the Enzyme Function Initiative (45, 46, 47, 48, 49) Enzyme Similarity Tool. UniRef50 clusters, which contain sequences that share 50% identity and greater than 80% overlap with the longest sequence in the cluster, were used. The SSN was generated with an *E* value of 5 and separated to an alignment score threshold of 67, as at higher alignment score threshold values the node containing TsaC separated from the main node cluster. A UniRef50 SSN of the Rieske oxygenase family was generated as previously described (22); briefly, an initial SSN of InterPro families with Rieske-type [2Fe–2S] clusters was generated and then curated to exclude non-Rieske oxygenase nodes when separated at alignment score threshold of 60. Cytoscape 3.8.2 (95) was used for visualization of all SSNs. Phylogenetic tree construction was conducted with the MEGA11 software package (96) using the maximum likelihood method that employs the Jones–Taylor–Thorton model (97) to generate a most likely phylogenetic tree after a heuristic search of initial trees. The Enzyme Function Initiative (45, 46, 47, 48, 49) Genome Neighborhood Tool was used for the analysis of the genomic context of SDR enzymes. Mutiple sequence alignments were performed using the Clustal Omega webserver (98), and logo diagrams were created using the WebLogo 3 webserver (99).

### Substrate and product standards used in enzymatic reactions

All compounds used were commercially purchased and are listed in Table S3. The purity of these compounds is over 95%.

### Stock preparation for enzymatic assays

All stock solutions were prepared in dimethyl sulfoxide (analytical grade) to a final concentration of 0.1 M, with the exception of 4-aminobenzaldehyde, *meso*-2,3-butanediol, and 3-hydroxy-2-butanone, which were prepared in methanol. Stock solutions of NAD^+^ and NADH were prepared in ultrapure water to a final concentration of 10 mM and stored at −20 °C. The stock solution of ferrous ammonium sulfate hexahydrate was prepared and stored at 5 mM concentration at −20 °C. To ensure the consistency of enzymatic assays, the enzyme aliquots were discarded after one freeze–thaw cycle. The ferrous ammonium sulfate hexahydrate, NADH, and NAD^+^ aliquots were discarded after no more than five freeze–thaw cycles.

### TTN determination of TsaC- and TsaD-catalyzed reactions

Wild-type TsaC and TsaC variants were prepared to a final concentration of 400 μM, and wild-type TsaD and TsaD variants were prepared to a final concentration of 200 μM. Reactions (50 μl) consisting of 5 μM enzyme, 2 mM substrate, 2 mM NAD^+^, and 20 mM Tris–HCl (pH 7.0) buffer were mixed and incubated at 30 °C for 3 h. Reactions were quenched with the addition of 100 μl acetonitrile containing our mass spectrometry (MS) internal standard, acetaminophen. The quenched reactions were centrifuged at 17,000*g* for 15 min. The supernatant (50 μl) was transferred to sample vials and run on the LC–MS. For most reactions, the TTN was determined by comparison to standard curves of the respective reaction starting material. For TsaC reactions with benzyl alcohol, 4-isopropylbenzyl alcohol, or 4-chlorobenzyl alcohol and all TsaD reactions, comparison to standard curves of respective reaction products was used instead. The substrate and product standard curves were plotted using the following relationship:

$$\text{Ratio}=\frac{\text{Peak}\;\text{area}\;\text{of}\;\text{product}\;\text{or}\;\text{peak}\;\text{area}\;\text{of}\;\text{substrate}}{\text{Peak}\;\text{area}\;\text{of}\;\text{internal}\;\text{standard}}$$

The ratio obtained from the enzymatic reactions was then used to solve for the concentration of substrate consumed or product generated. To obtain the amount of product generated in the TsaC reactions when the substrate standard curve was used, the value calculated from the standard curves was subtracted from the initial quantity of starting material (2 mM). In these cases, TTNs were calculated using the following equation:

$$\text{TTNs}=\frac{\left(\text{Initial}\;\text{quantity}\;\text{of}\;\text{subtrate}\;\left(2\;\text{mM}\right)-\text{substrate}\;\text{amount}\;\text{left}\;\left(\text{mM}\right)\right)x\;1000}{\text{Total}\;\text{concentration}\;\text{of}\;\text{enzyme}\;\text{used}\;\left(5\;\mu M\right)}$$

To obtain TTNs of products generated in TsaD and TsaC reactions when using benzyl alcohol, 4-isopropylbenzyl alcohol, or 4-chlorobenzyl alcohol as a substrate, the generated product concentration was obtained using the ratio calculated from the enzymatic assay. TTNs were calculated using the following equation:

$$\text{TTNs}=\frac{\text{Total}\;\text{concentration}\;\text{of}\;\text{generated}\;\text{product}\;\left(\mu M\right)}{\text{Total}\;\text{concentration}\;\text{of}\;\text{enzyme}\;\text{used}\;\left(5\;\mu M\right)}$$

All product standard curves and enzymatic reactions were conducted and analyzed in triplicate. Substrate standard curves used in the work are shown in Fig. S59. Product standard curves used in this work are shown in Fig. S58.

### Kinetic analysis

The investigation of the steady-state kinetics of TsaC and TsaD started with assessment of the linear range of product formation. To begin, 50 μl enzymatic reactions were prepared consisting of 1 mM substrate, 2 mM NAD^+^, and different concentrations of TsaC or TsaD (2, 5, and 10 μM). Varying enzyme concentrations were used to ensure the formed product signal is in the detectable range of LC–MS. The enzymatic reaction was incubated at 30 °C and quenched at 2, 5, 10, and 40 min time points by the addition of 100 μl acetonitrile containing the MS standard, acetaminophen.

To determine the steady-state kinetic parameters for the TsaC- and TsaD-catalyzed reactions, reactions were conducted on a 50 μl scale with 2 mM NAD^+^ and a substrate range from 1 to 600 μM. The reactions were initiated by the addition of enzyme. Reactions were quenched after the time determined from the previous linear range experiments by the addition of 100 μl acetonitrile with our MS internal standard. The reactions were centrifuged at 17,000*g* for 15 min. The supernatant (50 μl) was transferred to sample vials and further analyzed by LC–MS. For TsaC-catalyzed reactions, the amount of product generated was calculated by substrate consumption using the substrate standard curve, with the exception of benzyl alcohol, 4-isopropylbenzyl alcohol, and 4-chlorobenzyl alcohol (Fig. S58). For TsaD-catalyzed reactions and TsaC-catalyzed reactions on benzyl alcohol, 4-isopropylbenzyl alcohol, and 4-chlorobenzyl alcohol, the product amount generated was calculated using the product standard curve (Fig. S58). The data were plotted and fit to the Michaelis–Menten equation using GraphPad Prism nine (GraphPad Software, Inc). All reactions were performed in triplicate.

### ^18^O-labeled water incorporation experiments

To probe the mechanism of TsaC with 4-formyl benzenesulfonate and 4-formylbenzoate substrates, TsaC was prepared to a final concentration of 400 μM. A stock solution of 10 mM NAD^+^ was prepared in ^18^O-water (^18^O, 97%). Reactions (50 μl) consisting of 5 μM enzyme, 2 mM substrate, 2 mM NAD^+^, and 20 mM Tris–HCl (pH 7.0) buffer prepared in H_2_^18^O were mixed and incubated at 30 °C for 3 h. The reaction mixtures were quenched and analyzed as described previously.

### TTN determination of TsaM-catalyzed reactions

TsaM prepared following the protocol described previously was concentrated to a stock solution of 250 μM and stored at −80 °C. Enzymatic reactions (50 μl) consisting of 2 mM substrate, 2 mM NADH, 500 μM ferrous ammonium sulfate hexahydrate, 2.5 μM TsaM, and 10 μM VanB were mixed and incubated at 30 °C for 3 h. The reaction was quenched and analyzed as described previously.

The TTNs of TsaM-catalyzed reactions were calculated based on the amount of the product generated by using the product standard curve in Fig. S58. The calculation of TTNs follows the equation as below:

$$\text{TTNs}=\frac{\text{Total}\;\text{concentration}\;\text{of}\;\text{generated}\;\text{product}\;\left(\mu M\right)}{\text{Total}\;\text{concentration}\;\text{of}\;\text{enzyme}\;\text{used}\;\left(2.5\;\mu M\right)}$$

Enzymatic reactions were prepared and analyzed in triplicate.

### Activity assay of Tsa pathway reactions

To assess the ability of TsaM, VanB, TsaC, and TsaD to convert the native substrates, 4-methylbenzenesulfonate and 4-methylbenzoate, into product, 50 μl reactions consisting of 1 mM substrate, 1 mM NADH, 200 μM ferrous ammonium sulfate hexahydrate, 5 μM TsaM, 20 μM VanB, 5 μM TsaC, and 5 μM TsaD were made. The reaction was initiated by the addition of NADH. The enzymatic reaction mixture was incubated at 30 °C for 3 h and then quenched by the addition of 100 μl acetonitrile containing internal standard. The quenched reaction was centrifuged at 17,000*g* for 15 min. For analysis by LC–MS, 50 μl of the supernatant was transferred into the sample vial.

### Spectroscopic assay of Tsa pathway–catalyzed reactions

To evaluate whether TsaC and TsaD can recycle the NADH produced in the TsaM-catalyzed reaction, the amount of NADH consumed and regenerated was measured using a BioTek Epoch2 microplate reader by monitoring the absorbance at 340 nm. To begin, 50 μl enzymatic reactions consisting of 1 mM substrate, 5 μM TsaM, 20 μM VanB, 200 μM ferrous ammonium sulfate hexahydrate, and 1 mM NADH were prepared. The 1 mM NADH concentration was chosen because it is in the proper detection range of the plate reader. The reaction was initiated by addition of NADH. After NADH addition, the absorbance at 340 nm was measured for every 10 s for 30 min. After 30 min, 5 μM TsaC, 5 μM TsaD, or 5 μM TsaC and 5 μM TsaD were added to the enzymatic reaction. This reaction mixture was monitored at 340 nm every 10 s for 4000 s. Of note, there was approximately a 100 s delay before data collection after the initial addition of NADH and another 100 s of delay after the addition of TsaC, TsaD, or TsaC and TsaD.

### Synthesis using Corey–Suggs reagent and Jones reagent

Because of the difficulty of procuring 5-oxopentanoate and glutarate, these product standards were synthesized using the Corey–Suggs reagent pyridinium chlorochromate (PCC) and Jones reagent chromium trioxide (CrO_3_). To synthesize 5-oxopentanoate, a 100 mM stock solution of PCC was prepared in dichloromethane. A 150 μl PCC solution was combined with 100 μl of 100 mM 5-hydroxypentanoate and 200 μl of acetone. The reaction mixture was incubated at room temperature for 2 h with constant stirring. For the synthesis of glutarate, CrO_3_ was prepared in concentrated sulfuric acid to a final concentration of 100 mM. A 100 μl CrO_3_ solution was then mixed with 100 μl of 100 mM 5-hydroxypentanoate, 200 μl acetone, and 200 μl water, and incubated at room temperature for 3 h with constant stirring. Then, 50 μl of each reaction mixture was diluted with 250 μl acetonitrile containing acetaminophen as an internal standard. The mixture was further analyzed using LC–MS methods described later.

### LC–MS analysis of enzymatic reactions

LC–MS analysis was performed on an Agilent G6545A quadrupole-time of flight mass spectrometer equipped with a dual AJS ESI source and an Agilent 1290 Infinity series diode array detector, autosampler, and binary pump. For the majority of substrates and products, the LC–MS solvents A and B were 5% acetonitrile, 95% water, and 20 mM ammonium acetate (pH 5.5) and 95% acetonitrile, 5% water, and 20 mM ammonium acetate (pH 5.5), respectively. The instrument was run in negative ion mode. For 4-aminobenzyl alcohol, benzyl alcohol, 4-isopropylbenzyl alcohol, 4-chlorobenzyl alcohol, and their corresponding products, the LC–MS solvents A and B were 5% acetonitrile, 95% water, and 0.1% formic acid, and 95% acetonitrile, 5% water, and 0.1% formic acid, respectively. In these cases, the data collection was run in positive ion mode.

For LC to separate each substrate from its product, an Agilent ZORBAX Rapid Resolution HT 3.5 μm, 4.6 × 75 mm SB-CN liquid column was used for most substrates and products, with the exception of benzyl alcohol, 4-isopropylbenzyl alcohol, and 4-chlorobenzyl alcohol. The chromatographic method used for these substrates was as follows: 10% solvent B from 0 to 1 min (to waste) and then a gradient that ran from 10% to 95% solvent B from 1.0 to 4.0 min, which was followed by a 1 to 1.5 min isocratic flow at 95% solvent B. The column is re-equilibrated with 1 min 10% solvent B, at a 0.4 ml/min flow rate.

For the separation of benzyl alcohol, 4-isopropylbenzyl alcohol, and 4-chlorobenzyl alcohol from their products, an Agilent ZORBAX Rapid Resolution HT 1.8 μM, 2.1 × 50 mm SB-Aq liquid column was used. The method used with this column was as follows: 10% solvent B from 0 to 1 min (to waste), a gradient that ran from 10% to 60% solvent B from 1 to 2 min, and a second gradient from 60% to 95% solvent B from 2 to 3 min, followed by a 1.0 min isocratic flow of 95% solvent B (to MS). This step was followed by re-equilibration with 10% solvent B for another 1 min (to waste), at a flow rate of 0.4 ml/min. For this set of compounds, because of the difficulty associated with the ionization of benzyl alcohol, 4-isopropylbenzyl alcohol, and 4-chlorobenzyl alcohol, the diode array detector was used to detect the presence of substrate at 260 nm.

### Enzymatic assay for GC–MS reactions

Wild-type TsaC and TsaC variants were purified as described above. A 100 μl reaction containing 10 μM TsaC or a TsaC variant, 5 mM substrate (*meso*-2,3-butanediol), and 5 mM NAD^+^ was combined and incubated at 30 °C for 2 h. Reactions were then quenched using 100 μl acetonitrile and centrifuged at 17,000*g* for 15 min. Following centrifugation, a 100 μl aliquot of the supernatant was diluted with 80 μl acetonitrile and 20 μl acetonitrile containing our GC–MS internal standard, xylene (34 mM). This 200 μl mixture was then loaded onto a silica plug (0.4 cm radius × 1.0 cm) in a glass pipette to remove any salt from the enzymatic reaction. The silica was rinsed with 150 μl portions of acetonitrile two consecutive times, and the combined eluent was analyzed on GC–MS as described below. All assays were run in triplicate.

### GC–MS analysis of enzymatic reactions

GC–MS analysis used a Thermo Scientific ISQ 7000 single quadrupole mass spectrometer equipped with Thermo Scientific Trace 1310 gas chromatograph and Thermo Scientific GV-1MS 0.32 mm ID × 30 m by 0.50 μM film column. The GC–MS analysis methods were as follows: He (99.9999%) was used as the carrier gas at flow rate of 4.0 ml/min. The inlet temperature was maintained at 225 °C at a split flow of 25 ml/min. The initial oven temperature was set to 40 °C and was held for 0.2 min. The oven temperature was then raised to 200 °C at a rate of 25 °C/min from 0.2 to 6.6 min. A brief hold at 200 °C (0.4 min) completed a total run time of 7 min. The MS transfer line temperature was set to 250 °C, and the ion source temperature was 310 °C. The setting of the repeller voltage was 3.0 V with electron lens voltage as 5.0 V and electron energy as 71.0 V.

## Data availability

Protein coordinates and structure factors have been submitted to the PDB under accession code 8SY8.

## Supporting information

This article contains supporting information (7, 8, 9, 21, 28, 29, 35, 40, 62, 79, 92, 100, 101, 102, 103, 104, 105, 106, 107).

## Conflict of interest

The authors declare that they have no conflicts of interest with the contents of this article.
