# Supplementary material for: The NADH recycling enzymes TsaC and TsaD regenerate reducing equivalents for Rieske oxygenase chemistry
Source: J Biol Chem. 2023 Sep 9;299(10):105222. doi: 10.1016/j.jbc.2023.105222 (PMC10579966; doi:10.1016/j.jbc.2023.105222)
Supplement: Supporting information [file mmc1.pdf]

Supporting Information for:

The NADH recycling enzymes TsaC and TsaD regenerate reducing equivalents for Rieske oxygenase chemistry

Jiayi Tian[§1], David G. Boggs[§1], Patrick H. Donnan[1], Gage T. Barroso[1], Alejandro Arcadio Garcia[1], Daniel P. Dowling[2], Joshua A. Buss[1], and Jennifer Bridwell-Rabb[1]*

[1]Department of Chemistry, University of Michigan, Ann Arbor, Michigan, 48109
[2]Department of Chemistry, University of Massachusetts Boston, Boston, MA, 02125
*To whom correspondence should be addressed: jebridwe@umich.edu
[§]These authors contributed equally to this work

Table of Contents:

I. Supporting Information Tables S3-S5

Table S1. Data processing and refinement statistics for X-ray crystallography………… S3
Table S2. Primers for performing site-directed mutagenesis on TsaC and TsaD……… S3
Table S3. Substrates used in this work……………………………………………………S4
Table S4. Melting temperatures of wild-type TsaC determined using thermal shift assays..S6
Table S5. Size exclusion chromatography multiangle light scattering (SEC-MALS)………..S6

II. Supporting Information Figures S7-S68

Figure S1. TsaC can be recombinantly expressed and purified…………………..……..S7
Figure S2. Sequence alignment of TsaC and *meso*-2,3-butanediol dehydrogenase……… S8
Figure S3. TsaC behaves as a tetramer *in crystallo*………………………………………… S9
Figure S4. A conserved SDR-type NAD(H)/NADP(H) binding pocket………………………S10
Figure S5. The catalytic tetrad of residues is modeled in an inactive orientation………….S11
Figure S6. Active and inactive catalytic tetrad orientations……………………………….. S12
Figure S7. NAD^+ binding in TsaC requires a conformational shift…………………………...S13
Figure S8. TsaC adopts a tetrameric oligomeric state in the crystal structure.……………S14
Figure S9. Structural divergence in the substrate binding loop in SDR enzymes.…………S15
Figure S10. Topology diagrams identify conserved residues in TsaC………………………S16
Figure S11. An optimum pH value for measuring the activity of TsaC was determined…..S17
Figure S12. TsaC performs chemistry on 4-(hydroxymethyl)benzenesulfonate substrate..S18
Figure S13. TsaC performs chemistry on 4-(hydroxymethyl)benzoate substrate.…………S19
Figure S14. $NADP^+$ does not support the activity of TsaC……………………………………S20
Figure S15. A Y155F variant of TsaC is inactive……………………………………………S21
Figure S16. TsaC-catalyzed reactions was investigated in ^{18}O-labeled water.……………..S22
Figure S17. TsaC is more active with alcohol substrates.…………………………………… S23
Figure S18. Kinetics of TsaC with a 4-(hydroxymethyl)benzenesulfonate substrate.……...S24
Figure S19. Kinetics of TsaC with a 4-(hydroxymethyl)benzoate substrate.……................S25
Figure S20. TsaD can be recombinantly expressed and purified.…………………………...S26
Figure S21. TsaD transforms 4-formylbenzenesulfonate into 4-sulfobenzoate …………... S27
Figure S22. TsaD transforms 4-formylbenzoate into 1,4-benzenedicarboxylate …………S28
Figure S23. Kinetics of TsaD with a 4-formylbenzenesulfonate substrate.…………………S29
Figure S24. Kinetics of TsaD with a 4-formylbenzoate substrate.…………………………...S30

Figure S25. A catalytically important Cys residue is involved in TsaD………………….…S31
Figure S26. A C286A variant of TsaD is inactive……………………………………………..S32
Figure S27. TsaC performs chemistry on a 3-(hydroxymethyl)benzoate substrate………S33
Figure S28. TsaC is unable to perform chemistry on 2-(hydroxymethyl)benzoate……….S34
Figure S29. TsaC can perform chemistry on 4-aminobenzyl alcohol……….....................S35
Figure S30. TsaC shows activity when provided with a 4-nitrobenzyl alcohol substrate.…S36
Figure S31. TsaC performs chemistry on a benzyl alcohol substrate………………………S37
Figure S32. TsaC performs chemistry on a 4-isopropylbenzyl alcohol substrate…………S38
Figure S33. TsaC performs chemistry on a 4-chlorobenzyl alcohol substrate……………..S39
Figure S34. TsaC performs chemistry on a 4-(2-hydroxyethyl)benzoate substrate………S40
Figure S35. TsaC performs chemistry on a 4-(1-hydroxyethyl)benzoate substrate………S41
Figure S36. TsaC performs chemistry on a 5-hydroxypentanoate substrate……………...S42
Figure S37. TsaC can be engineered to accept a *meso*-2,3-butanediol substrate………..S43
Figure S38. Active site residues ‘gate’ substrate binding in BDH…………………………..S44
Figure S39. A conserved role of the gating position in substrate recognition………………S45
Figure S40. The purity of TsaC and its variants was assessed using SDS-PAGE……….S47
Figure S41. Thermal stability of wild-type TsaC with benzoate molecules………………...S48
Figure S42. Thermal stability of wild-type and Y155F at high concentration………………S49
Figure S43. Thermal stability of Y155F TsaC with benzoate molecules…………………...S50
Figure S44. Thermal stability of Y155F TsaC with benzenesulfonate……………………...S51
Figure S45. Thermal stability of Y155F TsaC with various substrates……………………..S52
Figure S46. Thermal stability of T143Q/G149N TsaC with meso-2,3-butanediol…………S53
Figure S47. Thermal stability of native and Y155F TsaC with NADH and $NADP^+$ S54
Figure S48. Dynamic light scattering experiments support tetrameric state………………..S55
Figure S49. TsaM and VanB were recombinantly expressed and purified ………………...S56
Figure S50. Enzyme-mediated production of 4-sulfobenzoate……………………………...S57
Figure S51. Enzyme-mediated production of 1,4-benzenedicarboxylate………………… S58
Figure S52. Iterative formation of products from 4-methylbenzoate………………………..S59
Figure S53. TsaM and VanB consume NADH and TsaC and TsaD regenerate NADH.….S60
Figure S54. NADH consumption in reactions that contain 4-methylbenzoate..……………S61
Figure S55. Bioinformatic analysis of the SDR enzyme family……………………………...S62
Figure S56. Logo diagram of co-occurring SDR enzymes………….................................S63
Figure S57. Sequence alignment of CymB and TsaC………………………………………...S64
Figure S58. Product standard curves were used to quantify enzymatic assays.…….........S65
Figure S59. Substrate standard curves were used to quantify enzymatic assays……….. S67

III. References **S69**

Table S1. Data processing and refinement statistics for X-ray crystallography.

	TsaC
**Data collection**	
Space group	$P\ 3_221$
Cell dimensions	
a, b, c (Å)	116.01, 116.01, 66.40
α, β, γ (°)	90, 90, 120
Resolution (Å)	50.00 – 2.18 (2.26 – 2.18)
R_{meas} (%)	7.2 (150.7)
I / σ	24.18 (1.98)
Completeness (%)	99.9 (99.5)
Redundancy	16.68 (14.87)
$CC_{1/2}$	100.0 (72.4)
**Refinement**	
Resolution (Å)	43.59 – 2.18
Unique reflections	26991 (2670)
R_{work} / R_{free}	0.2085 / 0.2283
No. atoms	3241
Protein	3157
Water	84
**B-factors**	
Overall	62.28
Protein	62.23
Water	64.22
**R.M.S. deviations**	
Bond lengths (Å)	0.010
Bond angles (°)	1.370

Values in parentheses are for the highest-resolution shell (2.26-2.18 Å).

Table S2. Primers for performing site-directed mutagenesis on TsaC and TsaD. The primers marked by an asterisk were used to obtain the TsaC variant with two mutations.

**Primer name**	**Primer DNA sequence**
TsaC_Y155F	5'-cctgacctggtttagcggcagcaagg-3'
TsaC_T143Q	5'-cgtggcgagccaaaccggtgtgc-3'
TsaC_G149N	5'-tgtgcgtccgaacccgggcctgac-3'
TsaC_T143Q*	5'-gttaacgtggcgagccagaccggtgtgcgtccg-3'
TsaC_G149N*	5'-cggtgtgcgtccgaatccgggcctgacc-3'
TsaD_C286A	5'-aggtcaggccgccctggccccga-3'

Table S3. Substrates used in this work. Class 1 compounds are the reported native substrates and products (1-3) of TsaC and TsaD. Class 2 compounds are the molecules used to evaluate the substrate scope accepted by TsaC and TsaD. The asterisk (*) indicates that no commercial standard is available.

	CLASS	NAME		CLASS	NAME
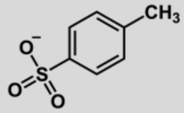	1	4-methylbenzenesulfonate	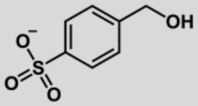	1	4-(hydroxymethyl)benzenesulfonate
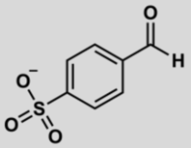	1	4-formylbenzenesulfonate	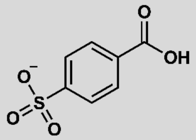	1	4-sulfobenzoate
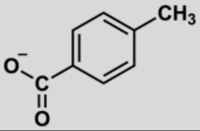	1	4-methylbenzoate	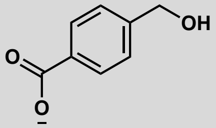	1	4-(hydroxymethyl)benzoate
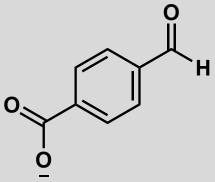	1	4-formylbenzoate	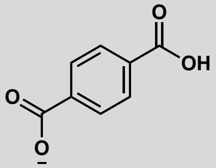	1	1,4-benzenedicarboxylate
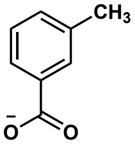	2	3-methylbenzoate	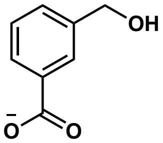	2	3-(hydroxymethyl)benzoate
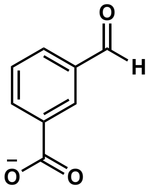	2	3-formylbenzoate	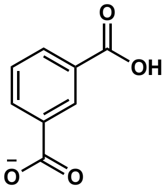	2	2,4-benzenedicarboxylate
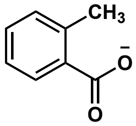	2	2-methylbenzoate	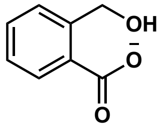	2	2-(hydroxymethyl)benzoate
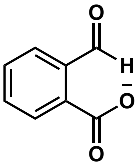	2	2-formylbenzoate	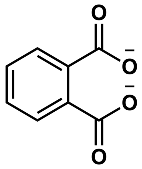	2	1,2-benzenedicarboxylate
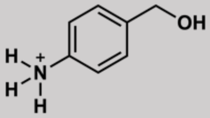	2	4-aminobenzyl alcohol	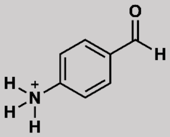	2	4-aminobenzyl aldehyde

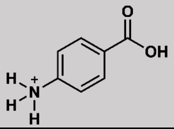	**2**	4-aminobenzoate	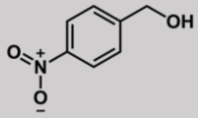	**2**	4-nitrobenzyl alcohol
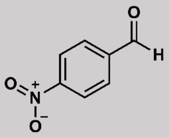	**2**	4-nitrobenzaldehyde	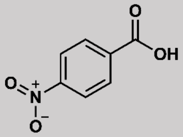	**2**	4-nitrobenzoate
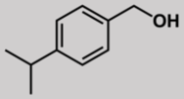	**2**	4-isopropylbenzyl alcohol	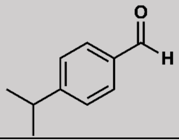	**2**	4-isopropylbenzaldehyde
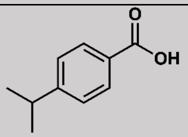	**2**	4-isopropylbenzoate	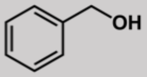	**2**	benzyl alcohol
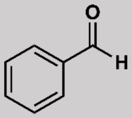	**2**	benzaldehyde	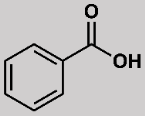	**2**	benzoate
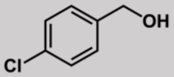	**2**	4-chlorobenzyl alcohol	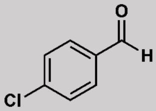	**2**	4-chlorobenzaldehyde
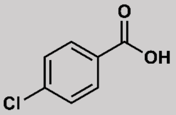	**2**	4-chlorobenzoate	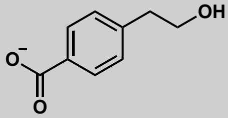	**2**	4-(2-hydroxyethyl)benzoate
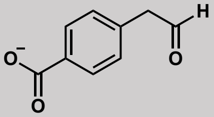	**2**	4-(2-oxoethyl)benzoate	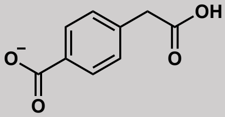	**2**	4-(carboxymethyl)benzoate
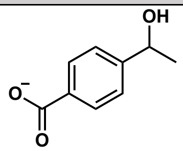	**2**	4-(1-hydroxyethyl)benzoate	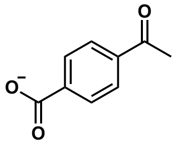	**2**	4-acetylbenzoate
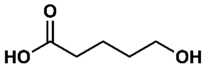	**2**	5-hydroxypentanoate	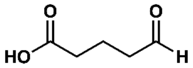	**2**	5-oxopentanoate*
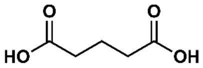	**2**	glutarate*	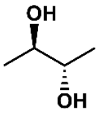	**2**	*meso*-2,3-Butanediol
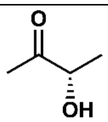	**2**	3-hydroxy-2-butanone			

.

Table S4. Melting temperatures of wild-type TsaC determined using thermal shift assays.

**Protein**	**Thermal Shift T_m (°C)**	**ΔT_m (°C)**
TsaC	54.7 ± 0.02	-
TsaC + NAD^+	54.4 ± 0.1	+0.1
TsaC + $NADP^+$	54.3 ± 0.03	-0.4
TsaC + NADH	67.1 ± 0.1	+12.4
TsaC + NAD^+ + 4-(hydroxymethyl)benzoate	56.2 ± 0.1	+1.4
TsaC + NAD^+ + 4-formylbenzoate	54.6 ± 0.3	-0.2
TsaC + NAD^+ + 1,4-benzenedicarboxylate	54.6 ± 0.2	-0.2
TsaC + NAD^+ + *meso*-2,3-butanediol	53.6 ± 0.2	-1.1

Table S5. Size exclusion chromatography multiangle light scattering (SEC-MALS) peaks of Y155F TsaC with corresponding experimental molecular weights and percent mass fraction for each peak. The hypothetical molecular weight of the monomer is 28.7 kDa, the dimer is 57.4 kDa, and the tetramer is 114.8 kDa. The approximate concentration of the protein eluting at the apex of the largest protein peak eluting from the column was approximately 0.3 μM (calculated for peak 3 of the Y155F TsaC run). The tail of the third peak partially coelutes with the solvent peak which limits the calculation for molecular weight at the peak tail and is therefore excluded. The mass fraction thus correlates to the area of the three peaks analyzed. The buffer for running these assays is 50 mM HEPES (pH 8.2), 200 mM NaCl, 5% (v/v) glycerol, and 2 mM NAD(H) as indicated.

	Peak 1		Peak 2		Peak 3	
	Mw (kDa)	Mass fraction (%)	Mw (kDa)	Mass fraction (%)	Mw (kDa)	Mass fraction (%)
Y155F TsaC	110.6	5.17	106.5	34.3	107.0	48.8
Y155F TsaC + NAD^+	107.8	34.3	108.8	48.9	105.0	16.8
Y155F TsaC + NADH	112.2	19.2	109.6	35.5	108.9	45.3

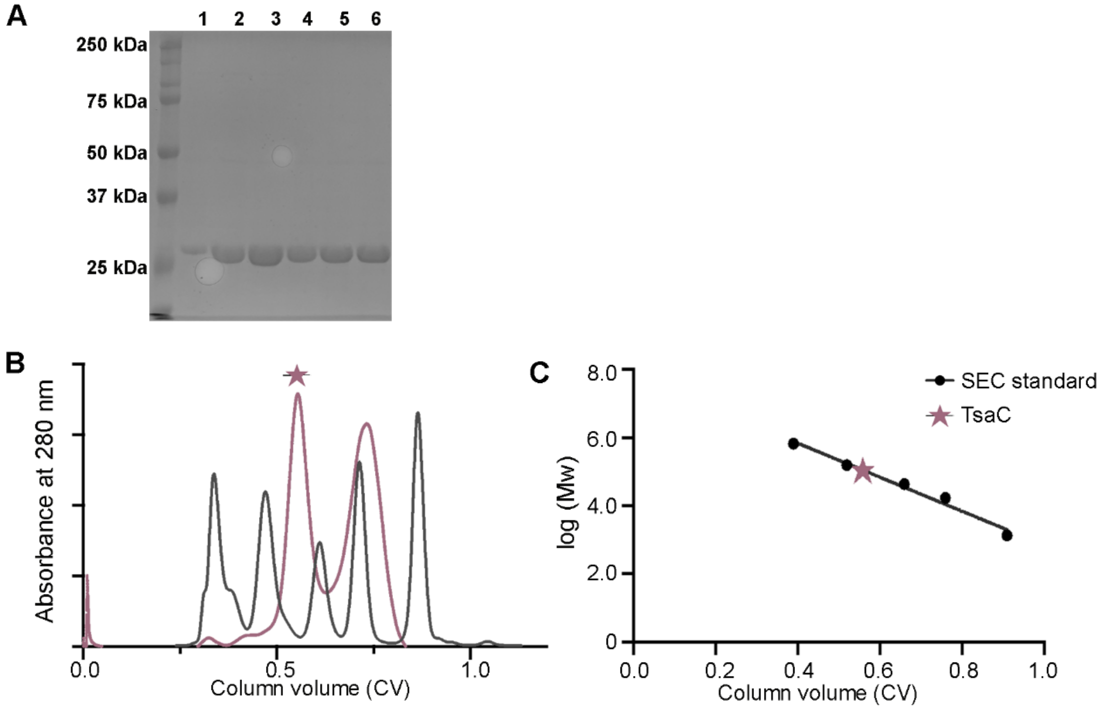

Figure S1. TsaC can be recombinantly expressed and purified using nickel affinity and gel filtration chromatography. (**A**) SDS-PAGE was used to assess the purity of His-tagged TsaC. The molecular mass of His-tagged TsaC is approximately 27 kDa and was judged by comparison with a protein standard (left lane). (**B**) Using gel filtration chromatography, it was determined that TsaC elutes in two peaks that correspond to both monomeric and tetrameric states. (**C**) The molecular weight of the eluted fractions of TsaC were estimated using a plot of standard molecular weight versus retention time.

```
TsaC      MNLNKQVAIVTGGASGFGAAIARRLSQAGAAVLVADLNAEGAQRMATELNAAGGRALGMA      60
BDH       ---MKKVALVTGAGQGIGKAIALRLVKDGFAVAIADYNDATAKAVASEINQAGGHAVAVK      57
              *:**:***...*:* *** ** : * ** :** *   *: :*:*:* ***:*:.:

TsaC      CDVSKEADYRAVVDAAIAQLGGLHIVVNNAGTTHRNKPALAVTEDEFDRVYRVNLKSVYW      120
BDH       VDVSDRDQVFAAVEQARKTLGGFDVIVNNAGVAP-STPIESITPEIVDKVYNINVKGVIW      116
           ***.. :  *.*: *   ***:.::*****.:  ..*  ::* : .*:**.:*:*.* *

TsaC      SAQCALPHFAQQGHGV-MVNVASTTGVRPGPGLTWYSGSKAAMINLTKGLALEFARSGVR      179
BDH       GIQAAVEAFKKEGHGGKIINACSQAGHVGNPELAVYSSSKFAVRGLTQTAARDLAPLGIT      176
          . *.*:  * ::***  ::*..* :*   .* *: **.** *: .**:  * ::*  *:

TsaC      INAVNPMIGETPMMADFMGMEDT------PANRERFLSRIPLGRFTRPDDVASAVAFLAS      233
BDH       VNGYCPGIVKTPMWAEIDRQVSEAAGKPLGYGTAEFAKRITLGRLSEPEDVAACVSYLAS      236
          :*.  * * :*** *::    .         .  .* .** ***::.*:***:.*::***

TsaC      DDASFLTGVCLDVDGGRNI-    252
BDH       PDSDYMTGQSLLIDGGMVFN    256
           *:.::** .* :***  :
```

Figure S2. A sequence alignment of TsaC and *meso*-2,3-butanediol dehydrogenase (BDH, PDB: 1GEG (4)) reveals the presence of conserved sequence motifs for binding NAD(H) and facilitating catalysis. Namely, TsaC contains the TGX$_3$GXG, NNAG, and characteristic Asp33 and Asp62 residues for binding NAD(H), rather than NADP(H), and interacting with NAD(H), respectively (purple). In addition, the catalytic tetrad of residues, Asn114, Ser142, Tyr155, and Lys159 are shown in blue. The 30-residue gap in our structure of TsaC corresponds to the so-called substrate binding loop and is highlighted in gray (residues 188-220 and 187-220 in Chains A and B, respectively). The residues targeted for mutagenesis in this work are shown in dark orange. Pro residues implicated in anchoring the substrate binding loop in other SDR enzymes are shown in green. In TsaC, Pro185 caps β6 and is part of the previously reported as the PGX$_3$T motif of classical SDR enzymes (5).

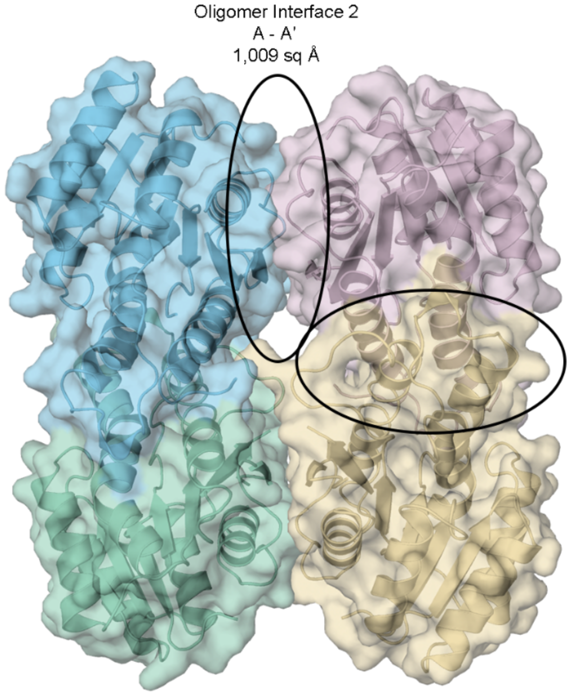

Figure S3. TsaC exists as tetramer *in crystallo*. The probability of biological relevance of the observed tetramer was assessed by calculating solvent accessible buried surface area using both PISA and PyMol (6). Since both programs calculated similar areas, the PISA results are listed here. Oligomer interface 1 is formed between α5 and α6 of both chains A and B of the same asymmetric unit. This interface contains approximately 2,830 sq Å of buried surface area. Oligomer interface 2, which is formed between α7, β7, and the C-terminal residues of symmetry related chains, contains 1,009 sq. Å of buried surface area. The total buried surface area within the tetrameric unit is 7,780 sq. Å, which corresponds to roughly 23.8% of the total combined surface area of the four polypeptides and indicates that the tetramer is very likely to be of biological relevance. Each polypeptide is colored individually, and the discussed interfaces are circled. The PISA tool (6) was used to estimate that the free energy of assembly dissociation (ΔG^{Diss}) from the homotetramer to the monomer state is approximately 4.4 kcal/mol. The free energy of assembly dissociation, from the homodimer to the monomer state is estimated, using the same tool (6), to be approximately 17.8 kcal/mol. Of note, values of ΔG^{Diss} greater than 0 indicate a stable complex in solution

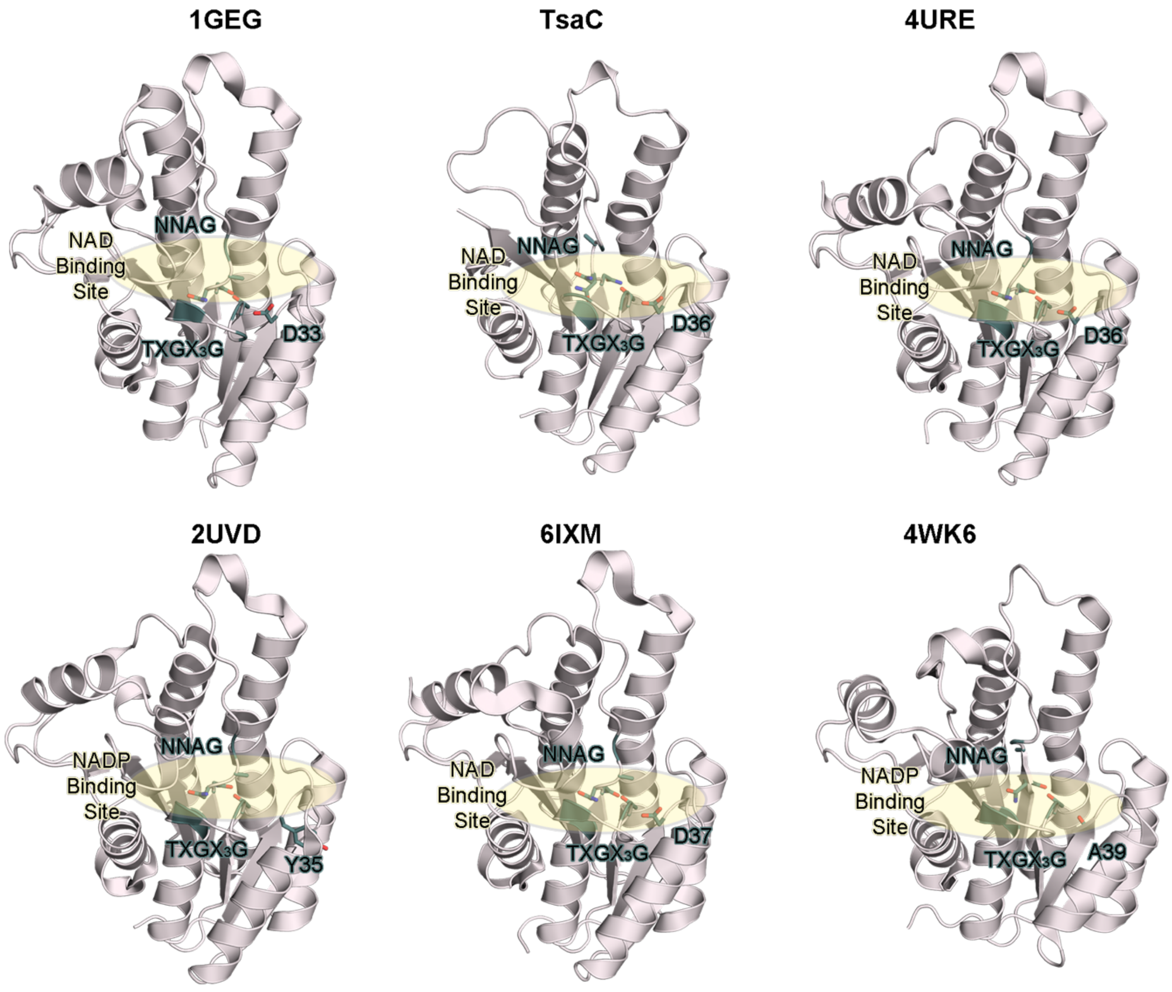

Figure S4. A conserved SDR-type NAD(H)/NADP(H) binding pocket is illustrated in various SDR structures. TsaC, the molecular replacement model *meso*-2,3-butanediol dehydrogenase (BDH, percent identity 39.11%, PDB: 1GEG) (4), and similar structures identified using the DALI server, including: 1-(4-hydroxyphenyl)-ethanol dehydrogenase (Hped, percent identity 31.71%, PDB: 4URE) (7), 3-oxoacyl-(acyl carrier protein) reductase (percent identity 36.07%, PDB: 2UVD) (8), *Ch*KRED20 (percent identity 33.33%, PDB: 6IXM) (9), and 3-ketoacyl-(acyl-carrier-protein) reductase (percent identity 36.51%, PDB: 4WK6) (10) were compared for conserved NAD(P)(H) binding residues. The TGX_3GXG and NNAG motifs near the N-terminus of SDR enzymes form a binding pocket for NAD(H)/NADP(H) binding. The TGX_3GXG forms the loop between β1 and α1, stabilizes the core of the protein through hydrogen bonding, and provides space with small Gly residues for NAD(H)/NADP(H), binding. The NNAG motif stabilizes β4 and again provides room for NAD(H)/NADP(H). The presence of a negatively charged residue, aligned with TsaC Asp36, typically favors NAD(H) rather than NADP(H), though the position of this residue can vary slightly depending on the subclass of classical SDR enzyme (11).

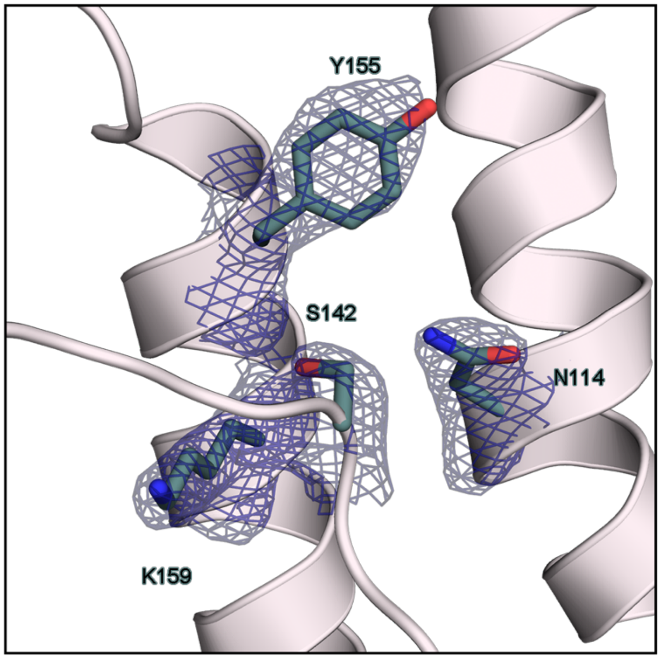

Figure S5. In the absence of NAD$^+$, the TsaC catalytic tetrad (Asn114, Ser142, Tyr155 and Lys159) of residues can be confidently modeled in an inactive orientation. 2F_o-F_c simulated annealing composite omit electron density maps are contoured to 1 σ around the catalytic tetrad residues and are shown in blue. Residues in the catalytic tetrad of TsaC are labeled. The B-factor for the residues within the tetrad (chain A: Asn110 = 49 Å^2, Ser142 = 63 Å^2, Tyr152 = 50 Å^2 and Lys156 = 45 Å^2) are similar to surrounding residues and are near or below the average B-factor for the residues in the structure (protein residues = 61 Å^2).

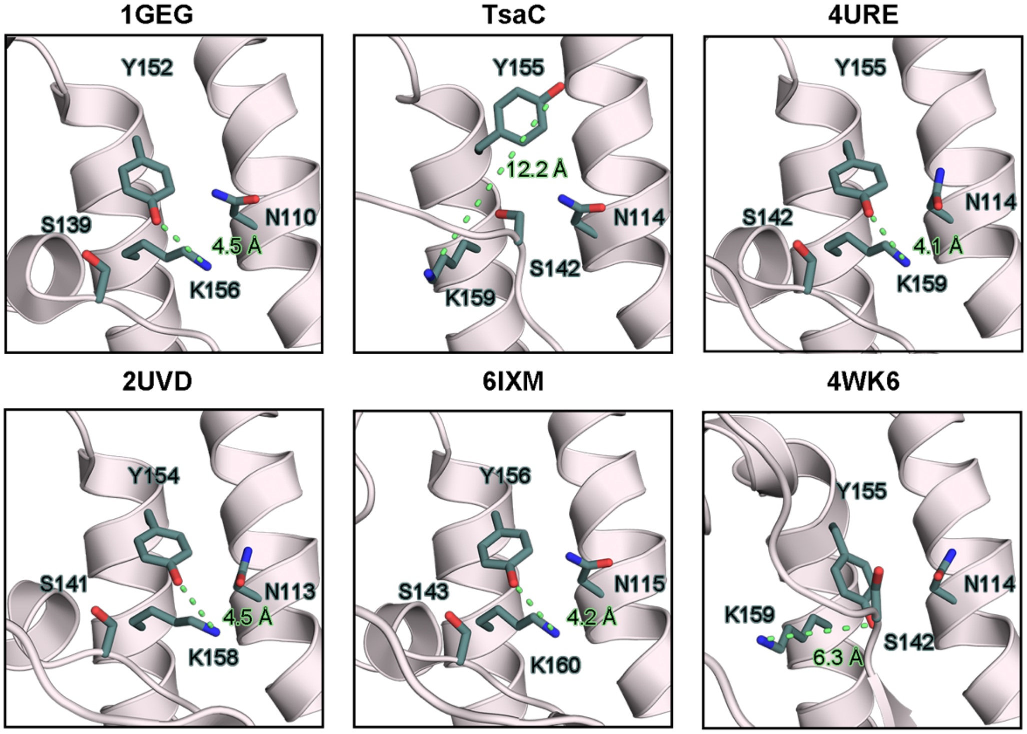

Figure S6. The catalytic tetrad of TsaC and SDR enzymes with similar structures showcase active and inactive orientations. The structure of TsaC, the molecular replacement model BDH (PDB: 1GEG), and structures similar to TsaC identified using the DALI server (12) are shown as cartoon ribbons, with emphasis on the active site catalytic tetrad. A canonical SDR mechanism employs a Lys residue to lower the pK_a of the catalytic Tyr residue, so that the deprotonated Tyr residue can act as a catalytic base. This interaction is supported by the Lys residue orientation near the Tyr residue, as shown in 1GEG, 4URE, 2UVD, and 6IXM. The inactive confirmation is typically characterized by the Tyr residue flipping away from the rest of the triad, as seen in TsaC. Distances between the Tyr and Lys are highlighted as green dashed lines. Protein names: *meso*-2,3-butanediol dehydrogenase (BDH, PDB:1GEG) (4); 1-(4- hydroxyphenyl)-ethanol dehydrogenase (PDB:4URE) (7); 3-oxoacyl-(acylcarrier protein) reductase (PDB:2UVD) (8); *Ch*KRED20 (PDB:6IXM) (9); beta-ketoacyl-(acyl carrier protein) reductase (PDB: 4WK6) (10).

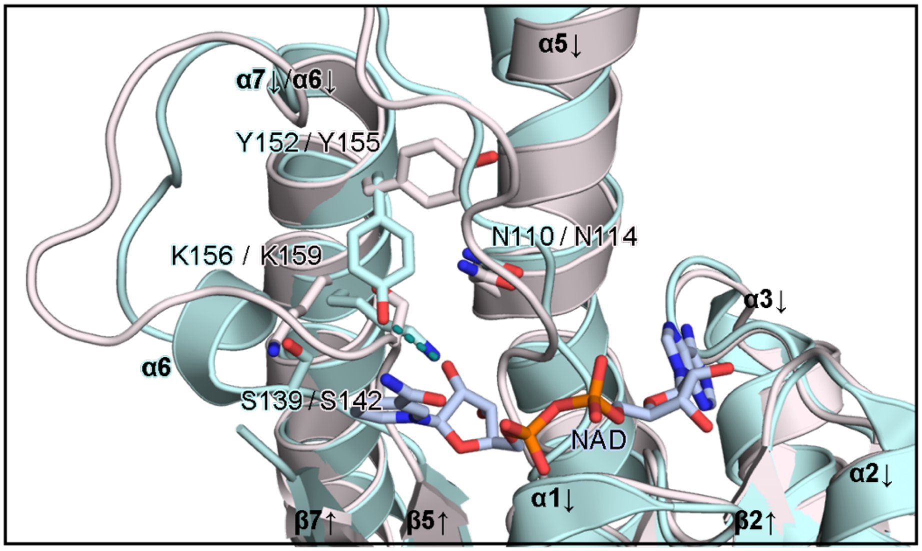

Figure S7. A conformational change in TsaC is required for NAD^+ to bind. An overlay of TsaC (lavender) with BDH (light blue, 1GEG (4)) reveals that NAD^+ would clash with the loop that connects β5 and α6. In addition, Tyr155 needs to assume an active orientation to provide the needed interaction with NAD^+. Tyr traditionally functions as the catalytic base and its pK_a is lowered though an electrostatic interaction with Lys, which also interacts with the ribose moiety of NAD^+.

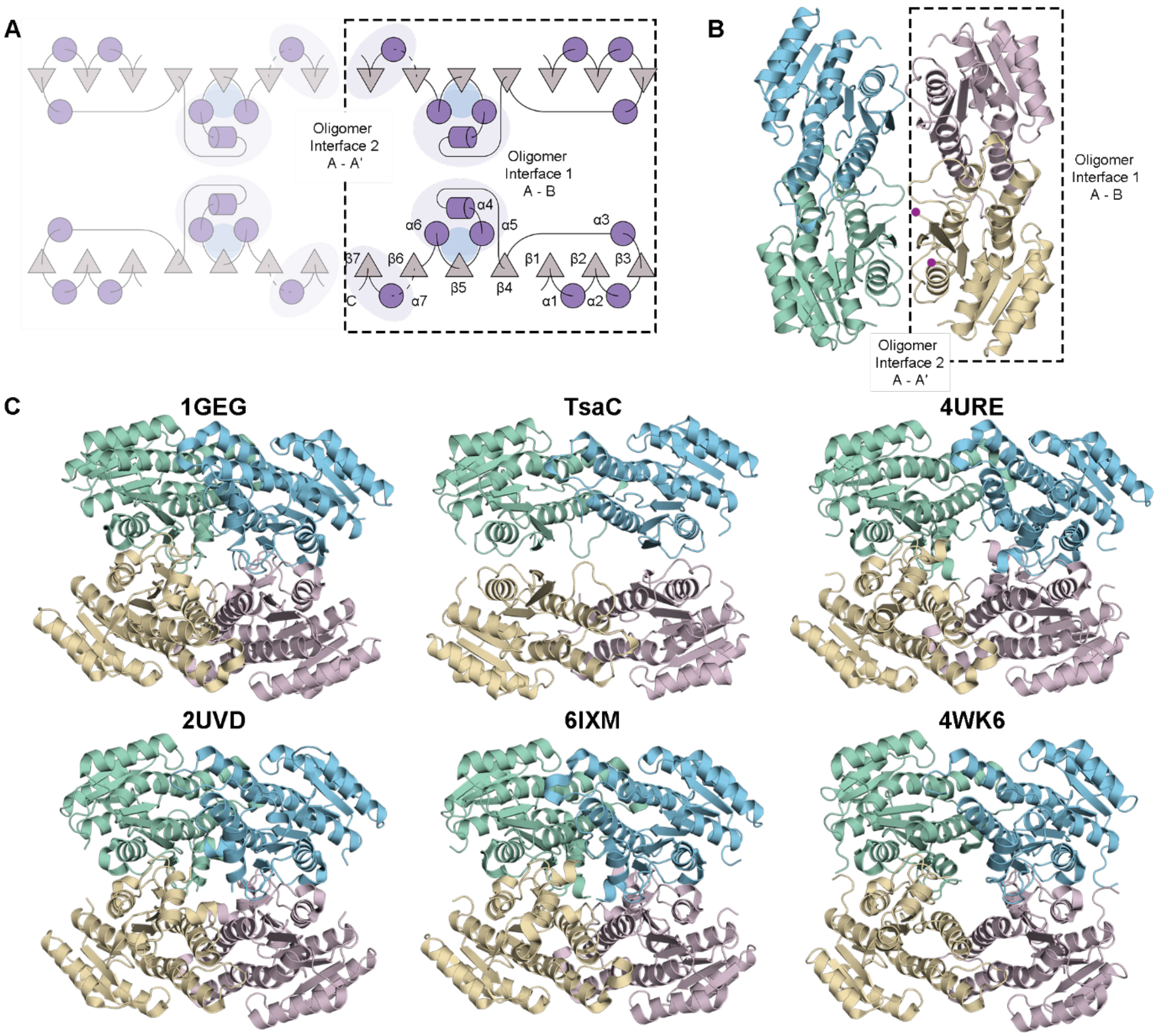

Figure S8. TsaC adopts a tetrameric oligomeric state in the crystal structure. (A) A schematic of the protein architecture reveals the central β-sheet within the Rossmann core fold of TsaC and two oligomeric interfaces. One interface is present between chain A and chain B within the asymmetric unit, and one interface is between chain A and chain A′ of the adjoining asymmetric unit. Structural elements involved in the interfaces are highlighted with purple ovals, and the location of the catalytic tetrad is highlighted with a blue circle between α5, β5, and α6. The asymmetric unit is outlined as a dashed box. (B) A cartoon diagram of the tetrameric TsaC provides context to the identified oligomer interfaces. The approximate 30 residue stretch that is unable to be modeled in any chain of the final structure, located near the center of the tetramer, is identified with purple dots on the sand-colored chain. (C) The tetrameric architecture is similar between TsaC, the molecular replacement model BDH, and similar structures identified using the DALI server. Each of these other structures contains a similar Rossmann core fold and are most varied in space in the substrate binding loop region between the region that corresponds to β6 and α7 in TsaC, near the center of the tetramer. Percent sequence identity was calculated using Clustal Omega (13) as compared to TsaC. Protein names: *meso*-2,3-butanediol dehydrogenase (BDH, PDB:1GEG) (4); 1-(4-hydroxyphenyl)-ethanol dehydrogenase (PDB:4URE) (7); 3-oxoacyl-(acylcarrier protein) reductase (PDB:2UVD) (8); ChKRED20 (PDB:6IXM) (9); beta-ketoacyl-(acyl carrier protein) reductase (PDB: 4WK6) (10).

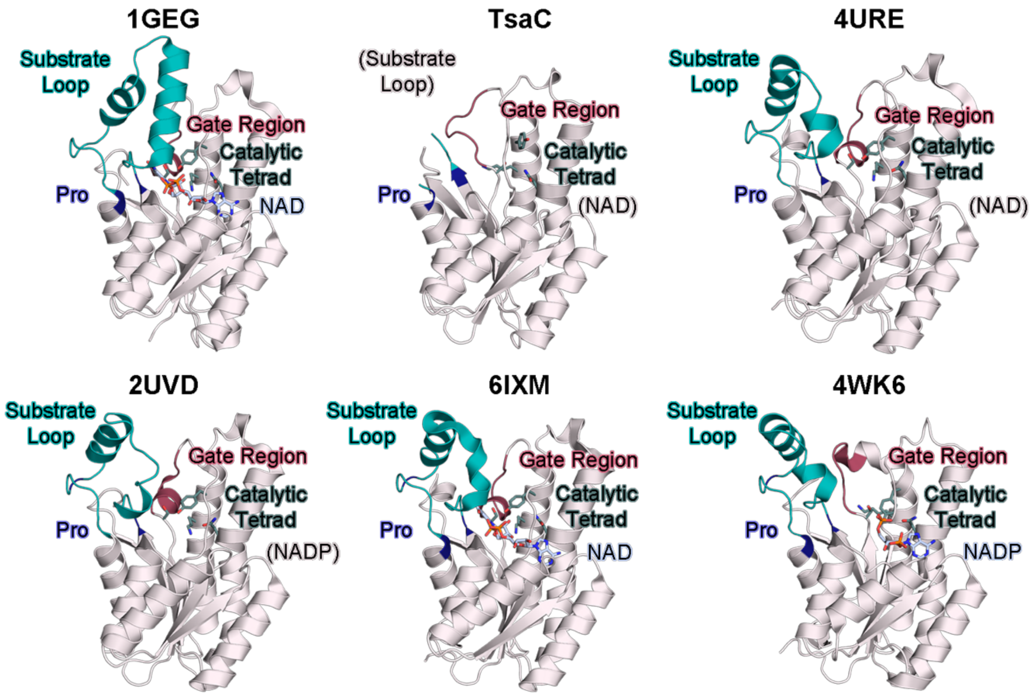

Figure S9. TsaC and SDR enzymes exhibit greater structural divergence in the substrate binding loop than in the rest of the enzyme. Despite similar overall structures, the substrate loop is seen in various structural arrangements (teal). This substrate binding loop is often bookended by Pro residues, with the first belonging to the so called PGX_3T (dark blue). The region around residues 143-149, which align with the identified salt bridge gate when comparing BDH and TsaC, here notated as the gate region, is highlighted in pink and also exhibits structural diversity. For structural context, the catalytic tetrad and NAD(H)/NADP(H) (if bound) are shown in stick representation. Residues in the substrate binding loop of TsaC are too disordered to build and are therefore missing from the structure (residues 188-220 in chain A, 187-220 in chain B). When no NAD(H)/NADP(H) is found in the structure, or in the case of TsaC when the substrate loop is not modeled, the location is identified in parentheticals. Protein names: *meso*-2,3-butanediol dehydrogenase (BDH, PDB:1GEG) (4); 1-(4- hydroxyphenyl)-ethanol dehydrogenase (PDB:4URE) (7); 3-oxoacyl-(acylcarrier protein) reductase (PDB:2UVD) (8); ChKRED20 (PDB:6IXM) (9); beta-ketoacyl-(acyl carrier protein) reductase (PDB: 4WK6) (10).

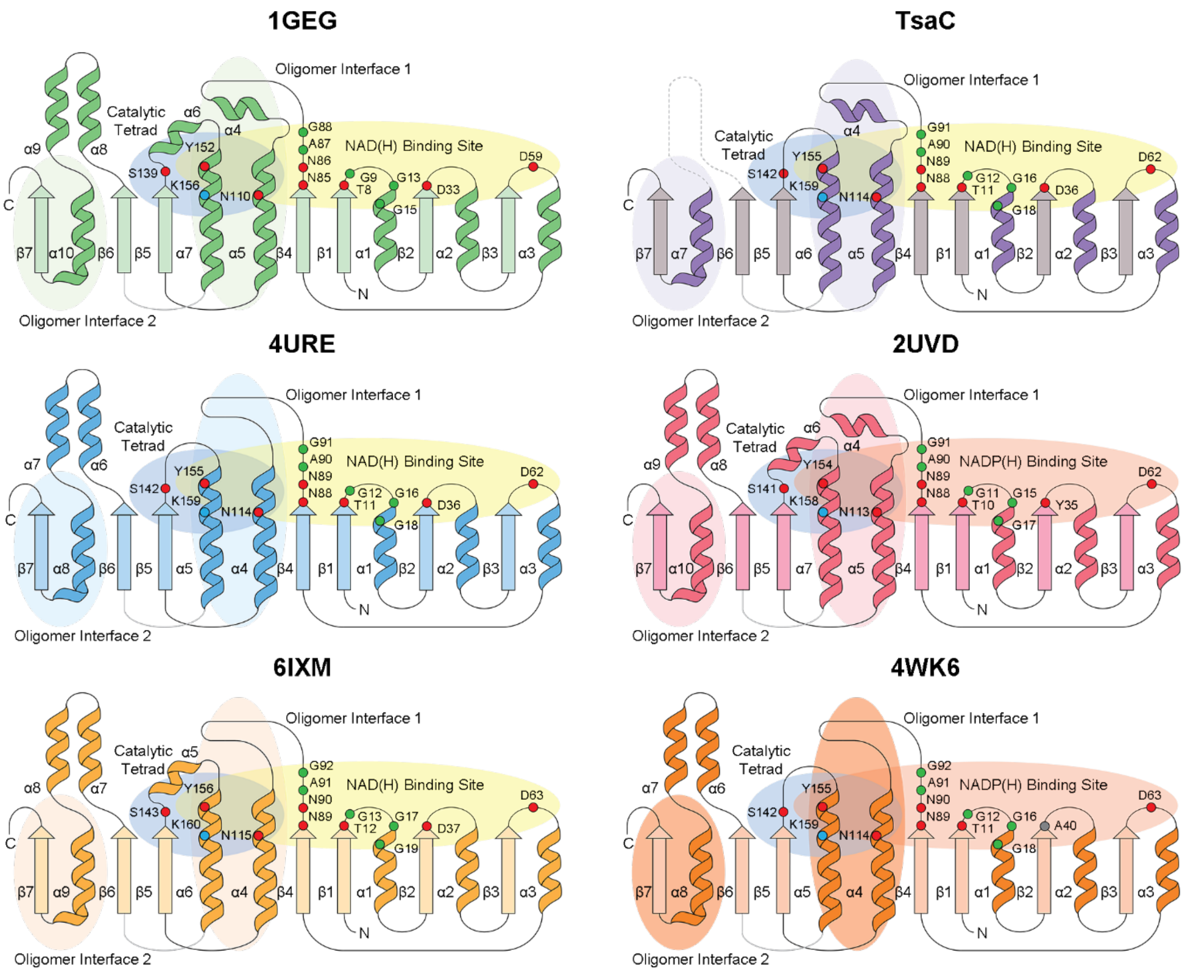

Figure S10. Topology diagrams identify conserved residues in TsaC and similar SDR enzymes. The oligomeric interfaces are highlighted as vertical shaded ovals. The active site catalytic tetrad is highlighted with a blue oval, and the NAD(H)/NADP(H) binding site is highlighted with a large yellow or red oval, respectively, encompassing the TGX_3GXG and NNAG motifs. The 3-oxoacyl-(acyl carrier protein) reductase (PDB:2UVD) (8) binds NADP(H), and contains Tyr35 in place of TsaC Asp36 to accommodate the additional phosphate of NADP(H). Each of the other structures contains two helices in the substrate binding loop, located above the catalytic tetrad. Numbering of the residues, helices, and loops correspond to the original publication of each structure where possible. Protein names: *meso*-2,3-butanediol dehydrogenase (BDH, PDB:1GEG) (4); 1-(4- hydroxyphenyl)-ethanol dehydrogenase (PDB:4URE) (7); ChKRED20 (PDB:6IXM) (9); beta-ketoacyl-(acyl carrier protein) reductase (PDB: 4WK6) (10).

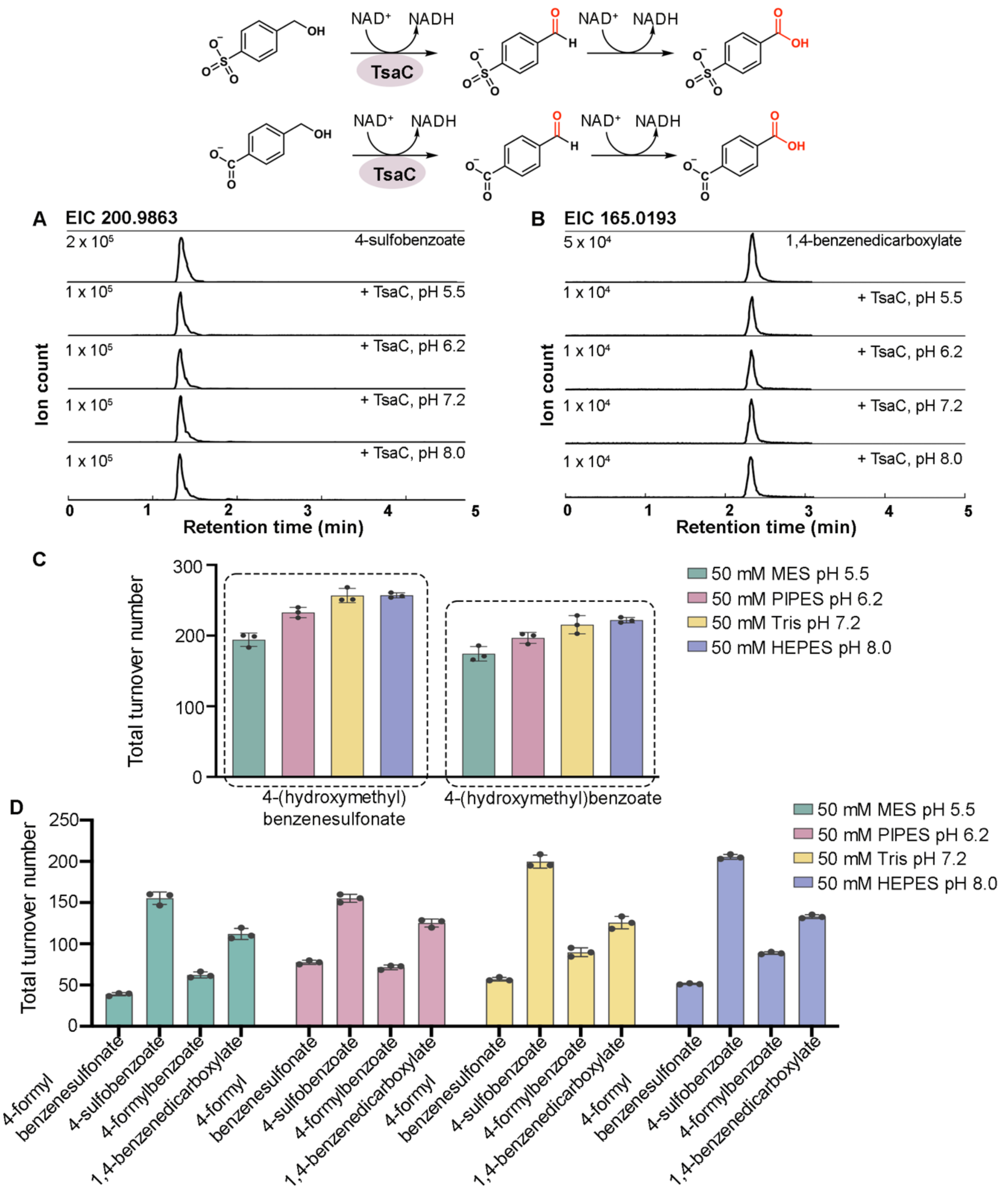

Figure S11. An optimum pH value for measuring the activity of TsaC was determined to be 7.2 for both 4-(hydroxymethyl)benzenesulfonate and 4-(hydroxymethyl)benzoate substrates. (**A**) Liquid chromatography-mass spectrometry (LC-MS) experiments were used to measure product formation when TsaC was incubated with 4-(hydroxymethyl)benzenesulfonate and NAD^+ at a range of different pH values. (**B**) Similar to that described in panel A, LC-MS experiments were used to measure product formation when TsaC was incubated with 4-(hydroxymethyl)benzoate and NAD^+ at a range of different pH values. (**C**) A plot of the data shown in panels A and B reveals that a pH value of 7.2 supports the optimum level of activity with both 4-(hydroxymethyl)benzenesulfonate and 4-(hydroxymethyl)benzoate substrates. (**D**) At each tested pH value, the distribution of the formed aldehyde and carboxylic acid products is similar.

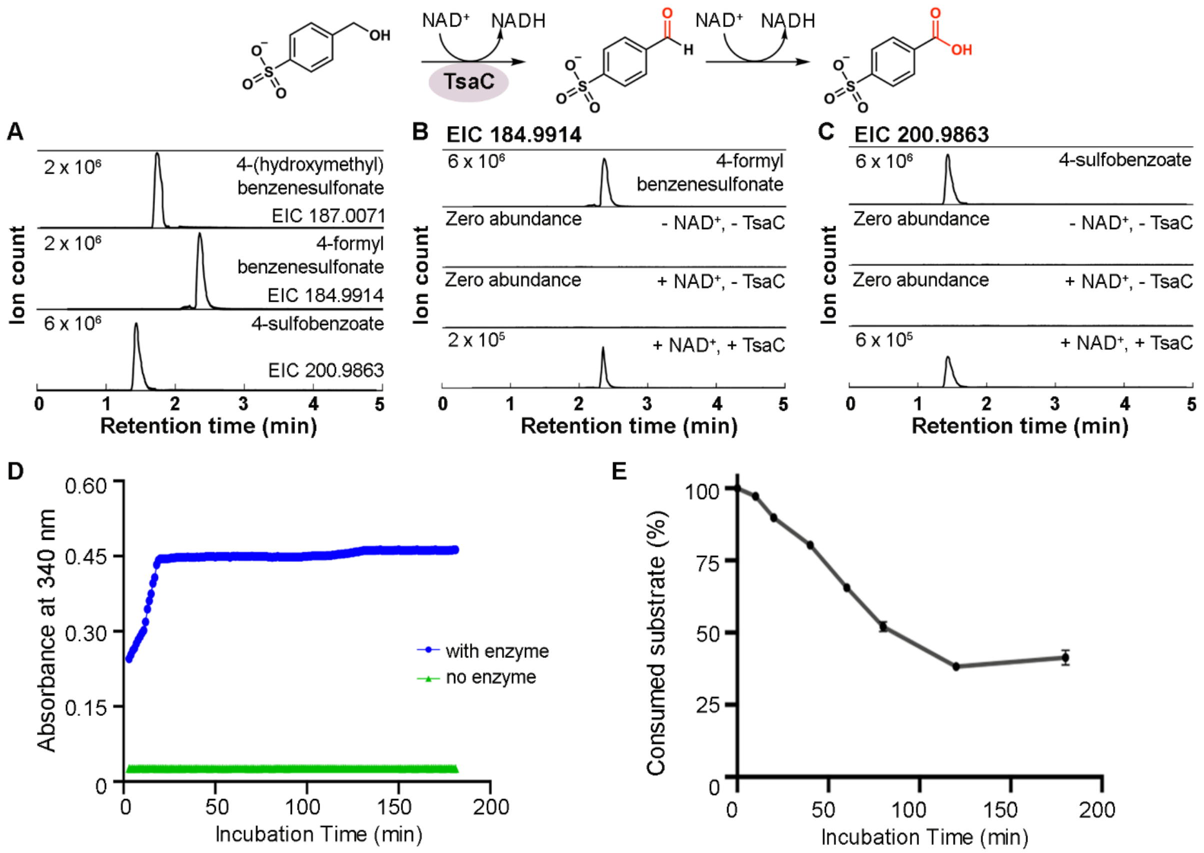

Figure S12. TsaC transforms 4-(hydroxymethyl)benzenesulfonate into 4-formylbenzenesulfonate and 4-sulfobenzoate products. (**A**) Commercially purchased substrate and product standards were used to evaluate the enzymatic activity of TsaC. (**B**) TsaC produces 4-formylbenzenesulfonate when incubated with a 4-(hydroxymethyl)benzenesulfonate substrate. This transformation requires NAD^+. (**C**) Similar to that shown in panel B, when TsaC is incubated with a 4-(hydroxymethyl)benzenesulfonate substrate and NAD^+, production of 4-sulfobenzoate is observed. (**D**) The absorbance at 340 nm was monitored to verify that NADH is produced in the TsaC-catalyzed reaction. (**E**) The extracted ion chromatogram for the product formed by TsaC with 4-(hydroxymethyl)benzenesulfonate revealed that the optimal incubation time is 2 h for measuring total turnover numbers (TTNs). The y-axis in this plot was calculated using substrate consumption with the substrate standard curve shown in Figure S59.

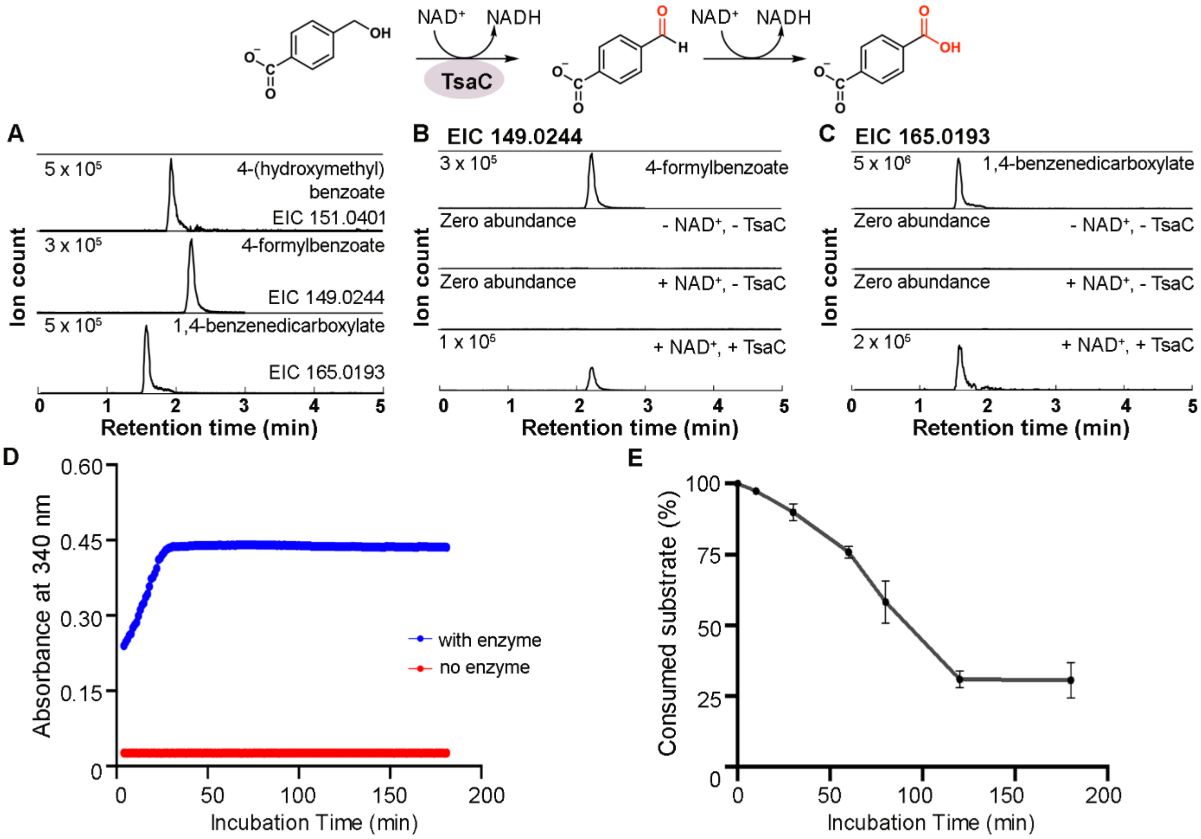

Figure S13. TsaC transforms 4-(hydroxymethyl)benzoate into both the corresponding aldehyde (4-formylbenzoate) and carboxylic acid (1,4-benzenedicarboxylate) products. (**A**) Commercially purchased standards of 4-(hydroxymethyl)benzoate, 4-formylbenzoate, and 1,4-benzenedicarboxylate were used to evaluate the enzymatic activity of TsaC. (**B**) TsaC, with the assistance of NAD^+, produces 4-formylbenzoate when incubated with a 4-(hydroxymethyl)benzoate substrate. (**C**) Similar to that shown in panel B, TsaC transforms 4-(hydroxymethyl)benzoate into 1,4-benzenedicarboxylate in an NAD^+-dependent reaction. (**D**) The absorbance at 340 nm was monitored to verify that NADH is produced in the TsaC-catalyzed reaction. (**E**) The extracted ion chromatogram for the product formed by TsaC with 4-(hydroxymethyl)benzoate revealed that the optimal incubation time is 2 h for measuring TTNs. The y-axis in this plot was calculated using substrate consumption with the substrate standard curve in Figure S59.

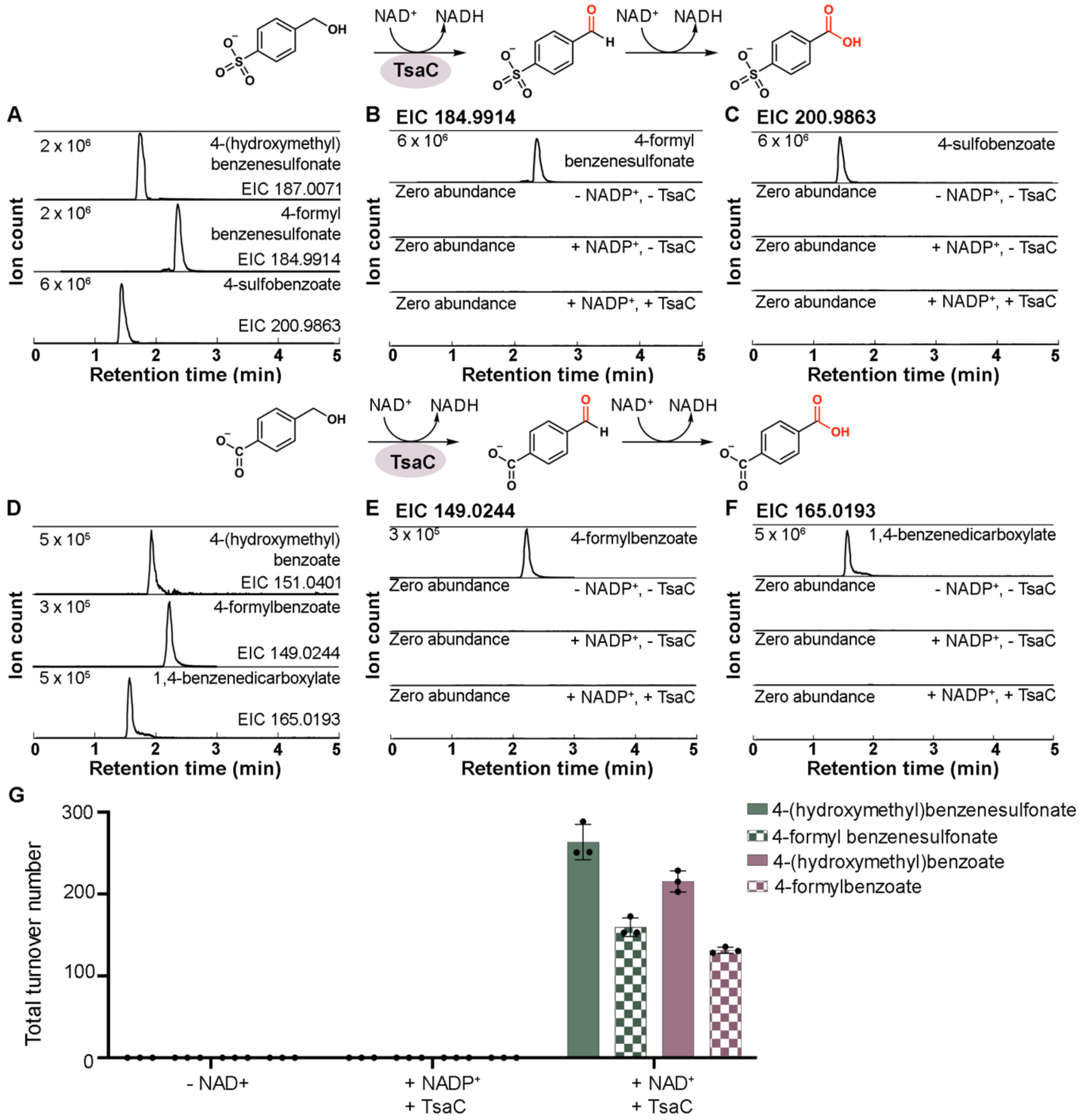

Figure S14. $NADP^+$ does not support the activity of TsaC. (**A**) LC-MS was used to separate the substrates and products of the reaction. This panel indicates that commercially purchased substrate of product standards of the tested set of sulfonate molecules can be separated and ionized using LC-MS. (**B**) The combination of TsaC, $NADP^+$, and 4-(hydroxymethyl)benzenesulfonate does not result in formation of 4-sulfobenzoate. (**C**) Similarly, incubation of TsaC, $NADP^+$, and 4-(hydroxymethyl)benzenesulfonate does not result in production 4-sulfobenzoate. (**D**) An LC-MS method was developed to separate 4-(hydroxymethyl)benzoate from its corresponding aldehyde and acid products. (**E**) As described for the 4-(hydroxymethyl)benzenesulfonate substrate, $NADP^+$ does not support an ability of TsaC to transform 4-(hydroxymethyl)benzoate into 4-formylbenzoate. (**F**) Likewise, $NADP^+$ does not support formation of 1,4-benzenedicarboxylate. (**G**) A bar graph shows the total turnover number of TsaC with four different provided substrates. This graph reveals that only NAD^+, and not $NADP^+$, supports TsaC catalysis. The data in this panel were measured using n = 3 independent experiments and are represented as the mean value of those measurements. The traces in panel (A) and (D) are the same as traces in Figure S12A and S13A, respectively, and are reproduced here for clarity.

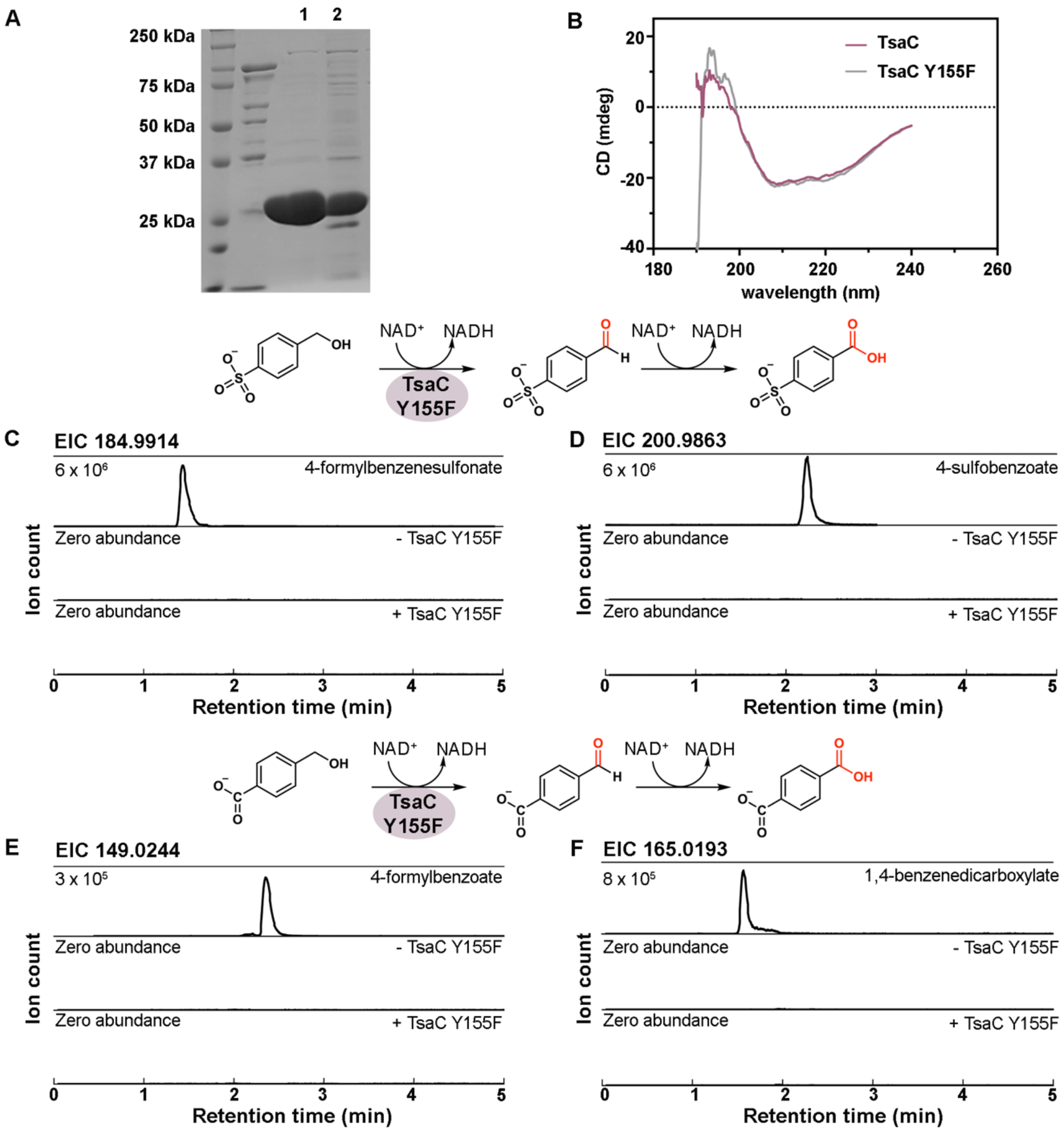

Figure S15. A Y155F variant of TsaC is not active on either 4-(hydroxymethyl)benzenesulfonate or 4-(hydroxymethyl)benzoate substrates. (**A**) The Y155F variant of TsaC was recombinantly expressed and purified. According to SDS-PAGE, the His-tagged variant, as expected, has a molecular weight of approximately 27 kDa. (**B**) A circular dichroism (CD) experiment indicates that both wild-type TsaC and Y155F TsaC are similarly folded. (**C**) Incubation of the Y155F variant with NAD^+ and 4-(hydroxymethyl)benzenesulfonate does not result in formation of 4-formylbenzenesulfonate. (**D**) As observed in panel C, the combination of Y155F TsaC with NAD^+ with 4-(hydroxymethyl)benzenesulfonate also does not result in formation of 4-sulfobenzoate. (**E**) Incubation of the Y155F variant of TsaC with NAD^+ and 4-(hydroxymethyl)benzoate does not result in formation of 4-formylbenzoate. (**F**) As observed in panel D, the combination of Y155F TsaC with NAD^+ with 4-(hydroxymethyl)benzoate does not result in formation of 1,4-benzenedicarboxylate.

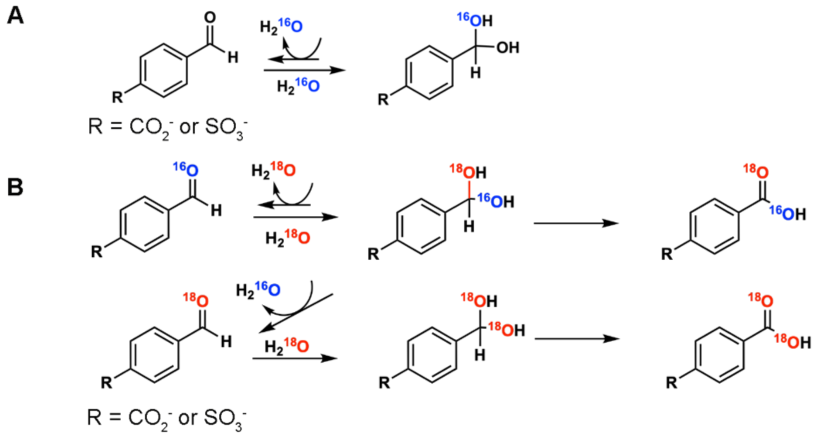

Figure S16. The origin of the carboxylic acid products (4-sulfobenzoate and 1,4-benzenedicarboxylate) detected in the TsaC-catalyzed reactions was investigated in ^{18}O-labeled water. (**A**) Incubation of either 4-formylbenzenesulfonate or 4-formylbenzoate in ^{18}O-labeled water revealed that labeled oxygen can be incorporated. (**B**) When TsaC performs chemistry in ^{18}O-labeled water, there is the potential to form a variety of differentially labeled carboxylic acid products (*see* Figure 3D-F).

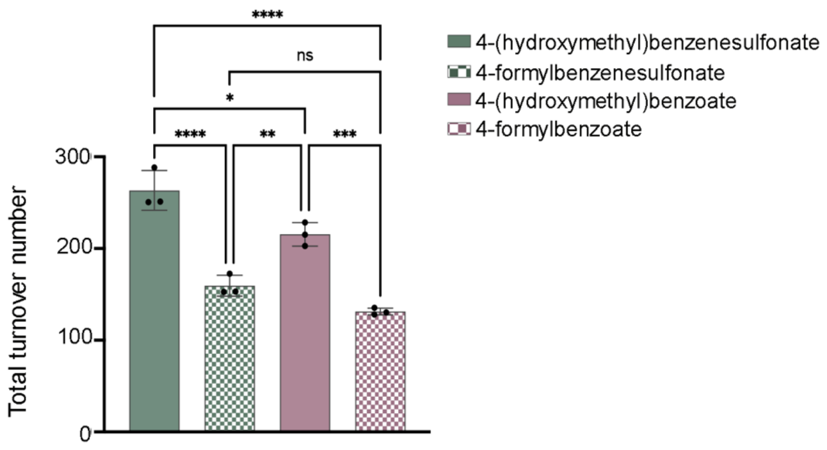

Figure S17. Total turnover number of TsaC when provided with either the alcohol- or aldehyde-containing substrates. In this plot, the amount of carboxylic acid product formed is shown. This plot reveals that the alcohol molecules support significantly higher levels of TsaC activity. The data were measured using n = 3 independent experiments and are presented as the mean value ± SD of these measurements. In this figure, ****$p < 0.0001$, ***$p < 0.001$, **$p < 0.01$, and ns indicates no significant difference from an ordinary one-way ANOVA Tukey analysis.

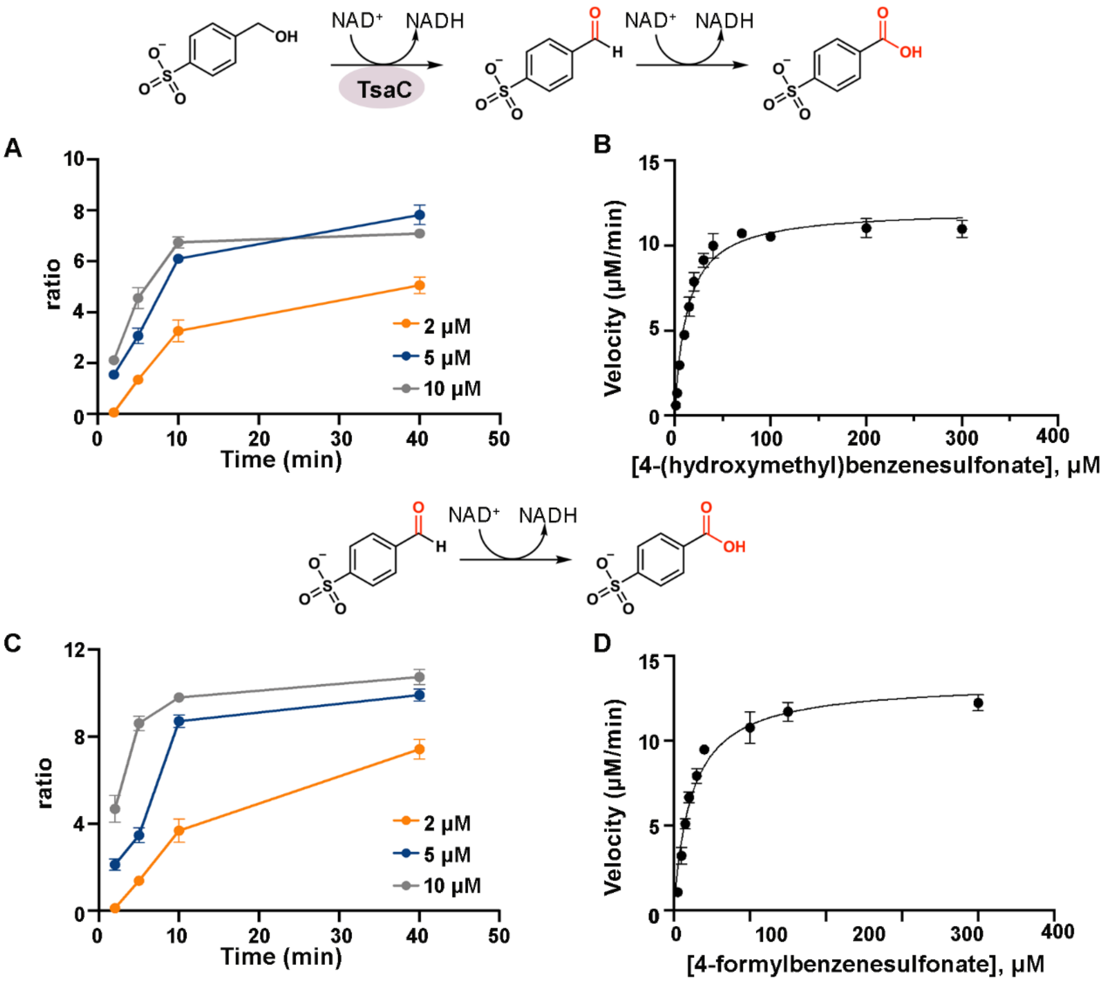

Figure S18. The steady state kinetic behavior of TsaC with a 4-(hydroxymethyl)benzenesulfonate substrate. (**A**) The linear range of 4-sulfobenzoate formation was investigated when using 4-(hydroxymethyl)benzenesulfonate as a substrate. A time of 5 min with 2 µM of TsaC was chosen for performing the kinetic assay. (**B**) Fitting of the measured data at different substrate concentrations to the Michaelis-Menten equation revealed the kinetic parameters for the TsaC-catalyzed reaction on a 4-(hydroxymethyl)benzenesulfonate substrate. (**C**) The linear range of formation of 4-sulfobenzoate was investigated using 4-formylbenzenesulfonate as a substrate. A time of 5 min with 2 µM of TsaC was chosen for measuring kinetics. (**D**) Fitting of the measured data to the Michaelis-Menten equation revealed the kinetic parameters for the TsaC-catalyzed reaction on a 4-formylbenzenesulfonate substrate. In this figure, the data were measured using n = 3 independent experiments and are represented as the mean value of those measurements. All kinetic parameters are reported in Table 1.

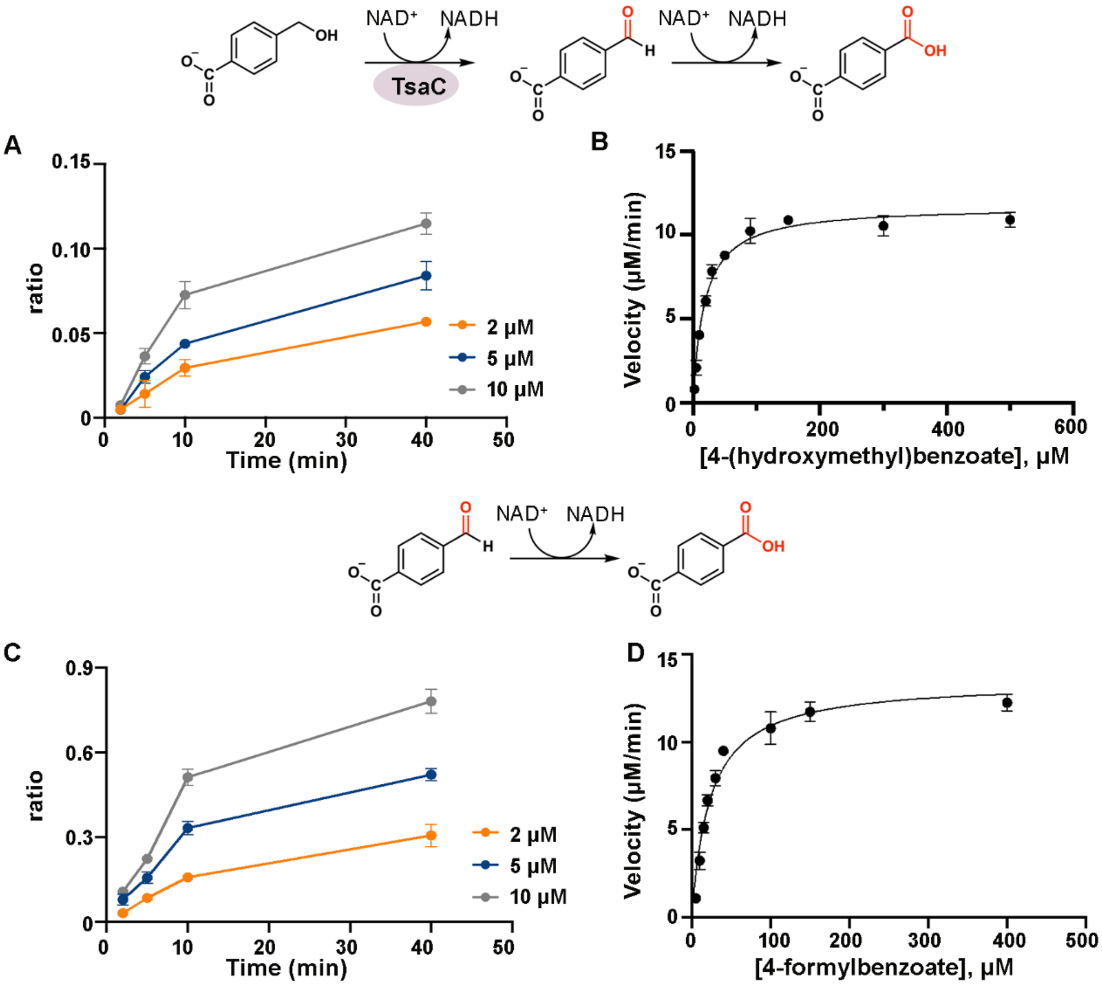

Figure S19. The steady state kinetic behavior of TsaC with a 4-(hydroxymethyl)benzoate substrate. (**A**) The linear range of 1,4-benzenedicarboxylate formation was investigated. A time of 5 min with 2.5 μM of TsaC was chosen for performing the kinetic experiment. (**B**) Following measurement of reaction velocity at different substrate concentrations, the data were fit to the Michaelis-Menten equation. (**C**) The linear range formation of 1,4-benzenedicarboxylate was investigated when using a 4-formylbenzoate substrate. A time of 5 min with 2 μM of TsaC was chosen to perform kinetic experiments. (**D**) Fitting of the measured data to the Michaelis-Menten equation revealed the kinetic parameters for the TsaC-catalyzed reaction on a 4-formylbenzoate substrate. In this figure, the data were measured using n = 3 independent experiments and are represented as the mean value of those measurements. All kinetic parameters are reported in Table 1.

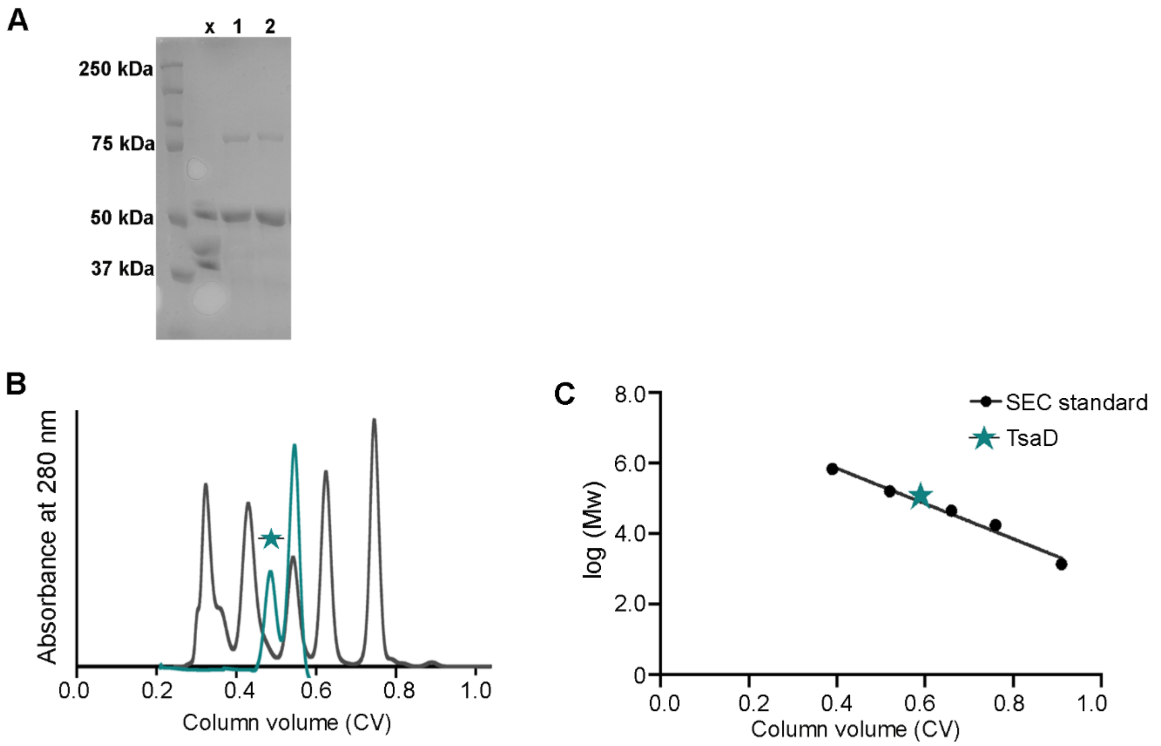

Figure S20. TsaD can be recombinantly expressed and purified in the laboratory. (**A**) SDS-PAGE reveals that tag-cleaved TsaD can be successfully isolated. The molecular mass of TsaD is approximately 51 kDa and was judged by comparison to the protein standard that is present in the far-left lane of the gel. (**B**) Once produced, the oligomeric state of TsaD was investigated using gel filtration chromatography. This experiment revealed that TsaD behaves as a homodimer in solution, which is consistent with the typically observed dimeric or tetrameric architecture of other annotated aldehyde dehydrogenases (14). (**C**) The molecular weight of TsaD was estimated using a comparison to a gel filtration standard (plotted here linearly and in panel B as a chromatogram). The standards in panel B and the data in panel C are also present in Figure S1B-C but are reproduced here for clarity and comparison to panel B.

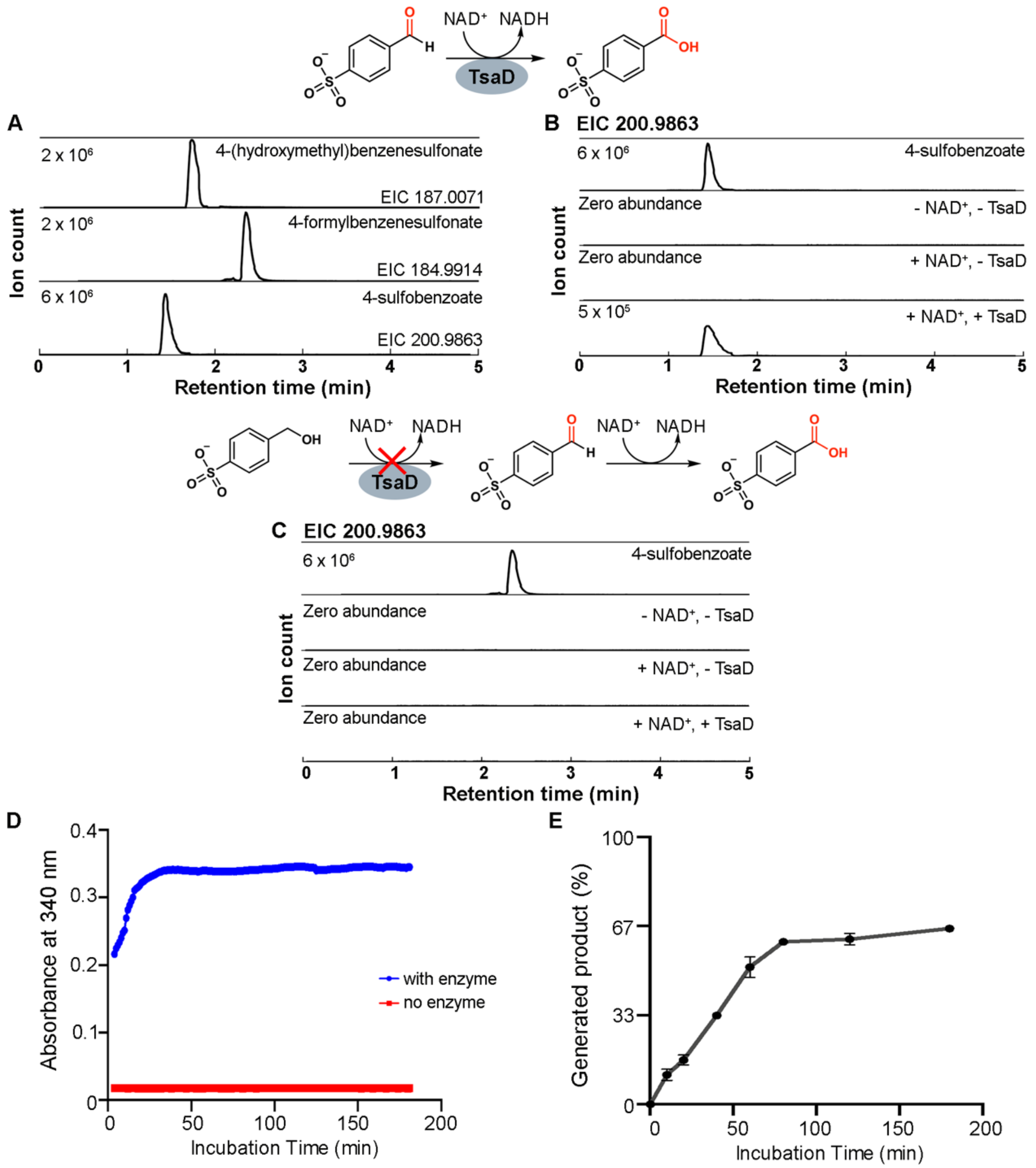

Figure S21. TsaD transforms 4-formylbenzenesulfonate into 4-sulfobenzoate. (**A**) Commercially purchased substrate and product standards were used to evaluate the enzymatic activity of TsaD. These standards could be resolved using LC-MS. (**B**) TsaD produces 4-sulfobenzoate when incubated with a 4-formylbenzenesulfonate substrate. This transformation requires NAD^+. (**C**) In contrast to that observed with TsaC, when TsaD is incubated with a 4-(hydroxymethyl)benzenesulfonate substrate and NAD^+, production of 4-sulfobenzoate is not observed. (**D**) The absorbance at 340 nm was monitored to verify that NADH is produced in the TsaD-catalyzed conversion of 4-formylbenzenesulfonate into 4-sulfobenzoate. (**E**) The extracted ion chromatogram for the product formed by TsaD with 4-formylbenzenesulfonate revealed that the optimal incubation time is 2 h for TTN experiments. The data in this panel were measured using n = 3 independent experiments and are represented as the mean value of those measurements. The traces in panel (A) are the same as trances in Figure S12A and are reproduced here for clarity.

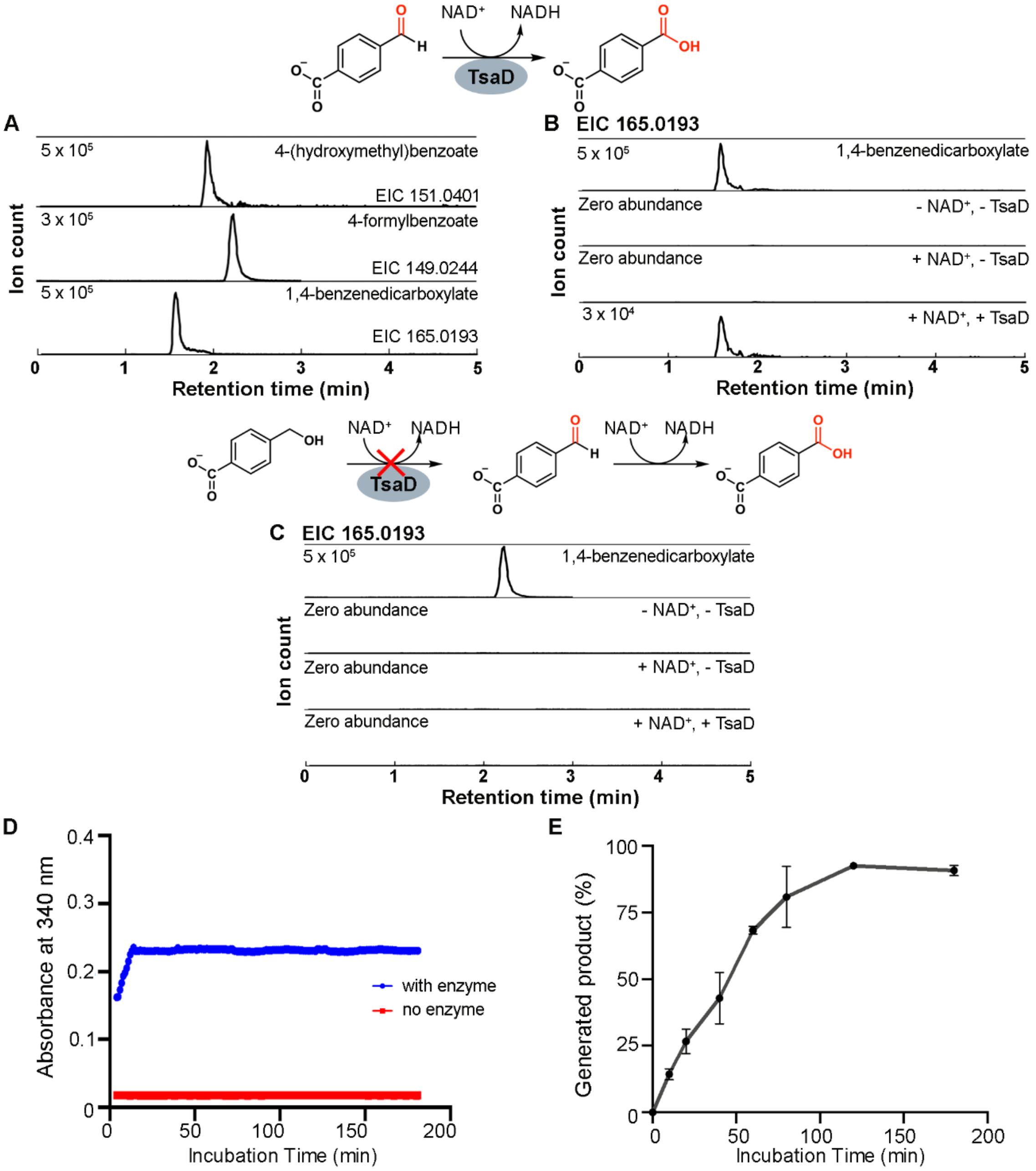

Figure S22. TsaD transforms 4-formylbenzoate into 1,4-benzenedicarboxylate. (**A**) Commercially purchased substrate and product standards were used to evaluate the enzymatic activity of TsaD. (**B**) TsaD produces 1,4-benzenedicarboxylate when incubated with a 4-formylbenzoate substrate and NAD^+. (**C**) In contrast to that observed with TsaC, when TsaD is incubated with a 4-(hydroxymethyl)benzoate substrate and NAD^+, production of 4-formylbenzoate is not observed. (**D**) The absorbance at 340 nm was monitored to verify that NADH is produced in the TsaD-catalyzed conversion of 4-formylbenzoate into 1,4-benzenedicarboxylate. (**E**) The extracted ion chromatogram for the product formed by TsaD with 4-formylbenzoate revealed that the optimal incubation time is 2 h for measuring TTNs. The data in this panel were measured using n = 3 independent experiments and are represented as the mean value of those measurements. The traces in panel (A) are the same as trances in Figure S13A and are reproduced here for clarity.

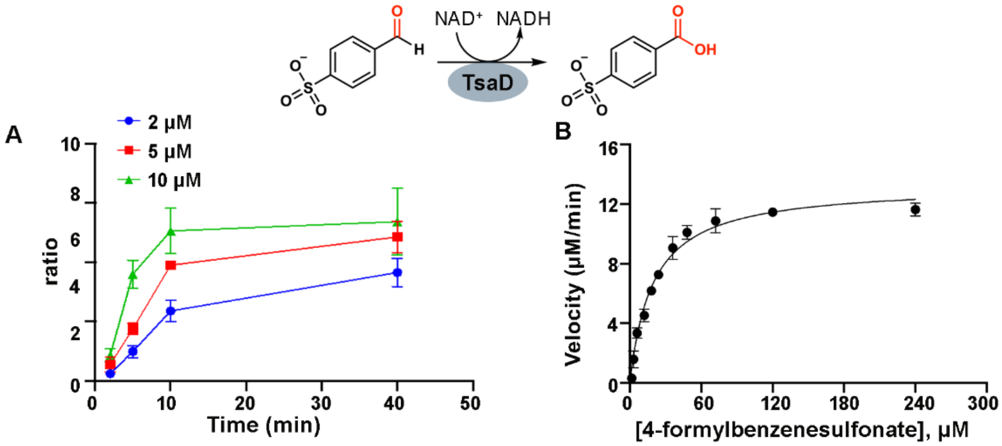

Figure S23. The steady state kinetic behavior of TsaD with a 4-formylbenzenesulfonate substrate. (**A**) The linear range of 4-sulfobenzoate formation was investigated and it was determined that kinetics should be measured using a time of 5 min with 2 μM of TsaD. (**B**) Fitting of the measured data to the Michaelis-Menten equation revealed the kinetic parameters for the TsaD-catalyzed reaction on a 4-formylbenzenesulfonate substrate. These parameters are included in Table 1. In this figure, the data were measured using n = 3 independent experiments and are represented as the mean value of those measurements. The R^2 value for the fits of the Michaelis-Menten to the data in panel B is 0.9722.

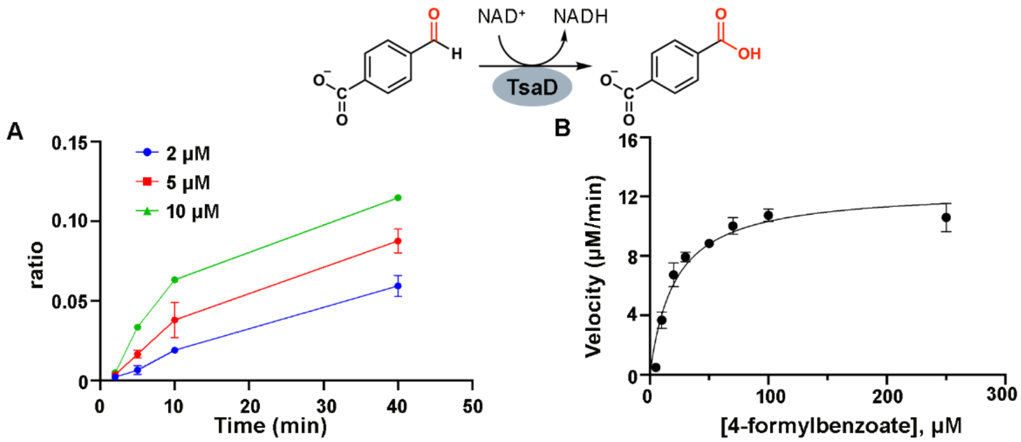

Figure S24. The steady state kinetic behavior of TsaD with a 4-formylbenzoate substrate. (**A**) The linear range of 1,4-benzenedicarboxylate formation was investigated. A time of 5 min with 2 μM of TsaD was chosen for performing the kinetic assay. (**B**) Fitting of the measured data to the Michaelis-Menten equation revealed the kinetic parameters for the TsaD-catalyzed reaction on a 4-formylbenzoate substrate (*see* Table 1). In this figure, the data were measured using n = 3 independent experiments and are represented as the mean value of those measurements. The R^2 value for the fits of the Michaelis-Menten to the data in panel B is 0.9777.

```
TsaD      MSTVLYRCPELLIGGEWRPGRHEQRLVVRNPATGEPLDELRLASADDLQLALQTTQQAFE    60
5X5T      MANVTYTDTQLLIDGEWVDAASGKTIDVVNPATGKPIGRVAHAGIADLDRALAAAQSGFE    60
          *:.* *   :***.***  .   : : * *****:*:..:  *.  **: ** ::*..**

TsaD      HWRQVPAHERCARLERGVARLRENTERIAHLLTLEQGKTLAEARMECAMAADLIKWYAEE    120
5X5T      AWRKVPAHERAATMRKAAALVRERADAIAQLMTQEQGKPLTEARVEVLSAADIIEWFADE    120
           **:******.* :.:..* :**.:: **:*:* **** *:***:*   ***:*:*:*:*

TsaD      ARRVYGRVIPARLPNSRMEVFKFPVGPVAAFSPWNFPLVLSARKLGGAIAAGCSIVLKAA    180
5X5T      GRRVYGRIVPPRNLGAQQTVVKEPVGPVAAFTPWNFPVNQVVRKLSAALATGCSFLVKAP    180
          .******::* *  .::  *.* ********:*****:   .***..*:*:***:::**

TsaD      EETPASVAAMVDC-LNQELPPGVVQLLYGVPAEVSQALIASPVVRKVTFTGSVPVGRHLA    239
5X5T      EETPASPAALLRAFVDAGVPAGVIGLVYGDPAEISSYLIPHPVIRKVTFTGSTPVGKQLA    240
          ****** **:: . ::  :* **: *:** ***:*. **  **:********.***::**

TsaD      ELSARHLKRITLELGGHAPVIVCGDADIARTVNLMVQHKFRNAGQACLAPTRFFVDRRIY    299
5X5T      SLAGLHMKRATMELGGHAPVIVAEDADVALAVKAAGGAKFRNAGQVCISPTRFLVHNSIR    300
          .*:. *:** *:**********. ***:* :*:     *******.*::****:*.. *

TsaD      GDFVDAFG-ATQALRVGAGMAAETQMGPVASARRQAAVQDLIARSVAAG--ARPVASAVP    356
5X5T      DEFTRALVKHAEGLKVGNGLEEGTTLGALANPRRLTAMASVIDNARKVGASIETGGERIG    360
          .:*. *:   ::.*:** *:   * :* :*. ** :*: .:* .:  .*   .  .. :

TsaD      EAGYFVAPTLLADVPLDAPVMSEEPFGPVACAVPFDSLDQAIAQANHNPYGLAGYLFTDS    416
5X5T      SEGNFFAPTVIANVPLDADVFNNEPFGPVAAIRGFDKLEEAIAEANRLPFGLAGYAFTRS    420
          . * *.***::*:***** *:.:*******.   **.*::***:**: *:***** ** *

TsaD      AKAILAVSERLEVGSLAVNGMGVSVPEAPFGGVKDSGYGSESGTEGMEAFLDTKFMHYVA    476
5X5T      FANVHLLTQRLEVGMLWINQPATPWPEMPFGGVKDSGYGSEGGPEALEPYLVTKSVTVMA    480
             :  :::***** * :*  ..  ** *************.* *.:* :* ** :  :*

TsaD      -     476
5X5T      V     481
```

Figure S25. A sequence alignment of TsaD with α-ketoglutarate-semialdehyde dehydrogenase from *Azospirillum basilense* (PDB:5X5T (15)) was used to identify the catalytically important Cys residue (pink) involved in forming the carboxylic acid products of TsaD.

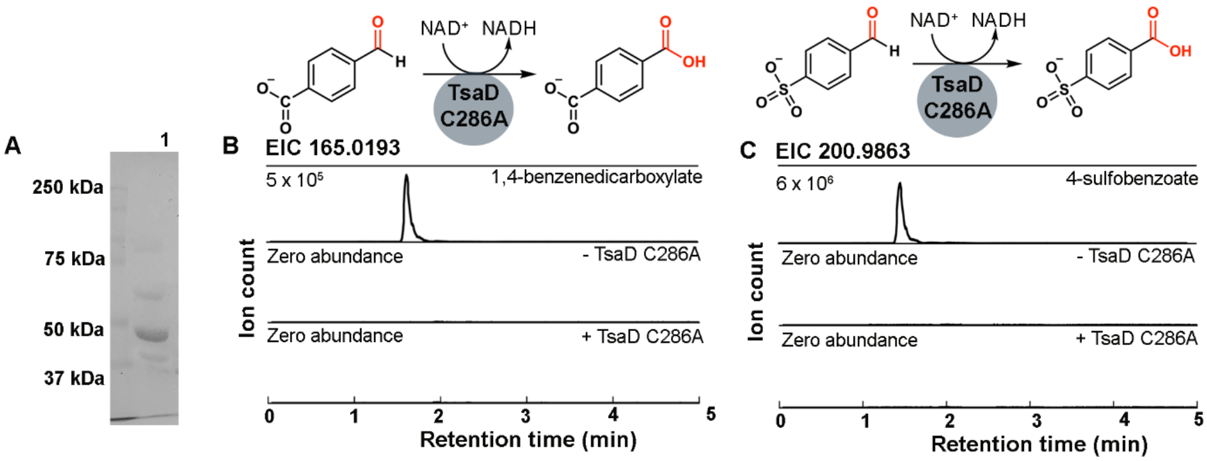

Figure S26. A C286A variant of TsaD is not able to oxidize either 4-formylbenzenesulfonate or 4-formylbenzoate. (**A**) SDS-PAGE reveals that the C286A TsaD variant can be recombinantly expressed and purified using affinity chromatography. (**B**) The combination of the C286A variant of TsaD with NAD^+ and 4-(hydroxymethyl)benzoate does not result in formation of 1,4-benzenedicarboxylate. (**C**) Incubation of the C286A TsaD variant with NAD^+ and 4-formylbenzenesulfonate does not result in formation of 4-sufobenzoate.

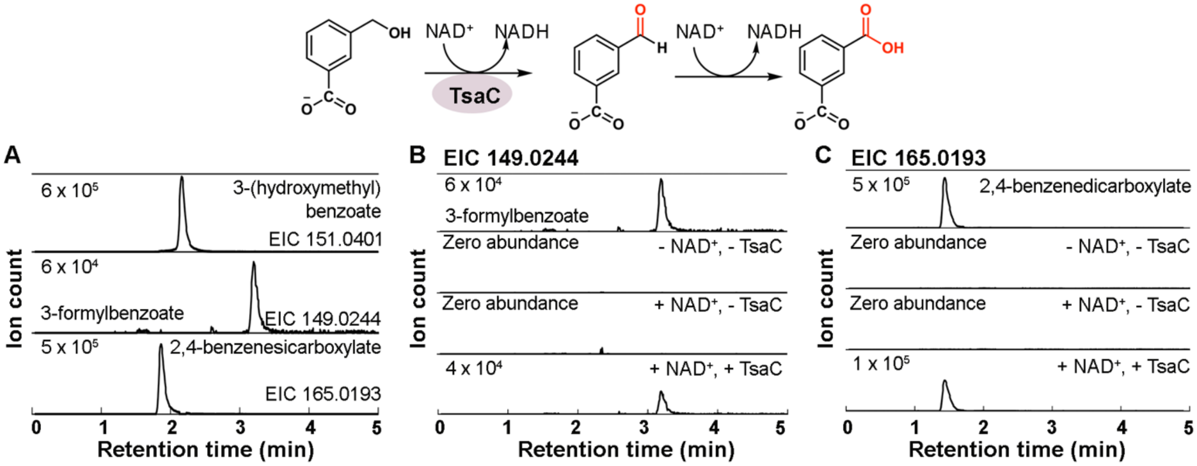

Figure S27. TsaC performs chemistry on a 3-(hydroxymethyl)benzoate substrate. (**A**) Commercially purchased substrate and product standards were used to evaluate the enzymatic activity of TsaC on 3-(hydroxymethyl)benzoate. (**B**) TsaC produces 3-formylbenzoate when incubated with a 3-(hydroxymethyl)benzoate substrate. This transformation is dependent on the presence of NAD^+ in the reaction mixture. (**C**) Similar to that shown in panel B, and as described for the reported native substrates (1-3), when TsaC is incubated with a 3-(hydroxymethyl)benzoate substrate and NAD^+, production of 2,4-benzenedicarboxylate is observed.

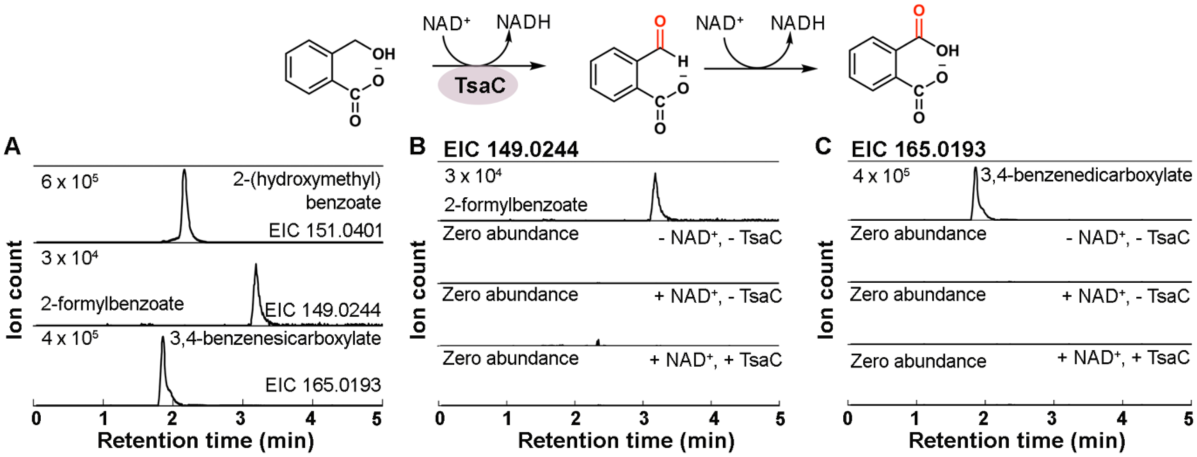

Figure S28. TsaC is unable to perform chemistry on 2-(hydroxymethyl)benzoate. (**A**) Commercially purchased substrate and product standards were used to evaluate the enzymatic activity of TsaC on 2-(hydroxymethyl)benzoate. (**B**) TsaC does not oxidize 2-(hydroxymethyl)benzoate into the corresponding aldehyde product. (**C**) Similarly, when TsaC is incubated with a 2-(hydroxymethyl)benzoate substrate and NAD^+, no formation of 3,4-benzenedicarboxylate is detected.

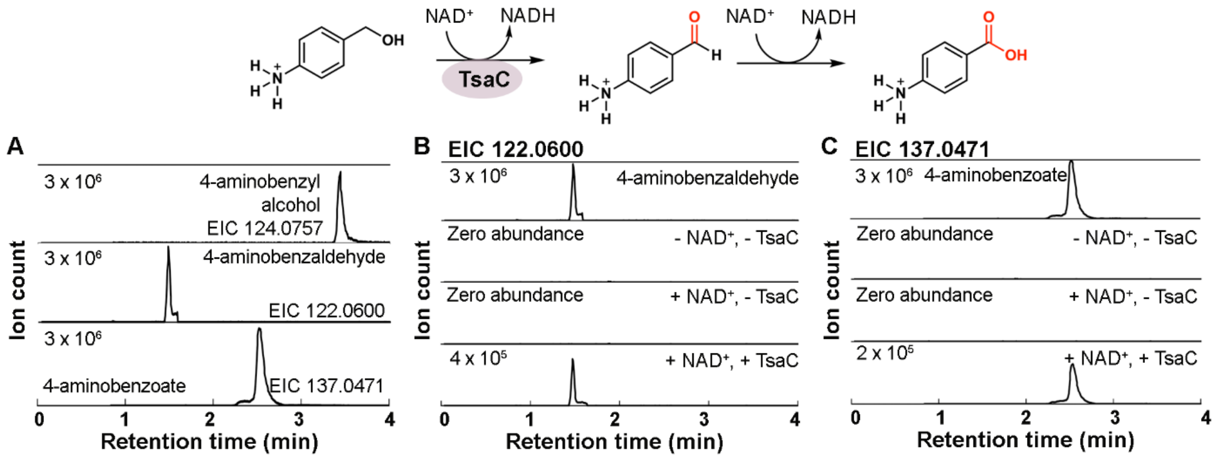

Figure S29. TsaC can perform chemistry on substrates like 4-aminobenzyl alcohol that have different functional groups at C1. (**A**) Commercially purchased substrate and product standards were used to evaluate the enzymatic activity of TsaC on 4-aminobenzyl alcohol. (**B**) TsaC produces a 4-aminobenzaldehyde product when provided with a 4-aminobenzyl alcohol substrate and NAD^+. (**C**) TsaC also produces another oxidized product, 4-aminobenzoate, when provided with a 4-aminobenzyl alcohol substrate and NAD^+.

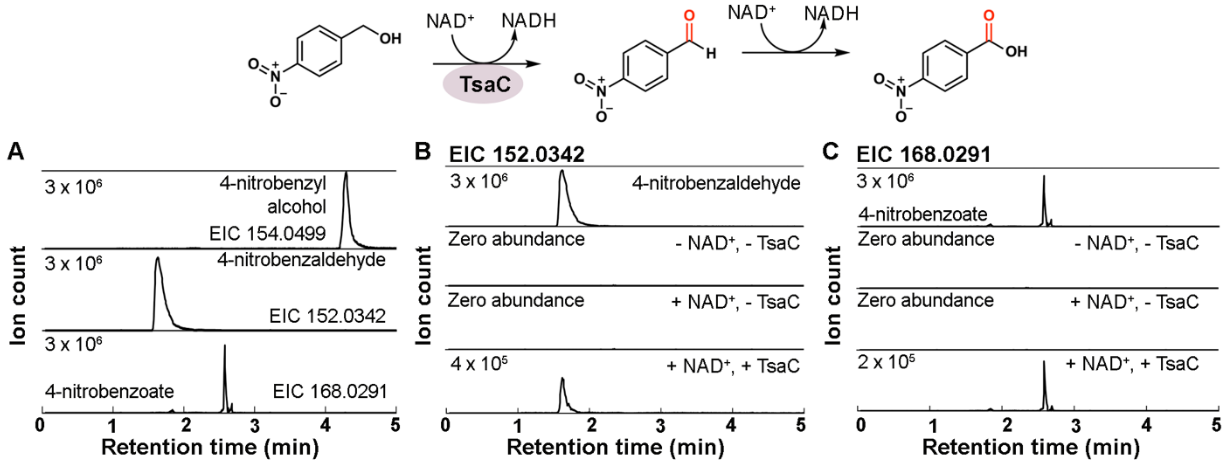

Figure S30. TsaC shows activity when provided with a 4-nitrobenzyl alcohol substrate, which has a different functional group at C1 than the reported native substrates (1-3). (**A**) Commercially purchased substrate and product standards were used to evaluate the enzymatic activity of TsaC 4-nitrobenzyl alcohol. (**B**) TsaC produces a 4-nitrobenzaldehyde product when provided with a 4-nitrobenzyl alcohol substrate and NAD^+. (**C**) TsaC also produces a 4-nitrobenzoate product when provided with a 4-nitrobenzyl alcohol substrate and NAD^+.

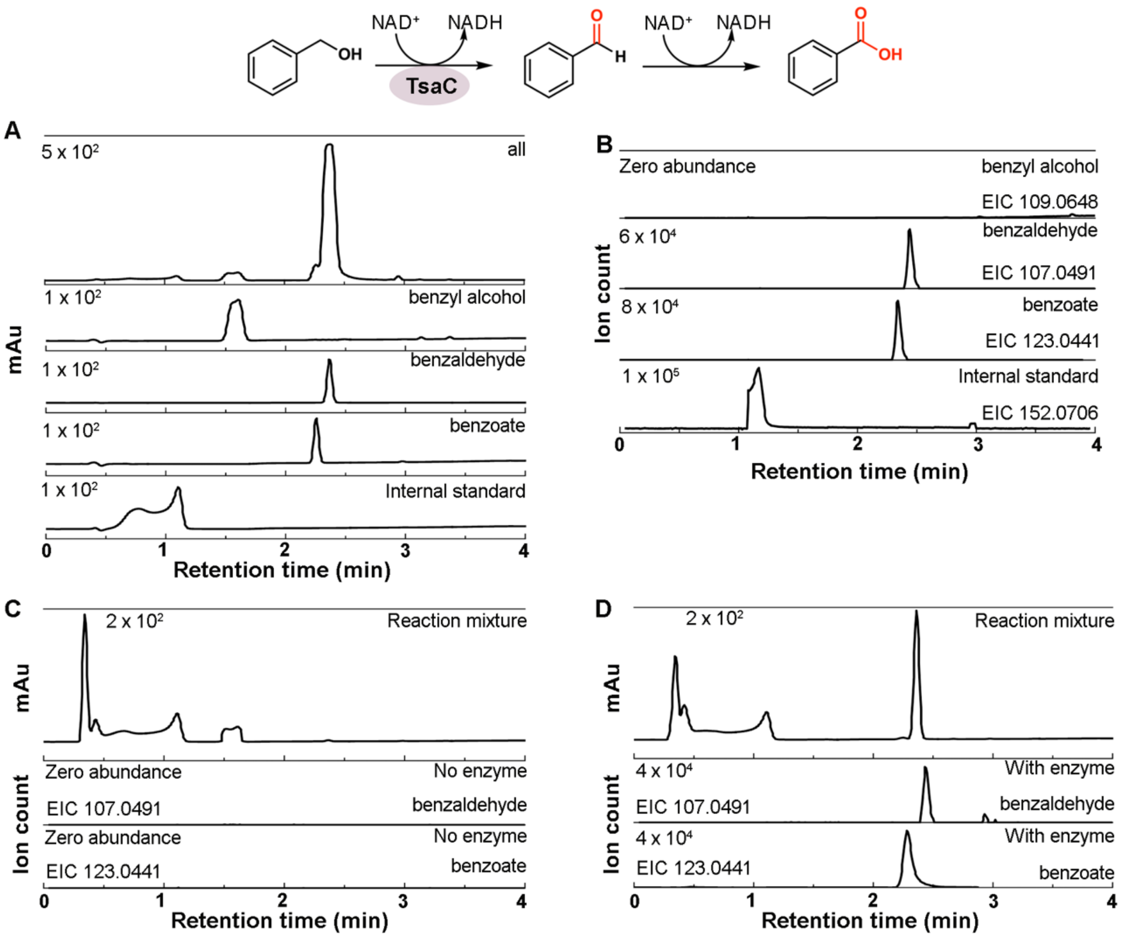

Figure S31. TsaC performs chemistry on a benzyl alcohol substrate. (**A**) Commercially purchased substrate and product standards were used to evaluate the enzymatic activity of TsaC on benzyl alcohol. For these experiments, the low ionizability of benzyl alcohol meant that its presence needed to be monitored using a diode array detector to detect its formation at 260 nm. (**B**) The benzaldehyde and benzoate molecules, on the other hand, could be ionized and detected similarly to the other compounds analyzed in this work. (**C**) In the absence of TsaC, no benzaldehyde or benzoate products were able to be detected. However, the UV-Vis absorbance peak around 1.5 min at 260 nm indicates the presence of benzyl alcohol in the reaction mixture. (**D**) LC-MS revealed that the combination of TsaC with benzyl alcohol results in formation of aldehyde (benzaldehyde, m/z = 107.0491) and carboxylic acid (4-isopropylbenzoate, m/z = 123.0441) products. In this experiment, as indicated by the loss of a UV-Vis absorbance peak at 1.5 min, benzyl alcohol is consumed during the TsaC-catalyzed reaction.

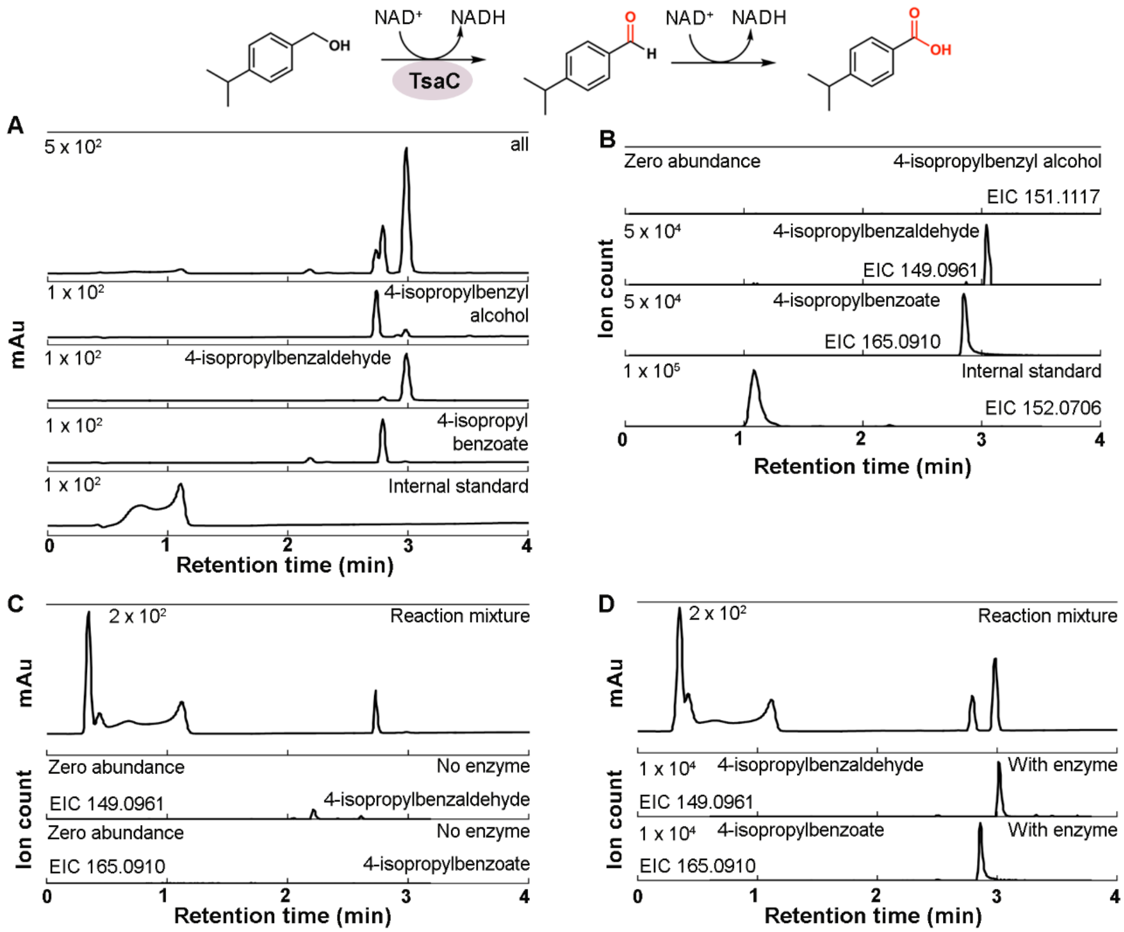

Figure S32. TsaC transforms a 4-isopropylbenzyl alcohol substrate into the corresponding aldehyde and carboxylic acid products. (**A**) Commercially purchased substrate and product standards were used to evaluate the enzymatic activity of TsaC on 4-isopropylbenzyl alcohol. For these experiments, as described for benzyl alcohol, the low ionizability of 4-isopropylbenzyl alcohol meant that its presence needed to be monitored using a diode array detector to identify peaks at 260 nm. (**B**) The 4-isopropylbenzaldehyde and 4-isopropylbenzoate molecules, on the other hand, could be ionized and detected and thus were not analyzed using the diode array detector. (**C**) When TsaC is not present in the reaction, 4-isopropylbenzaldehyde and 4-isopropylbenzoate are not formed. (**D**) LC-MS revealed that the combination of TsaC with 4-isopropylbenzyl alcohol results in formation of 4-isopropylbenzaldehyde (m/z = 149.0961) and 4-isopropylbenzoate (m/z = 165.0910). The lack of a UV-Vis absorbance peak around 2.5 min indicates that the 4-isopropylbenzyl alcohol substrate is consumed during the TsaC-catalyzed reaction.

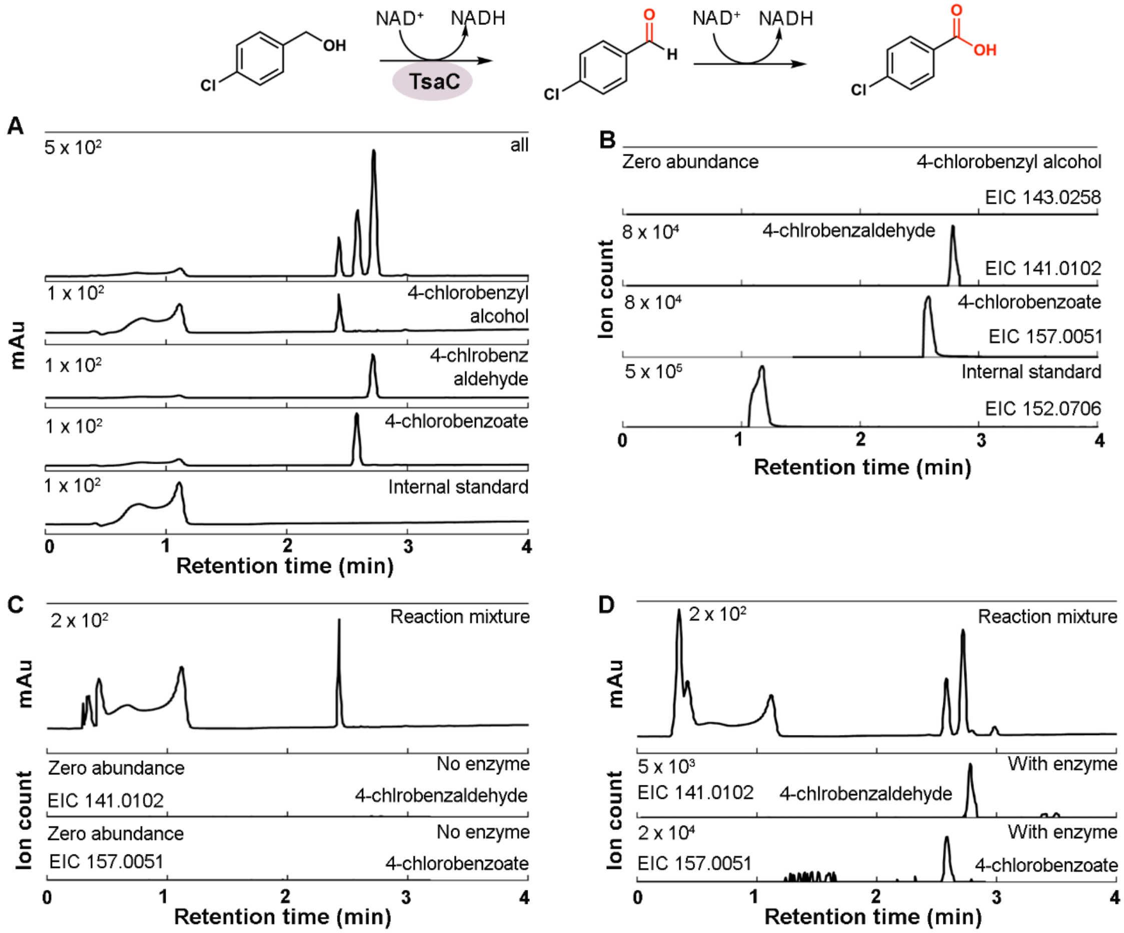

Figure S33. TsaC converts 4-chlorobenzyl alcohol into 4-chlorobenzaldehyde and 4-chlorobenzoate products. (**A**) Commercially purchased substrate and product standards were used to evaluate the enzymatic activity of TsaC on 4-chlorobenzyl alcohol. In these experiments, the presence of 4-chlorobenzyl alcohol in the reaction mixtures was monitored using a diode array detector to identify peaks at 260 nm. (**B**) The 4-chlorobenzaldehyde and 4-chlorobenzoate molecules were ionized and detected. (**C**) For a control experiment, it was determined that 4-chlorobenzaldehyde or 4-chlorobenzoate are not formed in the absence of TsaC. (**D**) The combination of TsaC with 4-chlorobenzyl alcohol reveals consumption of 4-chlorobenzyl alcohol and subsequent formation of 4-chlorobenzaldehyde (m/z = 141.0102) and 4-chlorobenzoate (m/z = 157.0051).

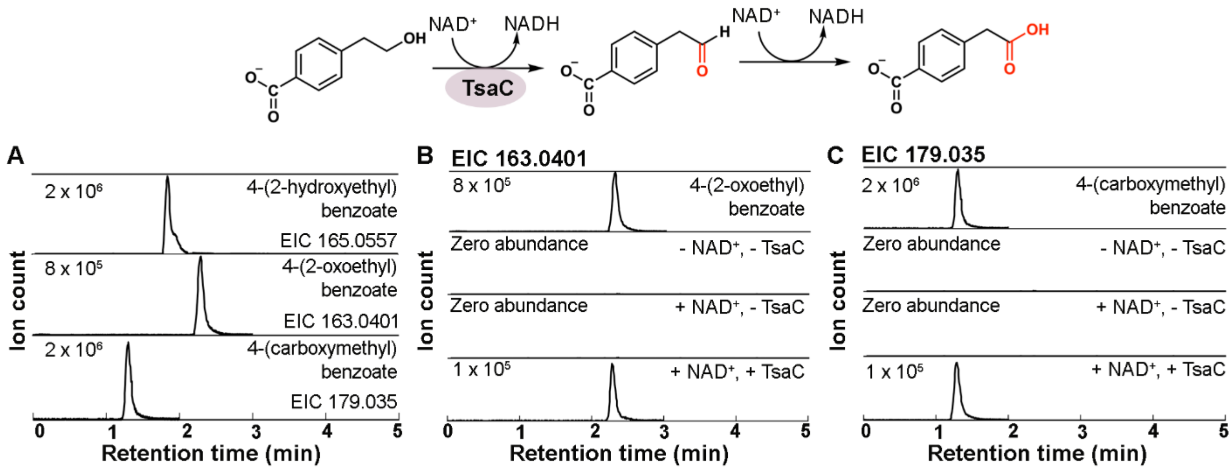

Figure S34. TsaC oxidizes a 4-(2-hydroxyethyl)benzoate substrate into aldehyde and carboxylic acid products. (**A**) Commercially purchased substrate and product standards were used to evaluate the enzymatic activity of TsaC on 4-(2-hydroxyethyl)benzoate. (**B**) TsaC produces 4-(2-oxoethyl)benzoate when provided with a 4-(2-hydroxyethyl)benzoate substrate and NAD^+. (**C**) TsaC also produces 4-(carboxymethyl)benzoate when provided with a 4-(2-hydroxyethyl)benzoate substrate and NAD^+.

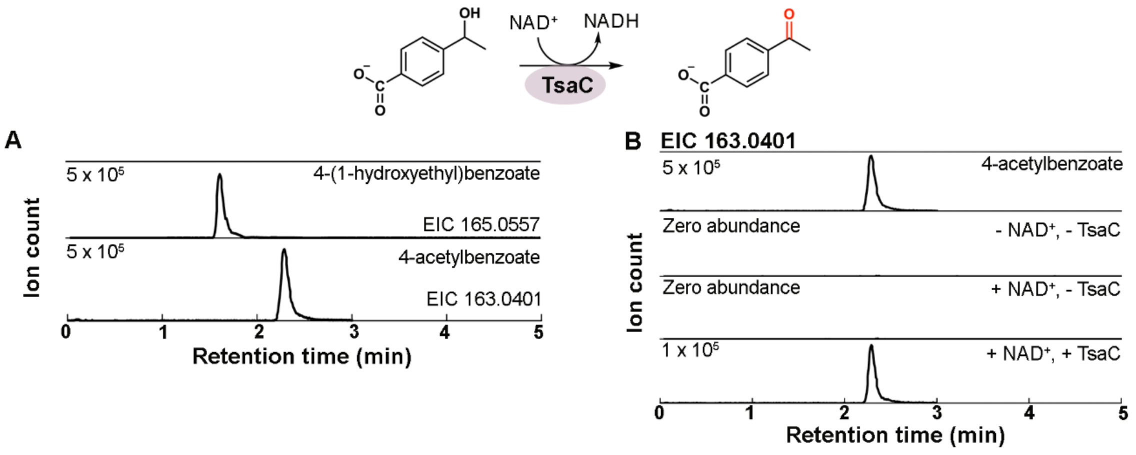

Figure S35. TsaC performs chemistry on the secondary alcohol 4-(1-hydroxyethyl)benzoate. (**A**) Commercially purchased substrate and product standards were used to evaluate the enzymatic activity of TsaC on 4-(1-hydroxyethyl)benzoate. (**B**) TsaC produces 4-acetylbenzoate when incubated with a 4-(1-hydroxyethyl)benzoate substrate in the presence of NAD^+.

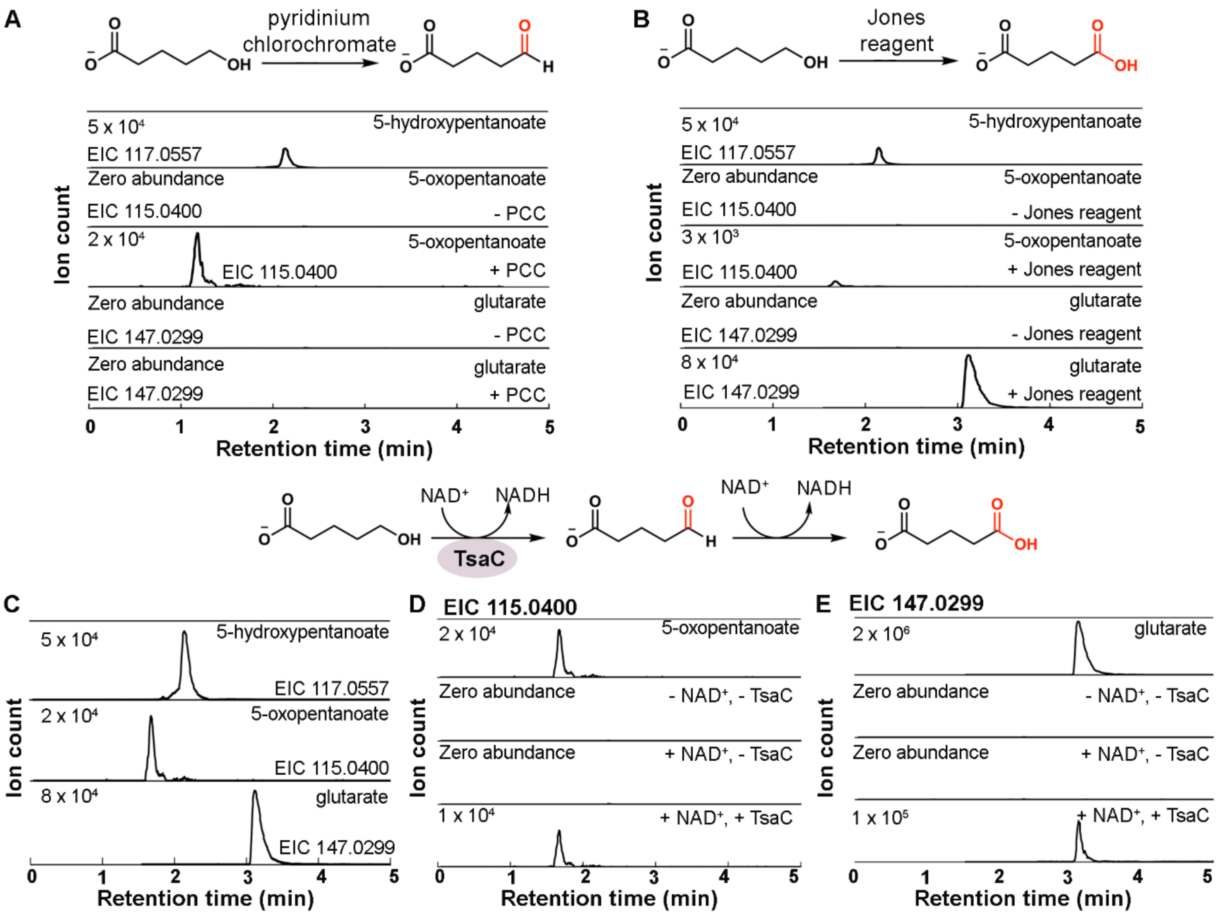

Figure S36. TsaC oxidizes the linear alcohol substrate 5-hydroxypentanoate. (**A**) A 5-oxopentanoate product standard was made synthetically. In brief, 5-hydroxypentanoate was oxidized by the addition of pyridinium chlorochromate (PCC) to produce 5-oxopentanoate. An extracted ion chromatogram indicates the successful generation of 5-oxopentanoate (m/z = 115.0400) using the implemented method. (**B**) A glutarate compound standard was also synthetically produced: 5-hydroxypentanoate was oxidized by the addition of Jones reagent (CrO_3) to produce glutarate. The extracted ion chromatogram reveals the retention time of glutarate is around 3.1 min. (**C**) LC-MS was used to separate 5-hydroxypentanoate from the expected product. (**D**) TsaC produces 5-oxopentanoate when incubated with a 5-hydroxypentanoate substrate in the presence of NAD^+. (**E**) TsaC also produces glutarate when incubated with a 5-hydroxypentanoate substrate in the presence of NAD^+.

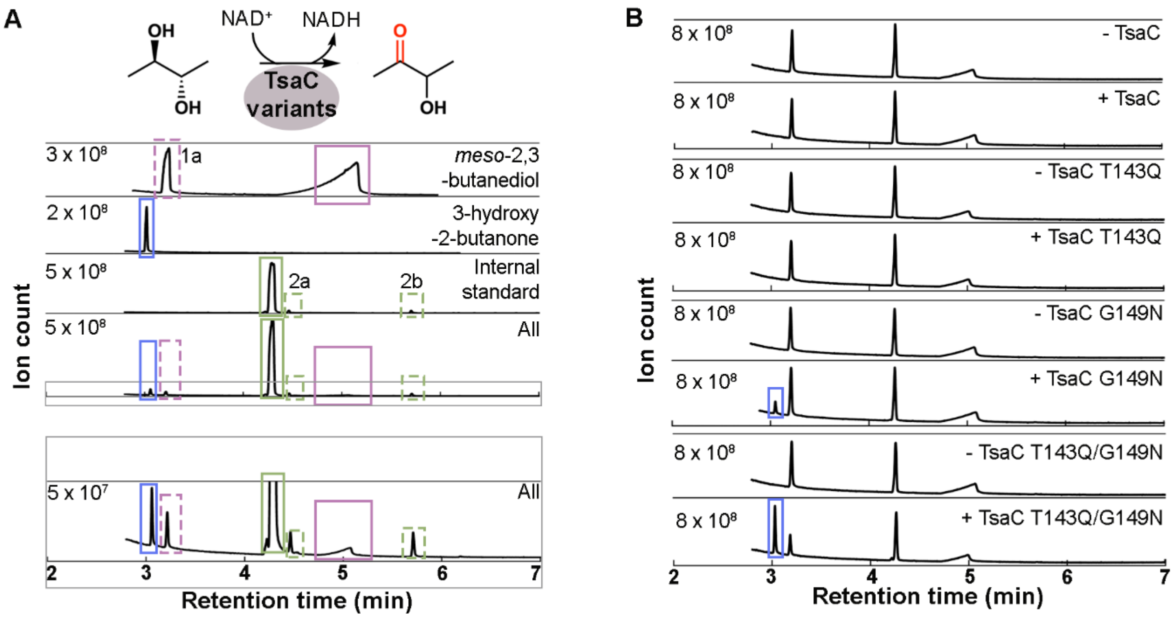

Figure S37. TsaC can be engineered to perform chemistry on a *meso*-2,3-butanediol substrate. (**A**) A GC-MS method was developed to allow for separation of *meso*-2,3-butanediol from 3-hydroxy-2-butanone and the internal *m*-xylene standard. Peak 1a is an impurity from the commercially purchased substrate standard. Peak 2a is the xylene isomer *p*-xylene and peak 3a is the oxidation product of *m*-xylene. In this panel, boxes with solid lines represent GC-MS peaks of compound standards; dashed boxes represent GC-MS peaks of impurities from the substrate and internal standards. (**B**) The ability of wild-type TsaC and TsaC variants to convert *meso*-2,3-butanediol into 3-hydroxy-2-butanone was probed. Both of the G149N and T143Q/G149N TsaC variants were able to transform *meso*-2,3-buntanediol into 3-hydroxy-2-butanone. In this panel the solid boxes highlight 3-hydroxy-2-butanone.

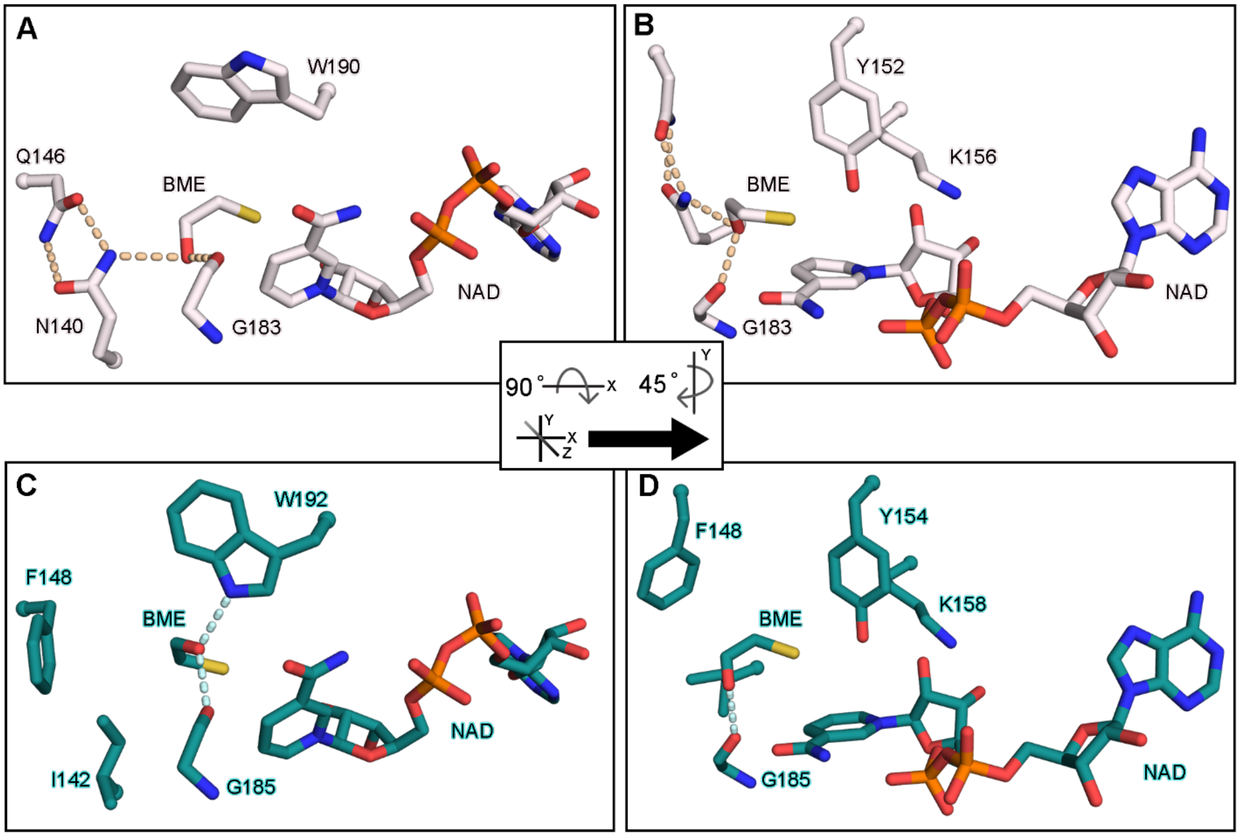

Figure S38. The residues that constitute the 'gate' near the substrate binding site have been implicated in determining the stereochemistry of accepted the substrate for BDH. The structure of *meso*-2,3-butanediol dehydrogenase (PDB:1GEG) (4) (**A** and **B**) contrasts with the L-2,3-butanediol dehydrogenase structure (PDB:3A28) (16) (**C** and **D**), where the gating residues Gln146 and Asn140 are instead Phe148 and Ile142. Shifting the angle of viewing, BME can be clearly observed to be oriented over NAD^+ such that it is in proximity to the catalytic Tyr residue, which itself is in proximity to the catalytic Lys residue.

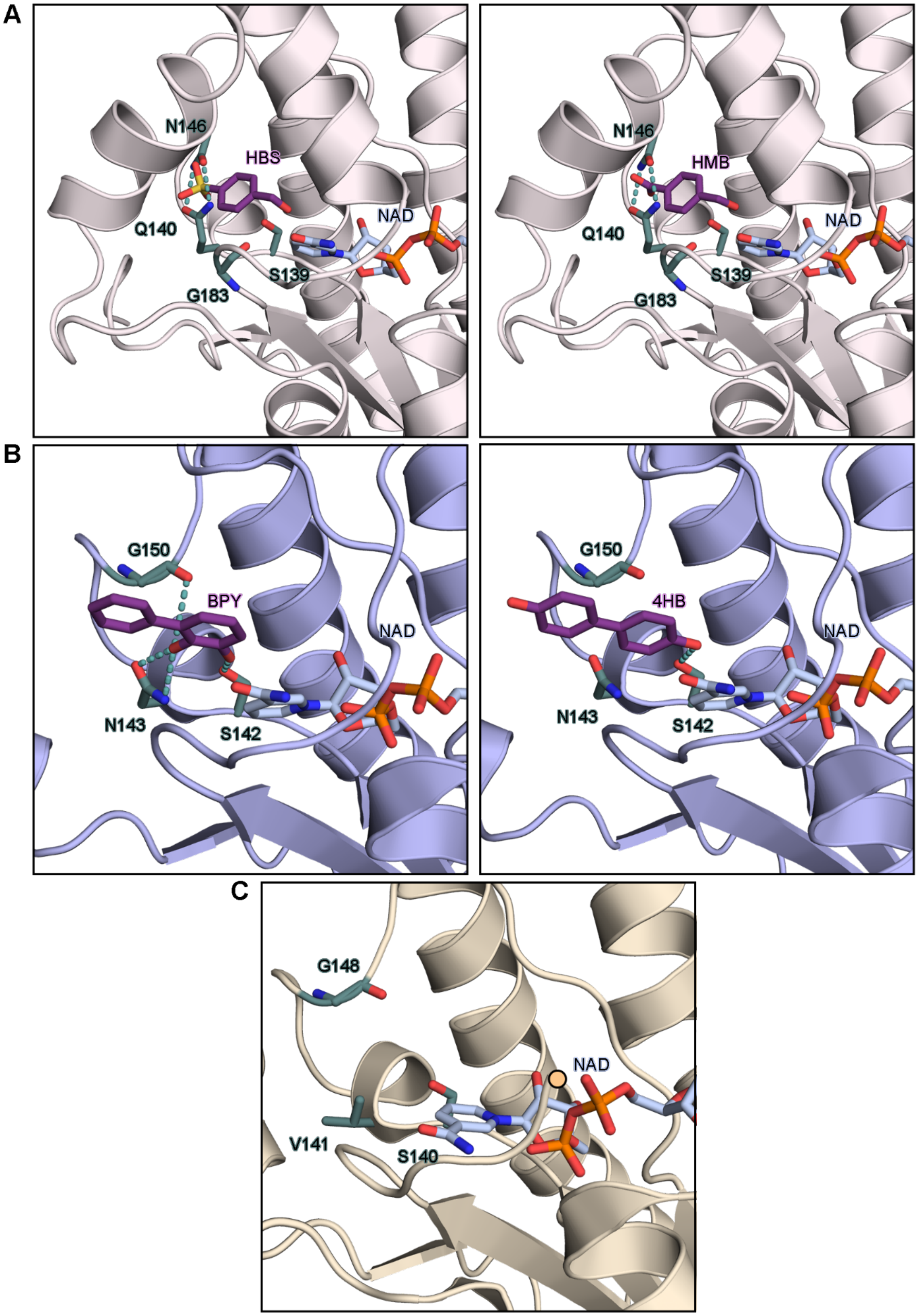

A
N146
HBS
Q140
NAD
S139
G183
N146
HMB
Q140
NAD
S139
G183
B
G150
BPY
NAD
N143
S142
G150
4HB
NAD
N143
S142
C
G148
NAD
V141
S140

D

```
TsaC      MNLNKQVAIVTGGASGFGAAIARRLSQAGAAVLVADLNAEGAQRMATELNAAGGRALGMA     60
BDH       ---MKKVALVTGAGQGIGKAIALRLVKDGFAVAIADYNDATAKAVASEINQAGGHAVAVK     57
NahB      -MGNQQVVSITGAGSGIGLELVRSFKLAGYCVSALVRNEEQEAL---LCNEFKDALEIVV     56
BphB      MKLTGEVALITGGASGLGRALVDRFVAEGARVAVLDKSAERLRE---LEVAHGGNAVGVV     57
               :*. :**...*:*  :.  :   *  *     .               .    :

TsaC      CDVSKEADYRAVVDAAIAQLGGLHIVVNNAGTTHRNKPALAVT----EDEFDRVYRVNLK     116
BDH       VDVSDRDQVFAAVEQARKTLGGFDVIVNNAGVAP-STPIESIT----PEIVDKVYNINVK     112
NahB      GDVRDHATNEKLIKQTIDRFGHLDCFIANAGIWDYMLNIE-EPWEKISSSFDEIFDINVK     115
BphB      GDVRSLQDQKRAAERCLAAFGKIDTLIPNAGIWDYSTALADLPEDKIDAAFDDIFHVNVK     117
           ** .        .     :* :. .: ***                   .* :: :*:*

TsaC      SVYWSAQCALPHFAQQGHGV-MVNVASTTGVRPGPGLTWYSGSKAAMINLTKGLALEFAR     175
BDH       GVIWGIQAAVEAFKKEGHGGKIINACSQAGHVGNPELAVYSSSKFAVRGLTQTAARDLAP     172
NahB      SYFSGISAALPELKKTNG--SVVMTASVSSHAVGGGGSCYIASKHAVLGMVKALAYELAP     173
BphB      GYIHAVKACLPALVSSRG--SVVFTISNAGFYPNGGGPLYTATKHAVVGLVRQMAFELAP     175
          .   . ...:  : .      :: . * :.   .     * .:* *: .:.:  * ::*

TsaC      SGVRINAVNPMIGETPMMADFMGMEDT------PANRERFLSRIPLGRFTRPDDVASAVA     229
BDH       LGITVNGYCPGIVKTPMWAEIDRQVSEAAGKPLGYGTAEFAKRITLGRLSEPEDVAACVS     232
NahB      -EIRVNAVSPGGTVTSLCGPASAGFDKMHMKDMPGIDDMIKGLTPLGFAAKPEDVVAPYL     232
BphB      -HVRVNGVAPGGMNTDLRGPSSLGLSEQSISSVP-LADMLKSVLPIGRMPALEEYTGAYV     233
            : :*.  *    * : .      .             :     :*     :: ..

TsaC      FLAS-DDASFLTGVCLDVDGGRNI------------------------ 252
BDH       YLAS-PDSDYMTGQSLLIDGGMVFN----------------------- 256
NahB      LLASRKQGKFITGTVISIDGGMALGRK--------------------- 259
BphB      FFATRGDSLPATGALLNYDGGMGVRGFLTAAGGADLPEKLNINREGQE 281
```

Figure S39. The residues in the gating position appear to serve a conserved role in substrate recognition. (**A**) Modeling the TsaC substrates into the structure of BDH (PDB:1GEG) (4) highlights the clash between the substrate and the gating residues Ans146 and Gln140. These residues shelter the considerably smaller BDH substrate and provide hydrogen bonding to position the substrate. The substrates were docked to orient the methoxy bond over the sulfur-carbon bond of the BME in the BDH structure. The left panel of B is the same as that shown in Figure 5B, but is repeated here for clarity and comparison. (**B**) Crystal structures of biphenyl dehydrogenase (BphB, PDB:1BDB) (17) with products bound again support the conclusion that the ‘gating’ residues recognize and provide space for the substrate to bind. (**C**) A structure of *cis*-dihydrodiol naphthalene dehydrogenase (NahB, PBD: 5XTF) (18) again showcases a space-opening Gly residue and a semi-bulky residue (Val) in the substrate binding gate. In this figure, an orange circle denotes the start of the unmodeled substrate binding loop. (**D**) A sequence alignment of TsaC, BDH, BphB, and NahB illustrates the important gating residue positions (yellow), as well as three of the catalytic tetrad residues (purple). Even though the BphB gating Gly residue does not perfectly align in sequence, it does superimpose in the structures on the Gly of TsaC and NahB. HBS = 4-(hydroxymethyl)benzenesulfonate; HMB = 4-(hydroxymethyl)benzoate; BPY = 2,3-dihydroxybiphenyl; 4HB = 4,4′ -dihydroxybiphenyl.

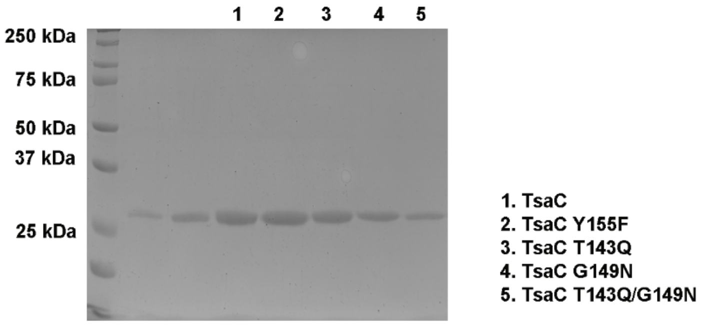

Figure S40. The purity of TsaC and its variants were assessed using SDS-PAGE. TsaC has a molecular weight of approximately 27 kDa and its presence was verified by comparison to a protein standard.

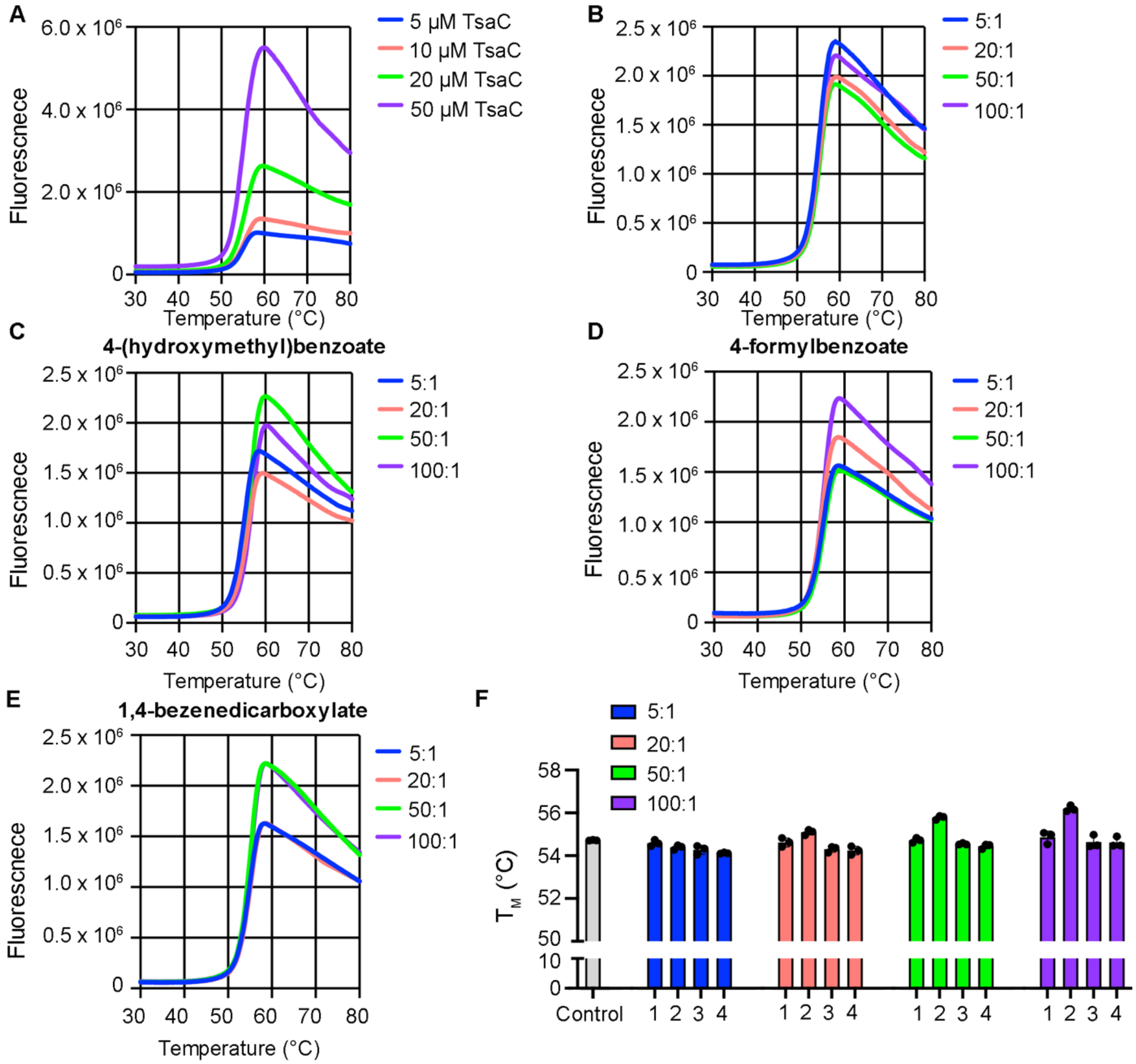

Figure S41. Wild-type TsaC with NAD^+, alcohol (4-(hydroxymethy)benzoate), aldehyde (4-formylbenzoate) and acid (1,4-benzenedicarboxylate) were assayed and analyzed using differential scanning fluorimetry. Thermal denaturation curves are organized by reaction mixture type. (**A**) Different concentrations of TsaC have similar melting curves, suggesting similar thermal stability. A concentration of 10 µM TsaC was chosen as the optimal concentration for the assays. (**B**) The thermal denaturation curve of different ratios of NAD^+ to TsaC suggested that NAD^+ minimally influences enzyme thermal stability. (**C**) The thermal denaturation curve of different ratios of NAD^+ to TsaC with the addition of excess alcohol substrate (4-(hydroxymethyl)benzoate) indicates increased stability in the presence of the alcohol substrate. (**D**) In the presence of excess aldehyde (4-formylbenzoate), with different ratios of NAD^+ and TsaC, the thermal stability of TsaC is not altered. (**E**) Similarly, the thermal stability of TsaC with different ratios of NAD^+ in the presence of acid (1,4-benzenedicarboxylate) is not altered. (**F**) The bar chart shows the melting points of TsaC with NAD^+, alcohol, aldehyde and acid compounds. On the x-axis, 1 is no compound addition, 2 is addition of alcohol, 3 is addition of aldehyde, and 4 is addition of acid. In all panels, the data were measured using n = 3 independent experiments. Indicated ratios represent the concentration of NAD^+ added to TsaC. In panels (A) to (E), the data are presented as the mean values of these measurements. In panel (F), the data are presented as the mean value ± SD of these measurements.

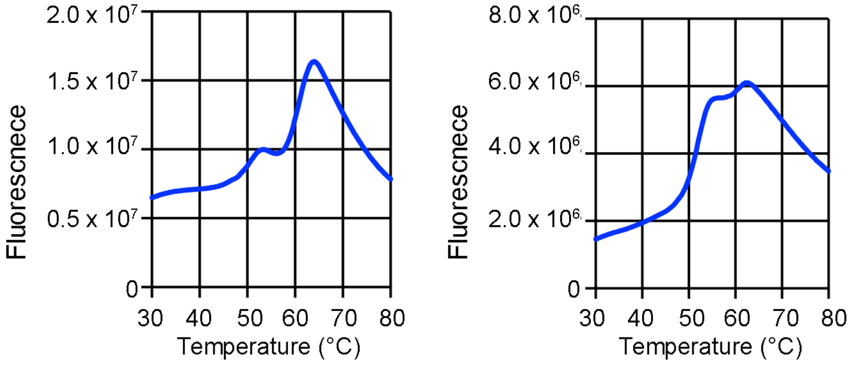

Figure S42. The thermal stabilities of wild-type and Y155F TsaC decrease substantially at a high protein concentration. TsaC (Left) and Y155F TsaC (right) exhibit a lower T_m at a concentration of 500 μM, indicating decreased thermal stability due potentially to the reversible formation of higher-order oligomeric states at this concentration. Here, the T_m values are 49.0 ± 0.5 °C and 47.3 ± 0.5 °C for wild-type and Y155F TsaC, respectively.

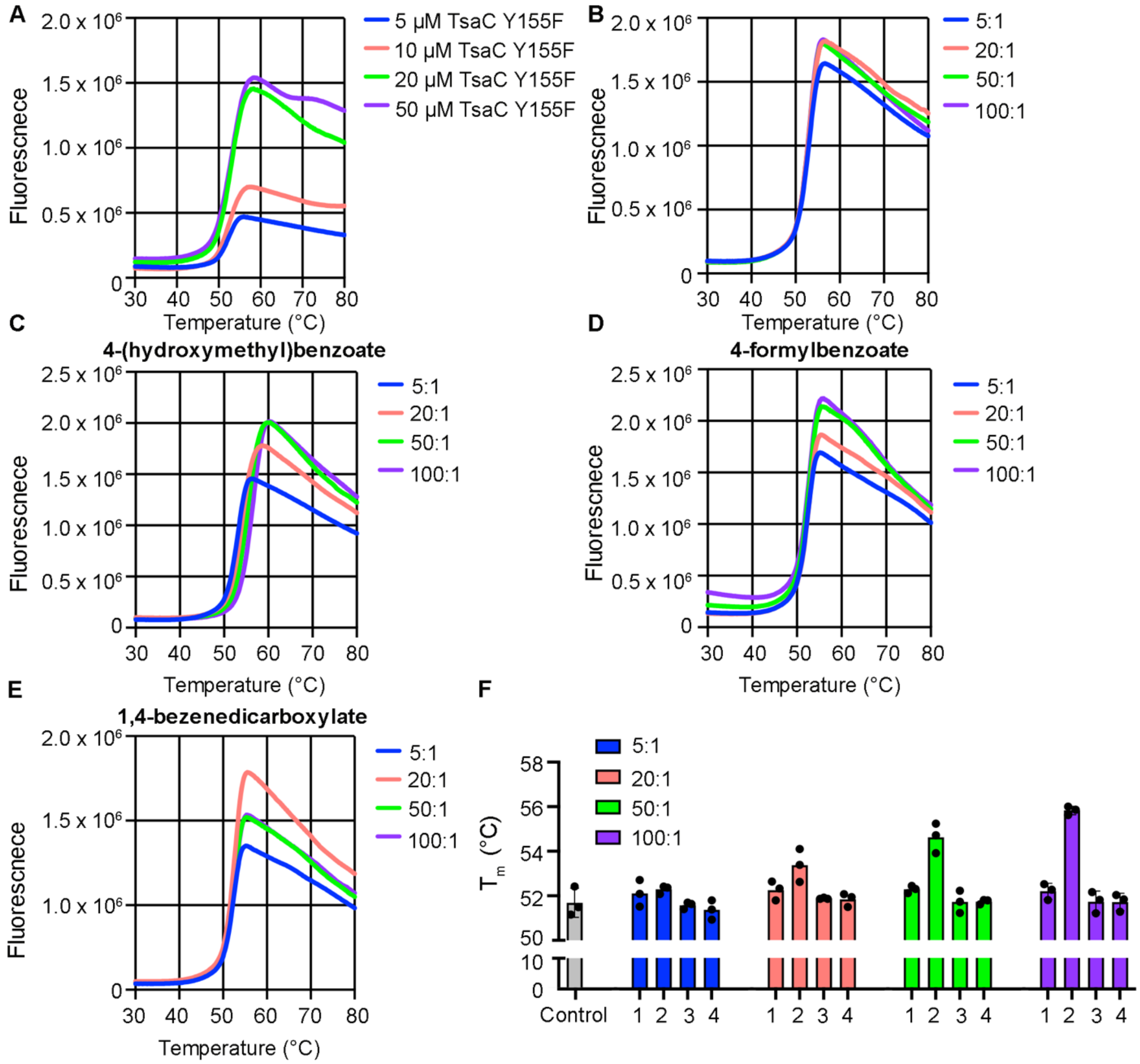

Figure S43. The thermal stability of Y155F TsaC with NAD$^+$, 4-(hydroxymethy)benzoate, 4-formylbenzoate, and 1,4-benzenedicarboxylate were assayed and analyzed using differential scanning fluorimetry. (**A**) Different concentrations of Y155F TsaC have similar melting curves, indicating their similar thermal stability. A concentration of 10 μM Y155F TsaC was chosen as the optimal concentration to pursue the subsequent experiments. (**B**) The thermal denaturation curves of different ratios of NAD$^+$ to Y155F TsaC suggested that NAD$^+$ minimally influences enzyme stability. (**C**) The thermal stability of different ratios of NAD$^+$ to Y155F TsaC with the addition of 4-(hydroxymethyl)benzoate was probed. The thermal stability of enzymes was increased in the presence of 4-(hydroxymethyl)benzoate and NAD$^+$. (**D**) The thermal denaturation curve of different ratios of NAD$^+$ to TsaC Y155F in the presence of 4-formylbezoate suggest that enzyme stability is not affected. (**E**) Similarly, the thermal stability of Y155F TsaC with different ratios of NAD$^+$ in the presence of 1,4-benzenedicarboxylate is not altered. (**F**) The bar chart shows the melting points of Y155F TsaC with NAD$^+$, 4-(hydroxymethy)benzoate, 4-formylbenzoate, and 1,4-benzenedicarboxylate. On the x-axis, 1 is no compound addition, 2 is addition of alcohol, 3 is addition of aldehyde, and 4 is addition of acid. Indicated ratios represent the concentration of NAD$^+$ added to Y155F TsaC. In all panels, the data were measured using n = 3 independent experiments. In panel (A) to (E), the data are presented as the mean values of these measurements. In panel (F), the data are presented as the mean value ± SD of these measurements.

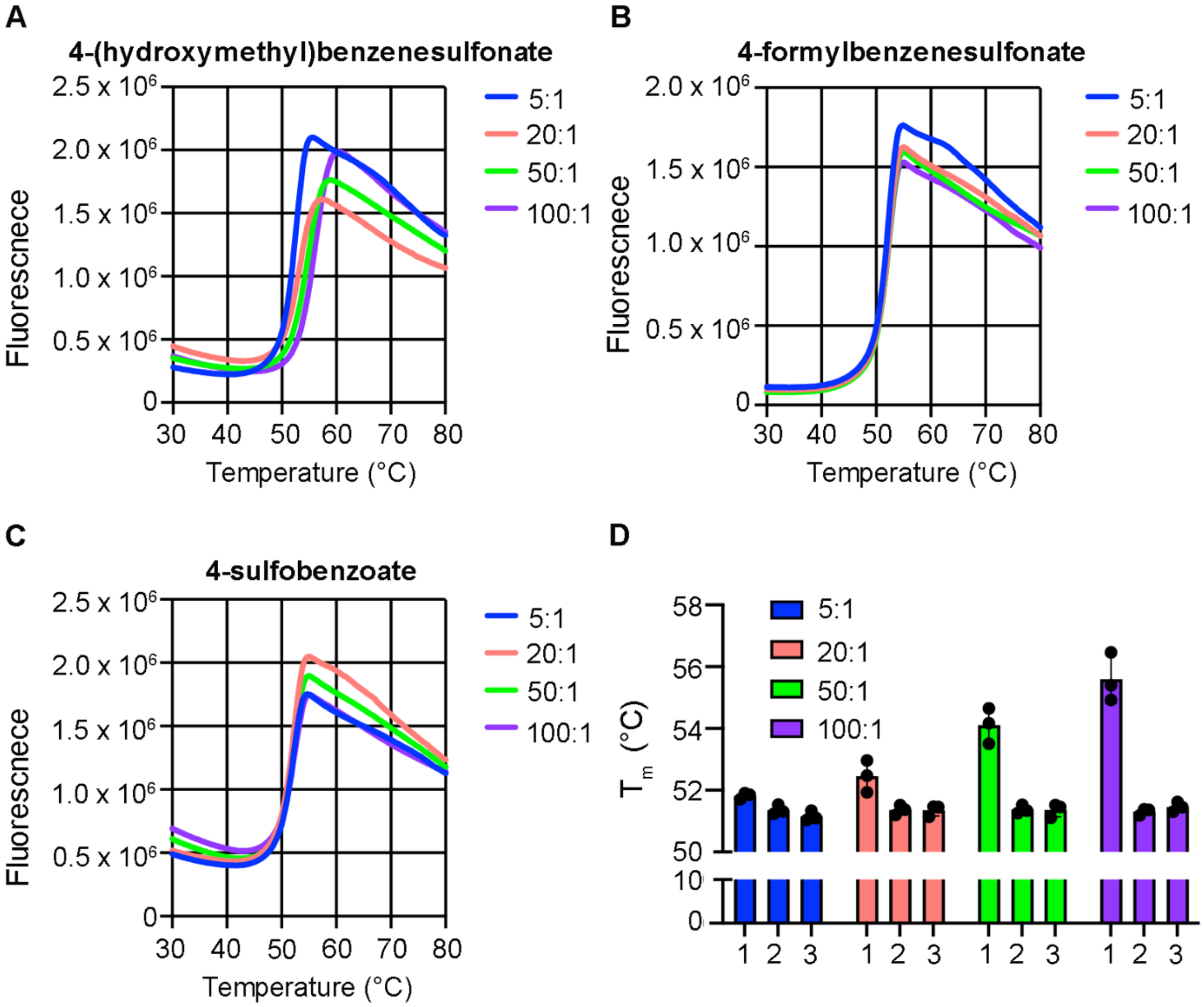

Figure S44. The thermal stability of Y155F TsaC with NAD^+, 4-(hydroxymethy)benzenesulfonate, aldehyde 4-formylbenzenesulfonate, and acid 4-sulfobenzoate were assayed and analyzed using differential scanning fluorimetry. (**A**) The thermal stability of different ratios of NAD^+ to Y155F TsaC with the addition of 4-(hydroxymethyl)benzenesulfonate was probed. In the presence of both 4-(hydroxymethyl)benzenesulfoante and NAD^+, the melting point increased, suggesting elevated thermal stability. (**B**) The thermal denaturation curve of different ratios of NAD^+ to Y155F TsaC in the presence of 4-formylbezoate suggests that enzyme stability is not affected. (**C**) Similarly, the thermal stability of Y155F TsaC with different ratios of NAD^+ in the presence of 1,4-benzenedicarboxylate is not altered. (**D**) The bar chart shows the melting point of Y155F TsaC with NAD^+, alcohol, aldehyde and acid compounds. On the x-axis, 1 is addition of alcohol, 2 is addition of aldehyde, and 3 is addition of acid. Indicated ratios represent the concentration of NAD^+ added to Y155F TsaC. In all panels, the data were measured using n = 3 independent experiments. In panel (A) to (C), the data are presented as the mean values of these measurements. In panel (D), the data are presented as the mean value ± SD of these measurements.

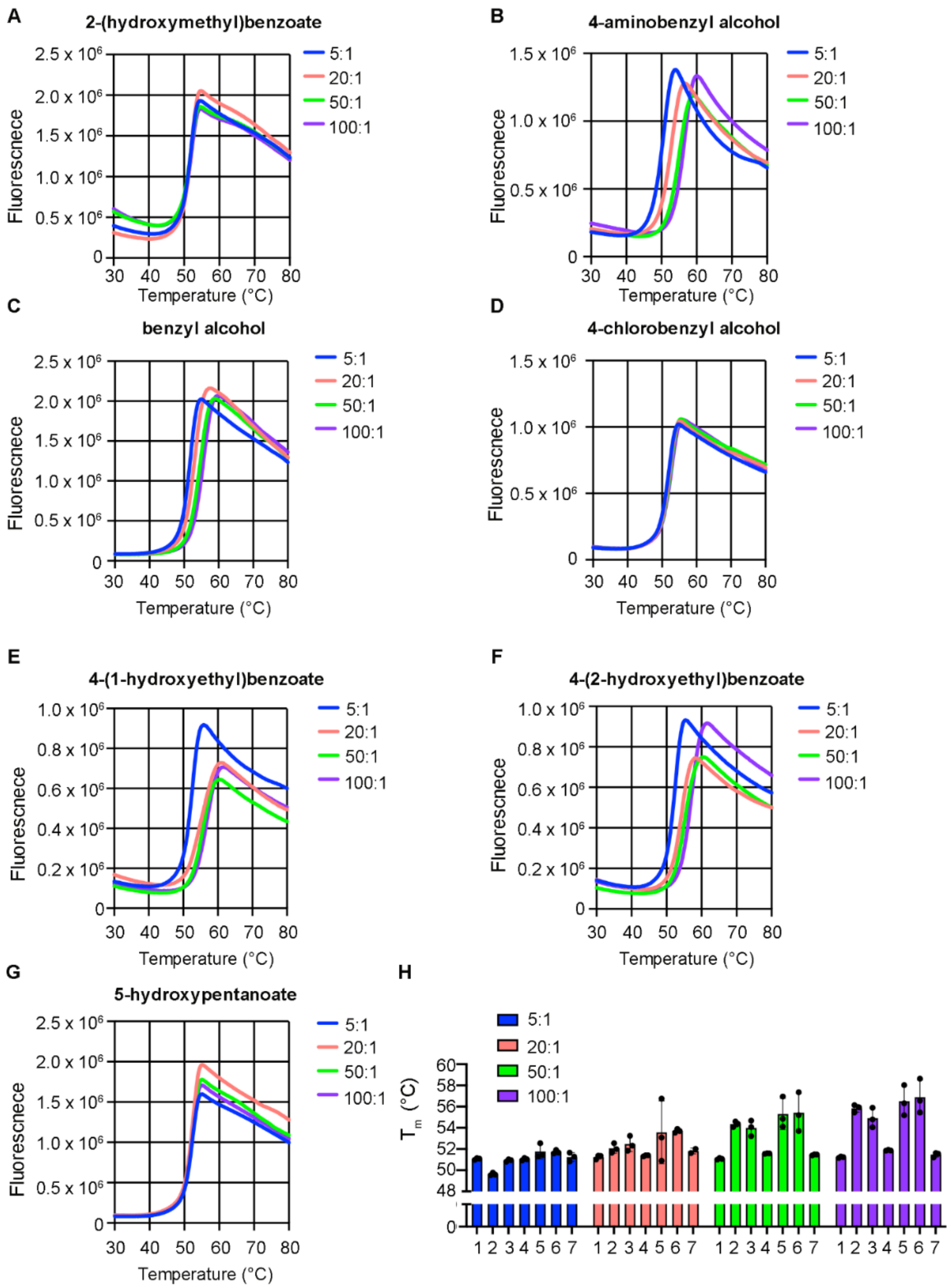

Figure S45. The thermal stability of Y155F TsaC with different substrates was probed and analyzed using differential scanning fluorimetry. Thermal denaturation curves shown here are organized by the substrate type, with panels (**A**) 2-(hydroxymethyl)benzoate, (**B**) 4-aminobenzyl alcohol, (**C**) benzyl alcohol, (**D**) 4-chlorobenzyl alcohol, (**E**) 4-(2-hydroxyethyl)benzoate, (**F**) 4-(1-hydroxyethyl)benzoate and (**G**) 5-hydroxypentanone. The thermal stability of Y155F TsaC with different ratios of NAD^+ increases in the presence of 4-aminobenzyl alcohol, benzyl alcohol, 4-(2-hydroxyethyl)benzoate, and 4-(1-hydroxyethyl)benzoate, which is consistent with enzymatic assay results. (**H**) The bar graph of the melting temperatures is shown. In all panels, the data were measured using n = 3 independent experiments. In panel (A) to (G), the data are presented as the mean values of these measurements. In panel (H), the data are presented as the mean value ± SD of these measurements. Indicated ratios represent the concentration of NAD^+ added to Y155F TsaC. On the x-axis, 1 is addition of 2-(hydroxymethyl)benzoate, 2 is addition of

4-aminobenzyl alcohol, 3 is addition of benzyl alcohol, 4 is addition of 4-chlorobenzyl alcohol, 5 is addition of 4-(1-hydroxyethyl)benzoate, 6 is addition of 4-(2-hydroxyethyl)benzoate, 7 is addition of 5-hydroxypentanoate. In all panels, the data were measured using n = 3 independent experiments

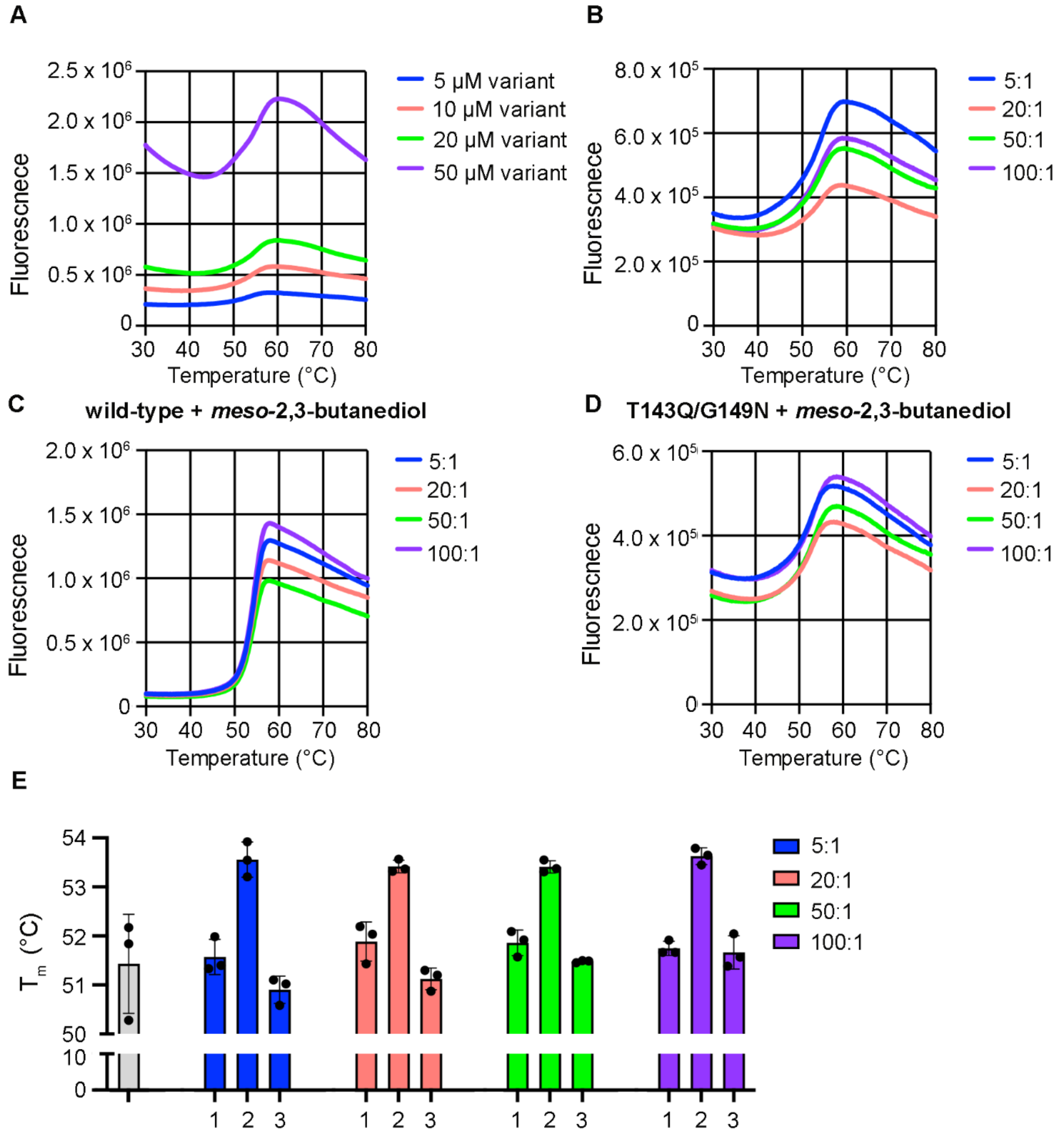

Figure S46. The thermal stability of T143Q/G149N TsaC with NAD$^+$ and *meso*-2,3-butadiol was probed and analyzed using differential scanning fluorimetry. (**A**) The thermal stability of different concentrations of T143Q/G149N TsaC was measured. An enzyme concentration of 10 μM was chosen as the optimal concentration for the following assays. (**B**) The thermal stability of different ratios of NAD$^+$ to T143Q/G149N TsaC is similar. (**C**) The thermal stability of wild-type TsaC with different concentrations of NAD$^+$ in the presence of *meso*-2,3-butadiol does not change. (**D**) Similarly, addition of *meso*-2,3-butadiol to the T143Q/G149N variant of TsaC with different ratios of NAD$^+$ does not change the thermal stability. (**E**) A bar chart to compare the thermal stability under the conditions noted in panels A-D. On the x-axis, 1 is T143Q/G149N TsaC with NAD$^+$, 2 is wild-type TsaC with NAD$^+$ and *meso*-2,3-butadiol, and 3 is T143Q/G149N TsaC with NAD$^+$ and *meso*-2,3-butadiol. Indicated ratios represent the concentration of NAD$^+$ added to Y155F TsaC. Gray is the T143Q/G149N TsaC variant. In all panels, the data were measured using n = 3 independent experiments. In panel (A) to (D), the data are presented as the mean values of these measurements. In panel (E), the data are presented as the mean value ± SD of these measurements.

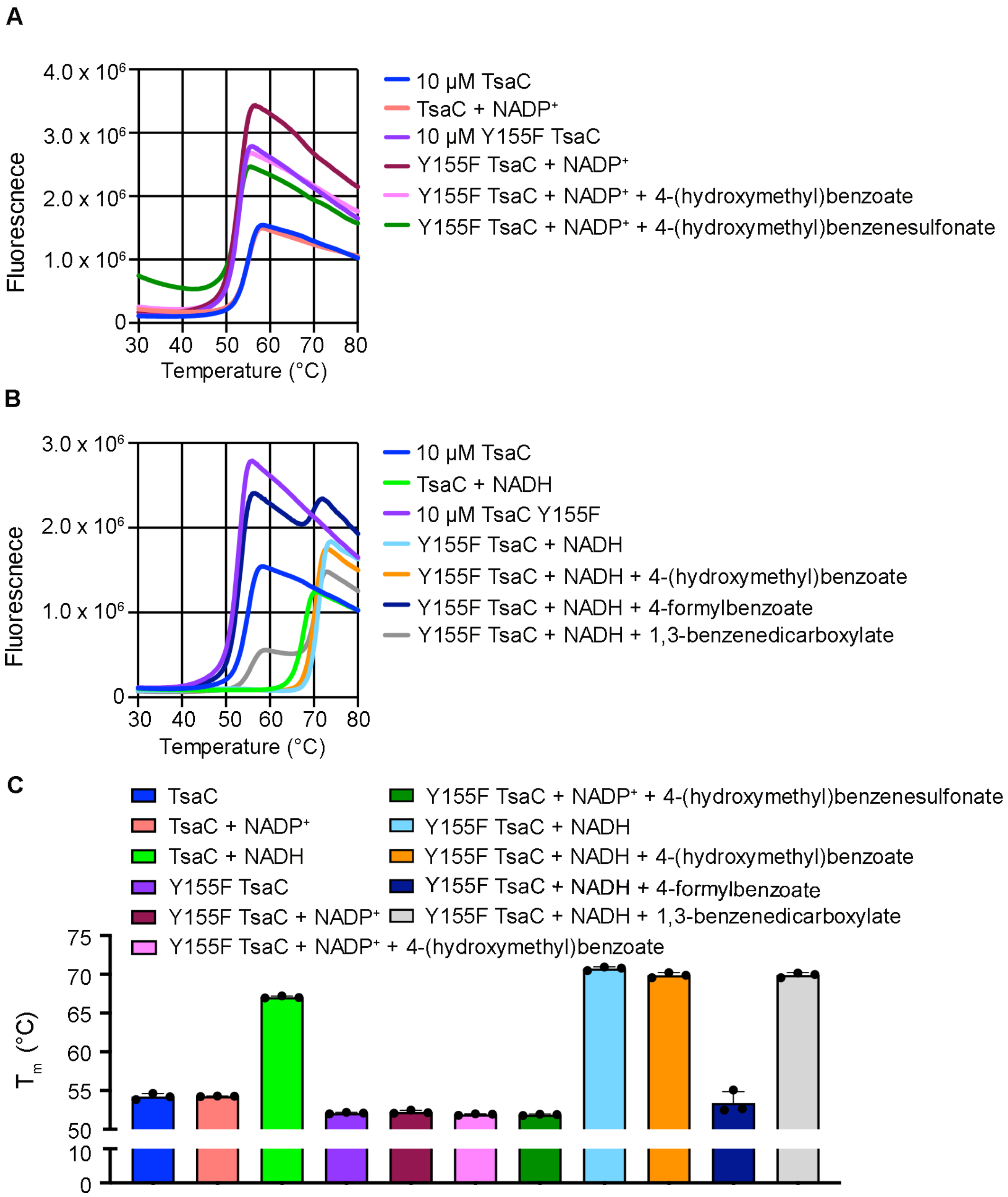

Figure S47. Wild-type TsaC and Y155F TsaC with $NADP^+$ or NADH, 4-(hydroxymethyl)benzoate, 4-formylbenzoate, and 1,4-benzenedicarboxylate were assayed and analyzed using differential scanning fluorimetry. For all experiments, 10 µM of enzyme and 100-fold excess of either $NADP^+$ or NADH was used. (**A**) Melting curves of wild-type TsaC when $NADP^+$ or $NADP^+$ and substrate is added show that the thermal stability of TsaC is not altered by the addition of $NADP^+$ or $NADP^+$ and substrate, indicating that consistent with the sequence and structure, $NADP^+$ likely does not bind to TsaC. (**B**) Melting curves of wild-type TsaC or Y155F TsaC when NADH is added, with or without substrate or product. A large shift when NADH is added is indicative of TsaC having higher thermal stability. The melting curve for wild-type TsaC is replotted from panel A for comparison. For 4-(hydroxymethyl)benzoate and 1,4-benzenedicarboxylate, a similar shift in thermal stability is observed as described in panel B, but this shift is not observed when 4-formylbenzoate is added. (**C**) A bar chart representing the T_m data calculated from the curves in panels A and B. Large increases in T_m occur when NADH is present in the mixture, with an exception for when 4-formylbenzoate is also present.

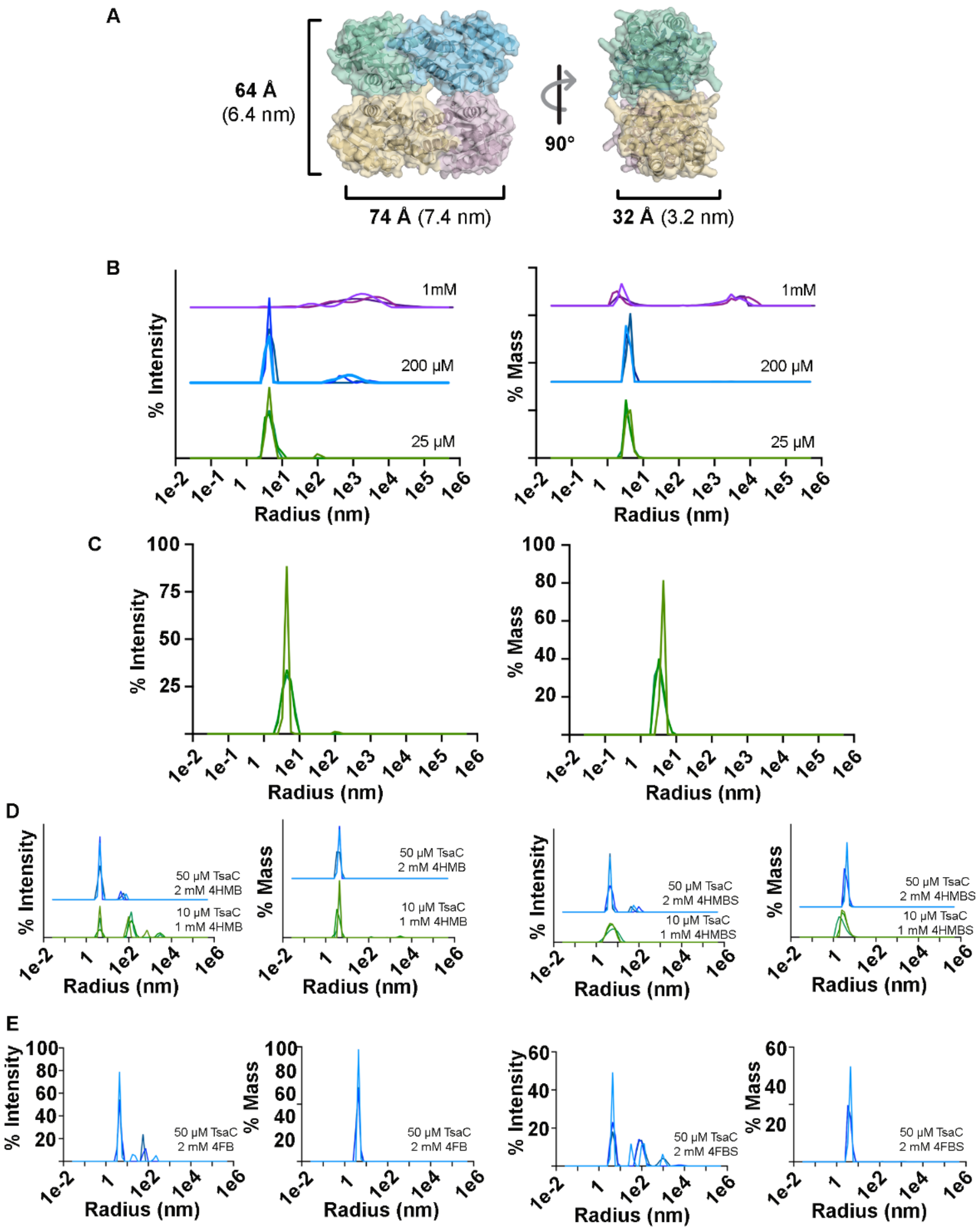

Figure S48. Dynamic light scattering experiments indicate that the radius of hydration of the dominant species of Y155F TsaC is consistent with the tetramer. Data for two or three data segments, spaced out every 1 min, for each sample are displayed. (**A**) Measured dimensions of the crystal structure contextualize the hydrodynamic radius of dynamic light scattering results. (**B**) Y155F TsaC in buffered solution at low (25 µM), high (200 µM) and very high (1 mM) concentration illustrates a reversible formation of a higher-order oligomeric state at high protein concentration. The broadening of the large-radius species indicates a likely multiple higher-order species. These assays were all performed with a 2 mM protein stock solution, indicating the reversibility of the formation of the larger species. (**C**) Including NAD+ in the solution does not appear to affect the oligomeric state of Y155F TsaC. (**D**) In the presence of NAD+ and substrate (4-hydroxymethylbenzoate; 4HMB left or 4-hydroxymethylbenzenesulfonate; 4HMBS right), there is a small amount of the higher order species, but the major species is still consistent with the tetramer. (**E**) In the presence of NADH and product (4-formylbenzoate; 4FB left or 4-formylbenzenesulfonate; 4FBS right),

there is a small amount of the higher order species present, but the major species by %mass is still consistent with the tetramer.

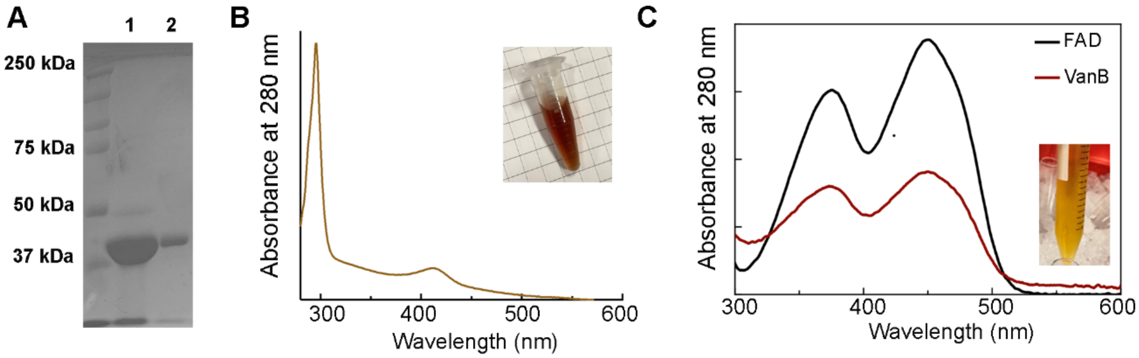

Figure S49. TsaM and VanB were recombinantly expressed and purified for use in enzyme cascade experiments. (**A**) The purities of recombinantly expressed and purified TsaM (lane 1) and VanB (lane 2) were assessed using SDS-PAGE. The expected molecular weights of TsaM and VanB are 38 kDa and 37 kDa, respectively. (**B**) As evidenced by the characteristic absorbance peak at approximately 420 nm, TsaM purifies with the Rieske [2Fe-2S] cluster intact. (**C**) Similarly, measurement of the UV-Vis absorbance maximum at 450 nm reveals that the FAD of VanB is intact following purification.

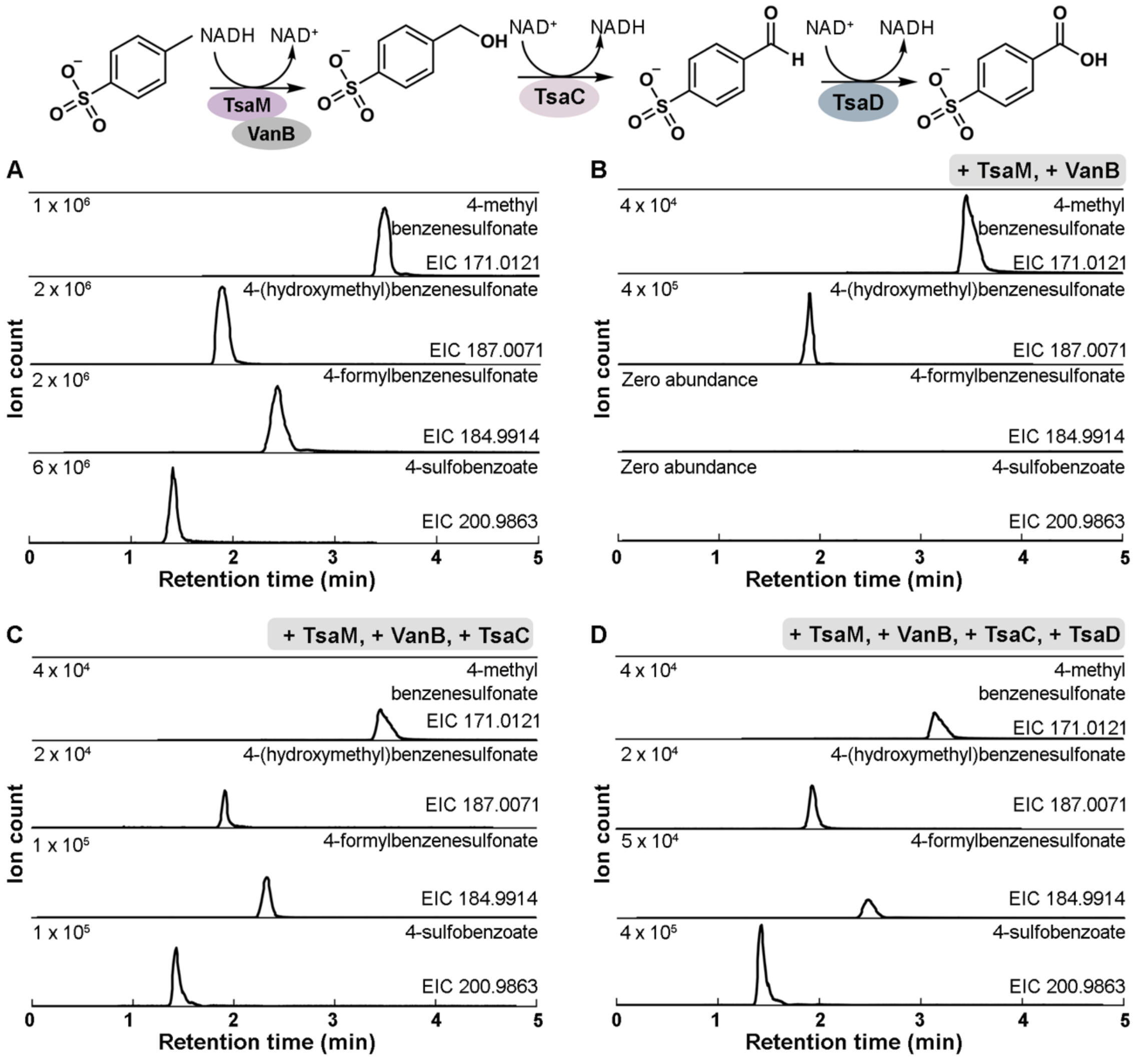

Figure S50. The combination of TsaM, VanB, TsaC, and TsaD results in the conversion of 4-methylbenzenesulfonate into 4-sulfobenzoate. (**A**) Commercially purchased standards were used to evaluate the results of the enzyme cascade experiment. (**B**) The combination of TsaM with VanB results in formation of 4-(hydroxymethyl)benzenesulfonate. (**C**) The combination of TsaM, VanB, and TsaC results in formation of 4-(hydroxymethyl)benzenesulfonate, 4-formylbenzenesulfonate, and 4-sulfobenzoate. (**D**) The combination of TsaM, VanB, TsaC, and TsaD results in formation of the same products shown in panel C, but the amount of 4-sulfobenzoate is higher when TsaD is included in the cascade.

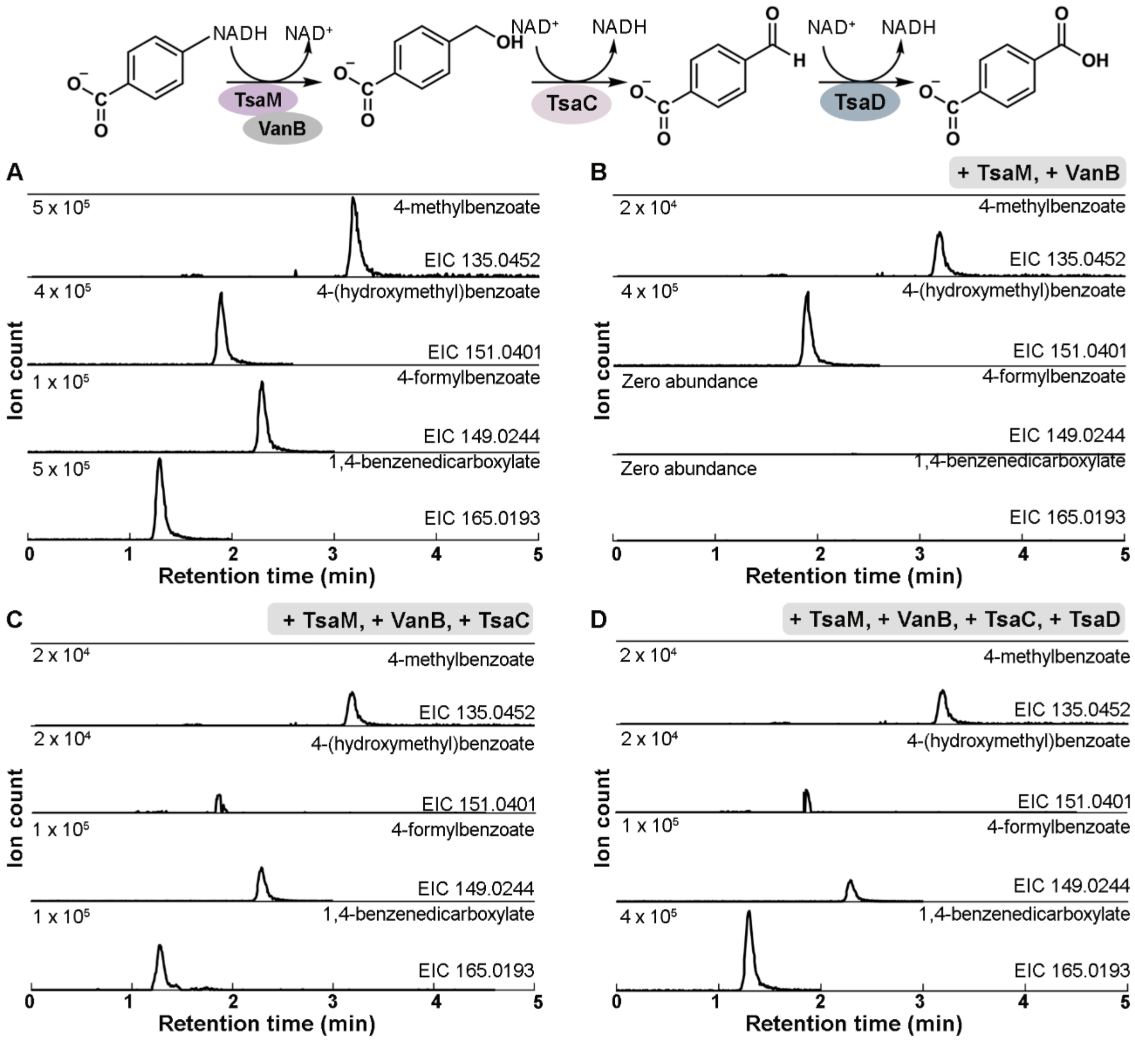

Figure S51. The combination of TsaM, VanB, TsaC, and TsaD results in the conversion of 4-methylbenzoate into 1,4-benzenedicarboxylate. (**A**) Commercially purchased standards were used to evaluate the results of the enzyme cascade experiment. (**B**) The combination of TsaM, VanB, and NAD^+ results in formation of 4-(hydroxymethyl)benzoate. (**C**) The combination of TsaM, VanB, and TsaC results in formation of 4-(hydroxymethyl)benzoate, 4-formylbenzoate, and 1,4-benzenedicarboxylate. (**D**) The combination of TsaM, VanB, TsaC, and TsaD results in formation of the same products shown in panel C, but the amount of 4-formylbenzoate is higher when TsaD is included in the cascade.

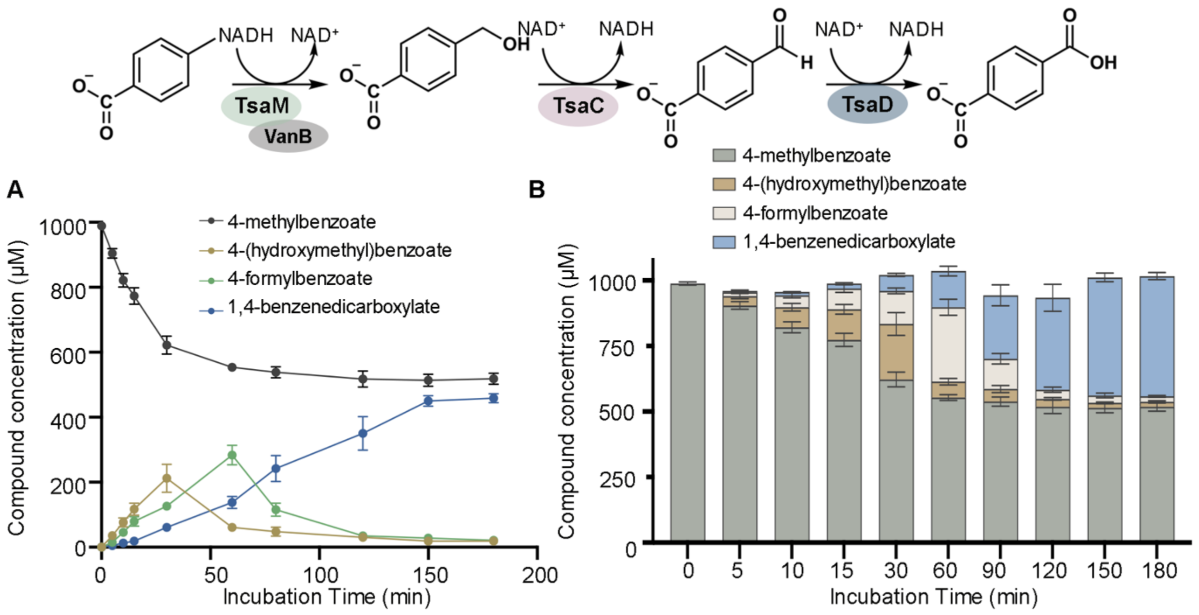

Figure S52. The combination of TsaM, VanB, TsaC, and TsaD with 4-methylbenzoate results in the iterative formation of 4-(hydroxymethyl)benzoate, 4-formylbenzoate, and 1,4-benzenedicarboxylate. (**A**) In this enzyme cascade experiment, 4-methylbenzoate is consumed over time and iteratively converted in alcohol, aldehyde, and carboxylic acid products. (**B**) The data from panel A is plotted as a bar graph to reveal the mass balance of the different products formed over time. The data in this panel were measured using n = 3 independent experiments and are represented as the mean value of those measurements.

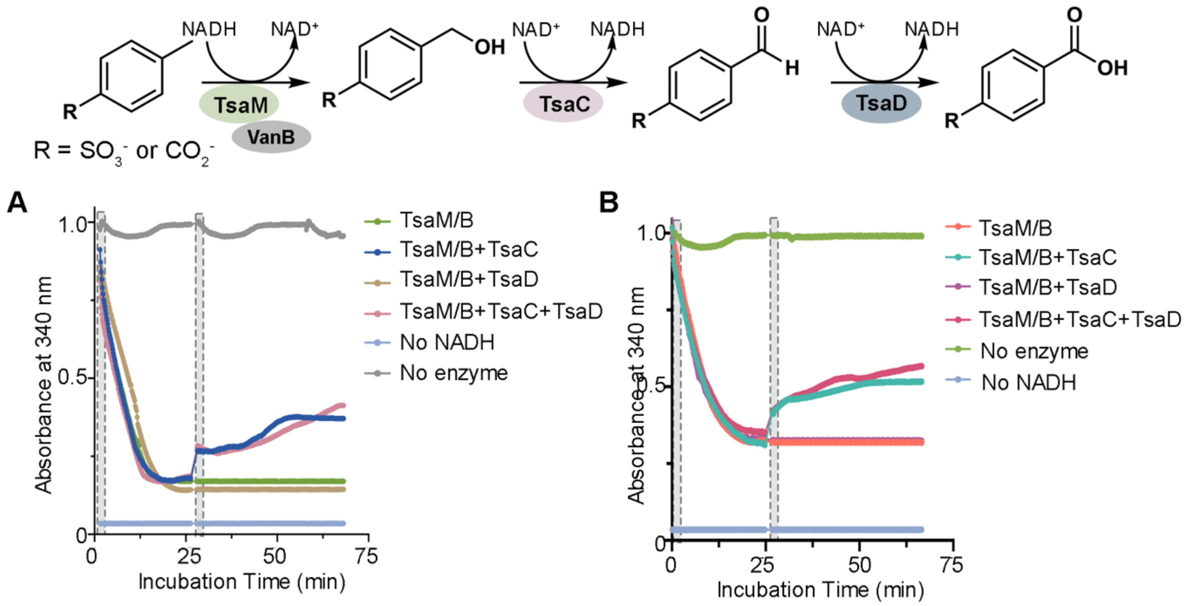

Figure S53. TsaM and VanB consume NADH and TsaC and TsaD regenerate NADH. (**A**) NADH is consumed in assays that contain TsaM, VanB, NADH, and 4-methylbenzenesulfonate. Addition of TsaC and TsaD to this NADH-consuming reaction allows for NADH production; TsaC and TsaD were injected at the time point marked by a gray rectangle. (**B**) NADH is consumed in assays that contain TsaM, VanB, NADH, and 4-methylbenzoate. Addition of TsaC and TsaD to this NADH-consuming reaction allows for NADH production; TsaC and TsaD were injected at the time point marked by a gray rectangle. The data in this figure were measured using n = 3 independent experiments and are plotted as the average of those data points.

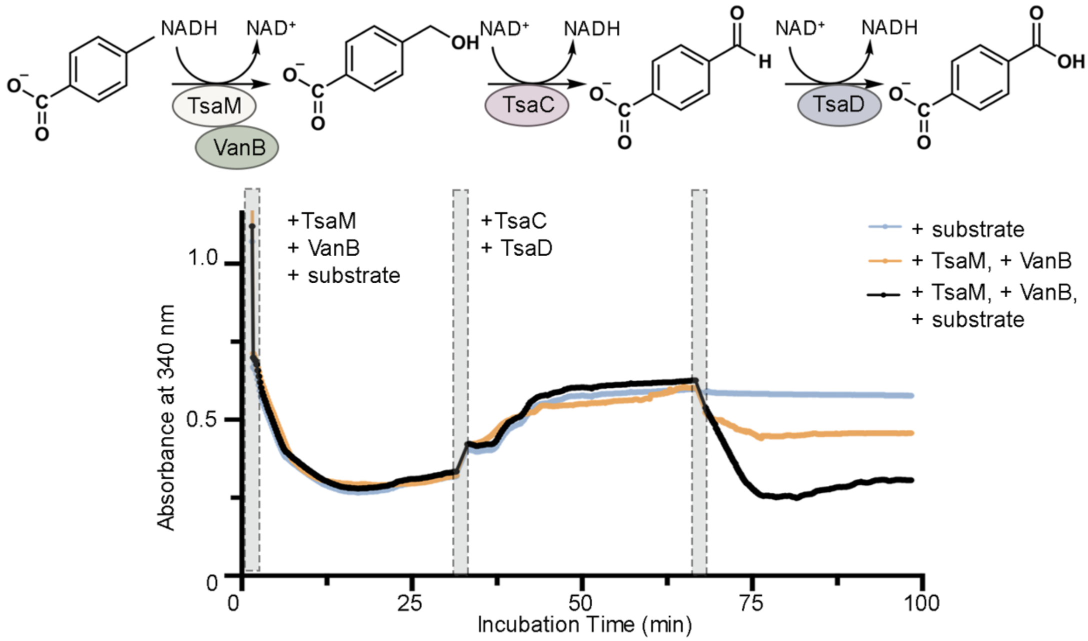

Figure S54. NADH is consumed in assays that contain TsaM, VanB, NADH, and 4-methylbenzoate. Addition of TsaC and TsaD to this NADH-consuming reaction allows for NADH production; TsaC and TsaD were injected at the time point marked by a gray rectangle. Likewise, addition of 4-methylbenzoate, TsaM and VanB, or TsaM, VanB, and 4-methylbenzenesulfonate at the 60-minute mark promotes NADH consumption.

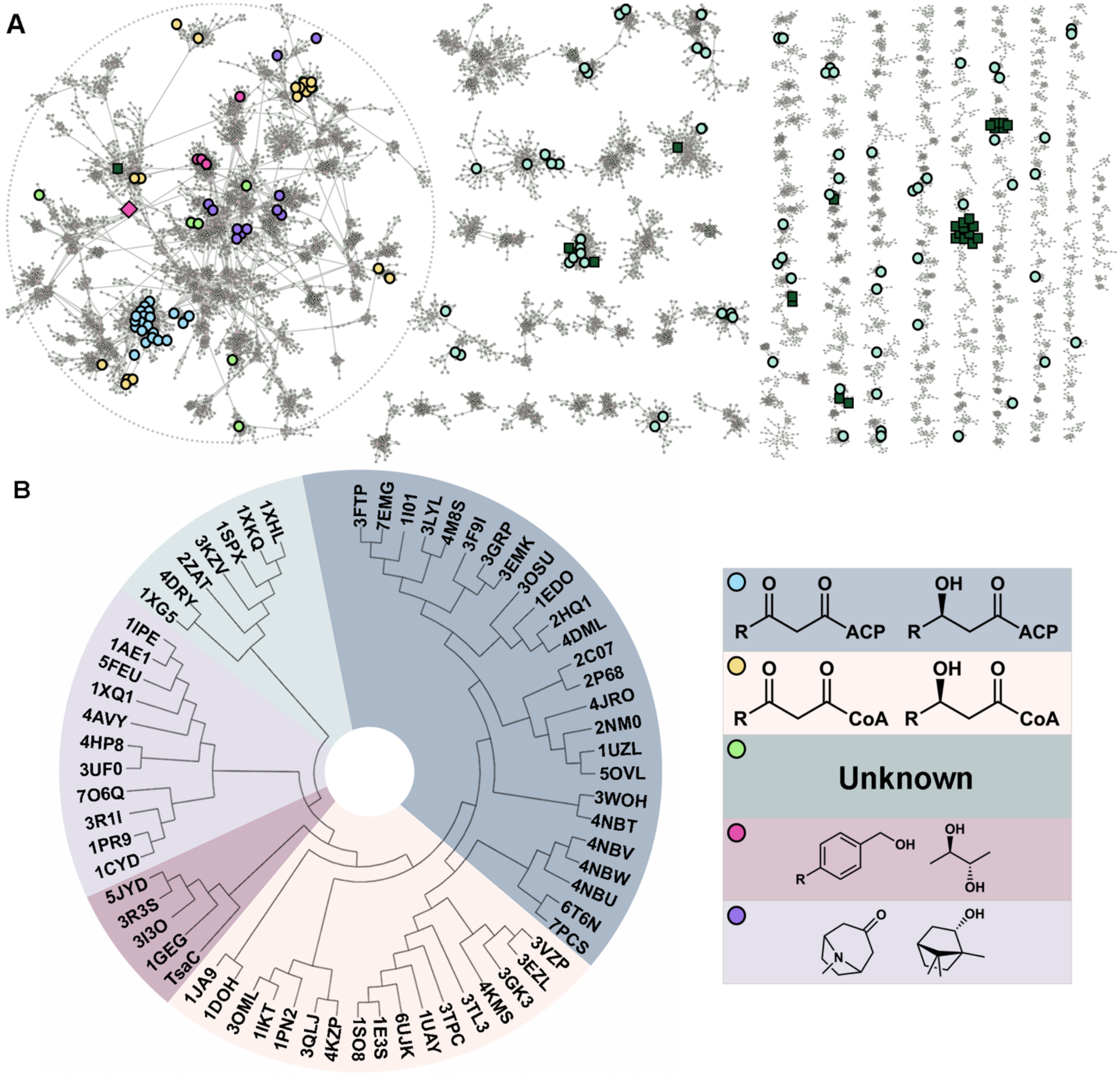

Figure S55. Bioinformatic analysis of the SDR enzyme family shows distribution of structurally characterized SDR enzymes and SDR enzymes that co-occur with Rieske oxygenases. (**A**) A UniRef50 sequence similarity network (SSN) of the SDR enzyme family separated at an alignment score threshold of 67 shows TsaC (pink diamond) remains in the largest node cluster in the SSN (5764 nodes; 390 nodes are found in the next largest cluster). Structurally characterized SDR enzymes are highlighted in larger circles; in the largest node circle they are colored by representative substrate and outside of it they are colored light green. SDRs that co-occur with Rieske oxygenases are shown as dark green squares. (**B**) A phylogenetic tree of the structurally characterized SDR enzymes in the main cluster, including TsaC, suggests an evolutionary relationship arranged by substrate type. When dividing the tree into clades based on substrate type, the largest two clades are represented by SDR enzymes that typically operate on 3*R*-hydroxyacyl-acyl carrier protein/3-oxoacyl-acyl carrier protein or 3*R*-hydroxyacyl-coenzyme A/3-oxoacyl-coenzyme A. The third largest clade generally accepts complex cyclic compounds as substrates, and the fourth largest clade does not have known substrates. The smallest clade, including TsaC, represents an outlier accepting various small molecules as substrates.

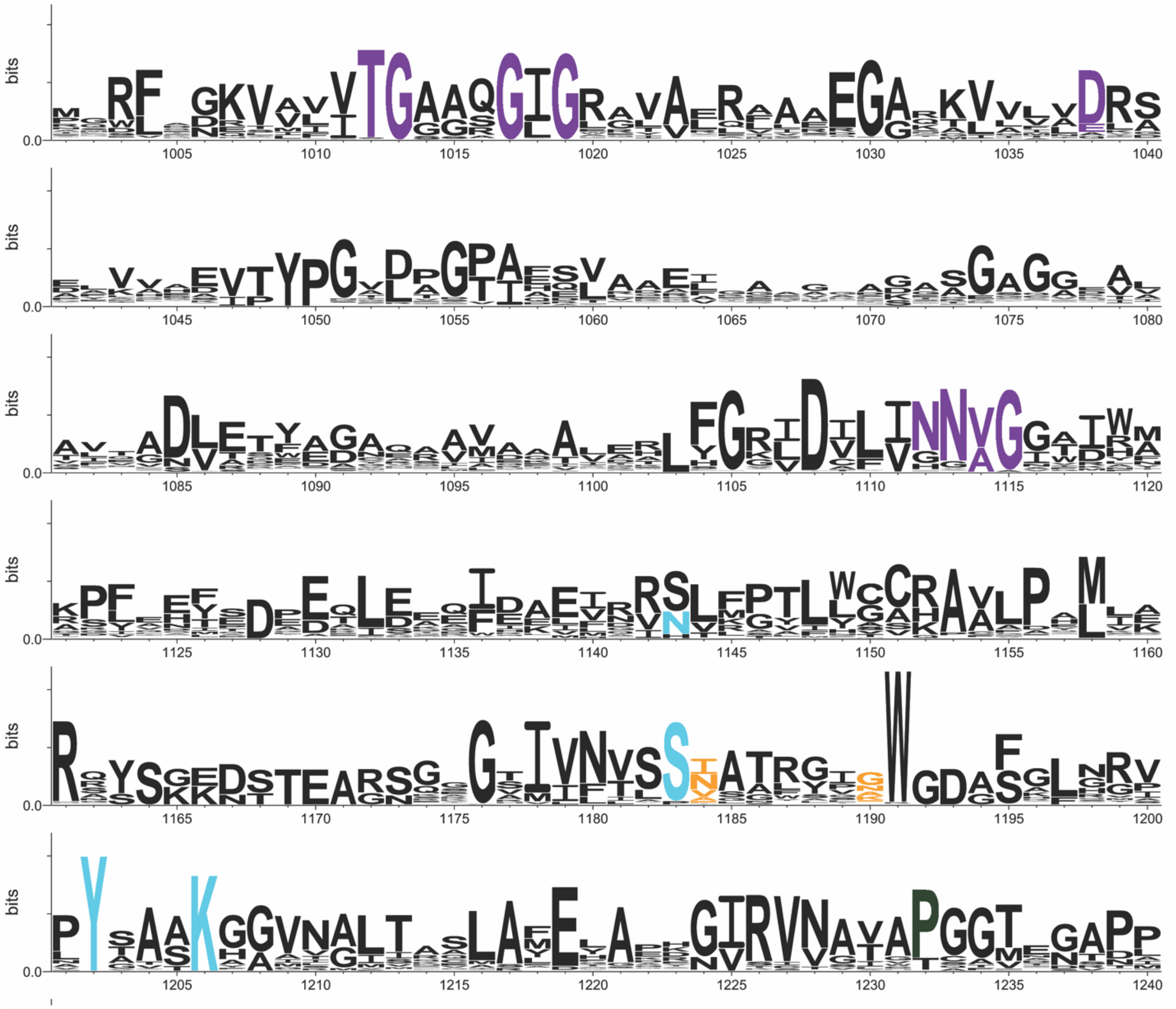

Figure S56. A logo-diagram comprised of the SDR enzymes that co-occur in gene clusters with Rieske oxygenases highlights conserved classical SDR enzyme sequence motifs. The TGX$_3$GXG motif and the so-called NNAG motif are predominant in the alignment, indicating that most of these enzymes are likely classical SDR enzymes (residues in purple). A predominant Asp or Glu residue that aligns with Asp36 in TsaC, the residue that has been implicated in dictating preference for NAD(H), indicates that the majority of these enzymes likely use NAD(H) (residue in purple). The catalytic tetrad (residues in light blue) is also predominant, with 100-percent conservation of the Tyr and Lys resides. There is notable variation in the Asn residue, as it is sometimes replaced with a Ser or His residue, which may play a similar role. In the β5-α6 loop, beginning with the gating residue that flanks the catalytic Ser residue and spanning through the space-opening Gly residues (residues in orange) likely play an important role in substrate binding for the co-occurring SDR enzymes.

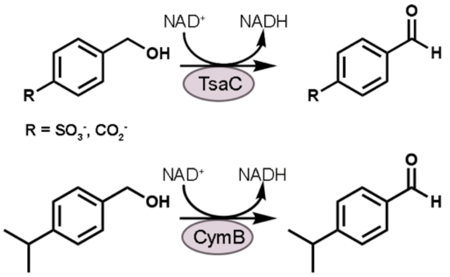


```
CymB            MRLKDKVAIVTGAATGIGNAIVRSYLAEGAKVVIADVKGAEAA--AAE--LGEDLALGVF 56
TsaC            MNLNKQVAIVTGGASGFGAAIARRLSQAGAAVLVADLNAEGAQRMATELNAAGGRALGMA 60
                *.*:.:******.*:*:* **.*     ** *::**::.  *   *:*   . . ***:

CymB            ADVSDPDSTKQMAKAALDRFGKIDVLVNNAGIFTGLNYVPMESISVADWDKLYSVNVKGP 116
TsaC            CDVSKEADYRAVVDAAIAQLGGLHIVVNNAGTTHR--NKPALAVTEDEFDRVYRVNLKSV 118
                .***.  . : :..**: ::* :.::*****        *  :::  ::*::* **:*.

CymB            WLCASAVSAAMREGGGGKIINIASVIAHIGAPFMLHYVSSKGAVAAMTRAMAREFATTKA 176
TsaC            YWSAQCALPHFAQQGHGVMVNVASTTGVRPGPGLTWYSGSKAAMINLTKGLALEFARS-- 176
                : .*...   : : * * ::*:**. .   .* :  * .**.*:  :*:.:* *** :

CymB            GISVNSISPGYTHSEN--ALANAQQHEQFEGVSASMRAIDRPQVPADIAGVALWLASDEA 234
TsaC            GVRINAVNPMIGETPMMADFMGMEDTPANRERFLSRIPLGRFTRPDDVASAVAFLASDDA 236
                *: :*::.*   .:     : . ::    .    *   :.*   * *:*... :****:*

CymB            SYVNGQNIVVDGGIYMSL   252
TsaC            SFLTGVCLDVDGGRNI--   252
                *::.*  : ****  :
```

Figure S57. The alcohol dehydrogenase CymB may employ a similar strategy in the "gating" residue fingerprint in substrate selectivity. (A) A diagram showing the transformations catalyzed by TsaC and CymB (19) illustrate the similarities of the substrates, and also the different identity of the handle: polar for TsaC and non-polar for CymB (B) A sequence alignment of TsaC and cymene dehydrogenase (CymB) reveals the presence of conserved sequence motifs for binding NAD(H) and facilitating catalysis. Both enzymes contain the TGX_3GXG, NNAG, and characteristic Asp33 residue for binding NAD(H) (purple) as well as the catalytic tetrad of residues, Asn, Ser, Tyr, and Lys (blue). Residues corresponding to the gating positions identified in TsaC are highlighted in orange, and suggest a common role for these residues in substrate recognition, with a small non-polar residue making space, similar to Gly146 and a Val residue replacing Thr, to presumably match the hydrophobic properties of cymene.

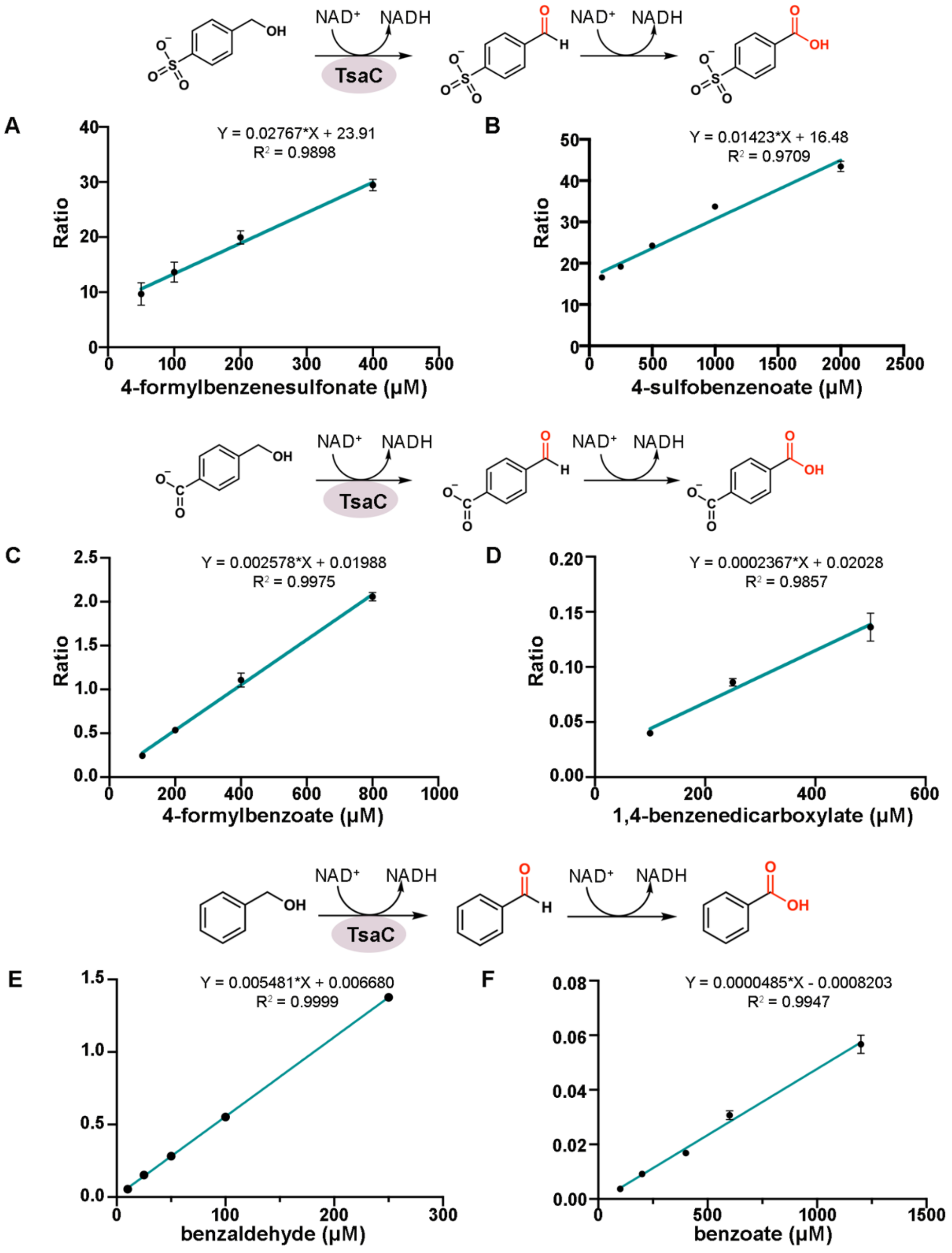
NAD+
NADH
TsaC
NAD+
NADH
OH
H
OH
A
Y = 0.02767*X + 23.91
R2 = 0.9898
Ratio
4-formylbenzenesulfonate (μM)
B
Y = 0.01423*X + 16.48
R2 = 0.9709
Ratio
4-sulfobenzenoate (μM)
C
Y = 0.002578*X + 0.01988
R2 = 0.9975
Ratio
4-formylbenzoate (μM)
D
Y = 0.0002367*X + 0.02028
R2 = 0.9857
Ratio
1,4-benzenedicarboxylate (μM)
E
Y = 0.005481*X + 0.006680
R2 = 0.9999
benzaldehyde (μM)
F
Y = 0.0000485*X - 0.0008203
R2 = 0.9947
benzoate (μM)

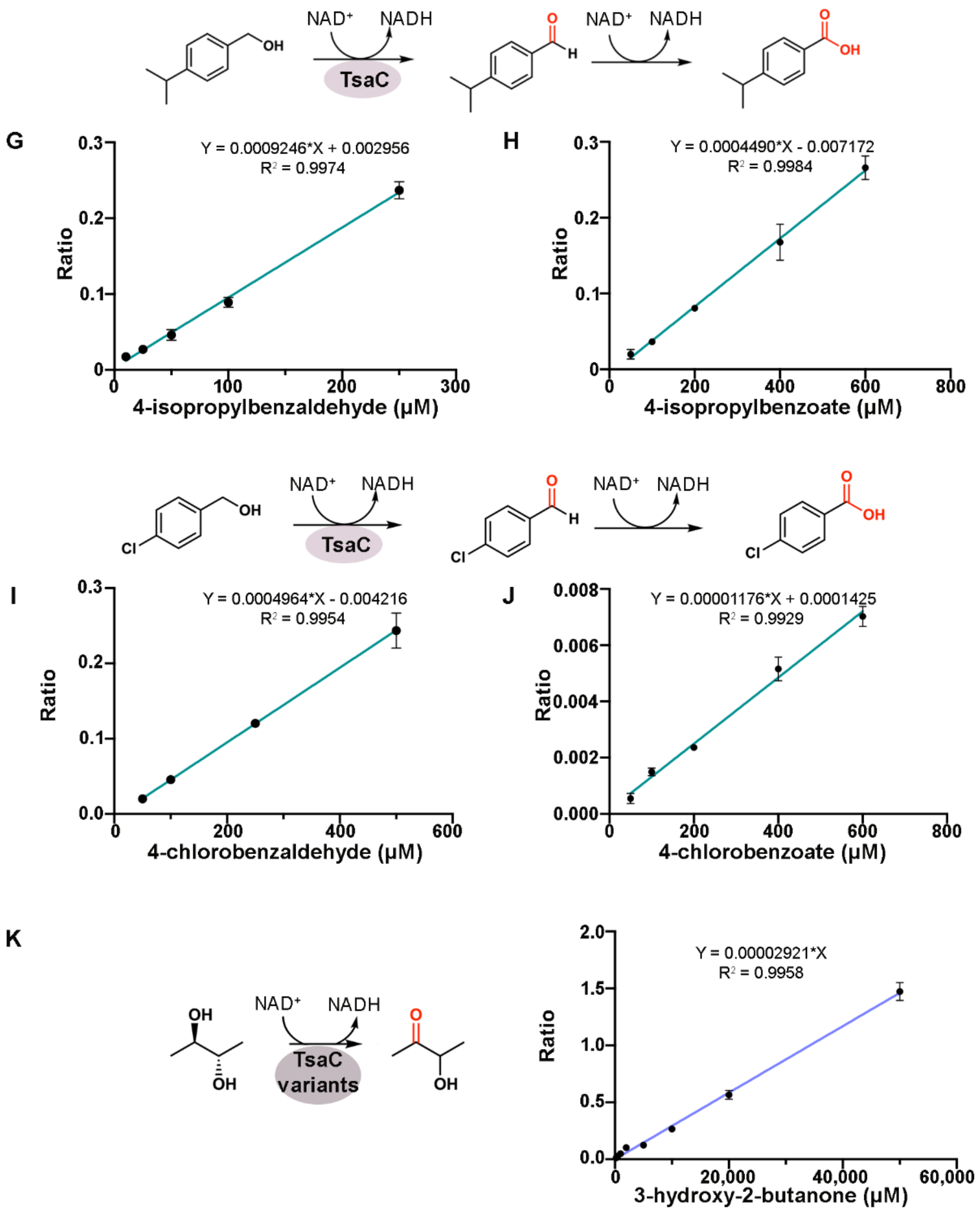

Figure S58. The constructed product standard curves were used to quantify the amount of product generated from LC-MS (all panels except K) and GC-MS (panel K) based enzymatic assays. In all panels, the x-axis corresponds to the amount of product (μM) and y-axis represents the ratio of product peak to internal standard peak. In order, the standard curves shown are: (A) 4-formylbenzenesulfonate, (B) 4-sulfobenzoate, (C) 4-formylbenzoate, (D) 1,4-benzenedicarboxylate, (E) benzaldehyde, (F) benzoate, (G) 4-isopropylbenzaldehyde, (H) 4-isopropylbenzoate, (I) 4-chlorobenzaldehyde, (J) 4-chlorobenzoate, (K) 3-hydroxy-2-butanone. In all panels, the data were measure using n = 3 experiments and are presented as the mean value ± SD of these measurements.

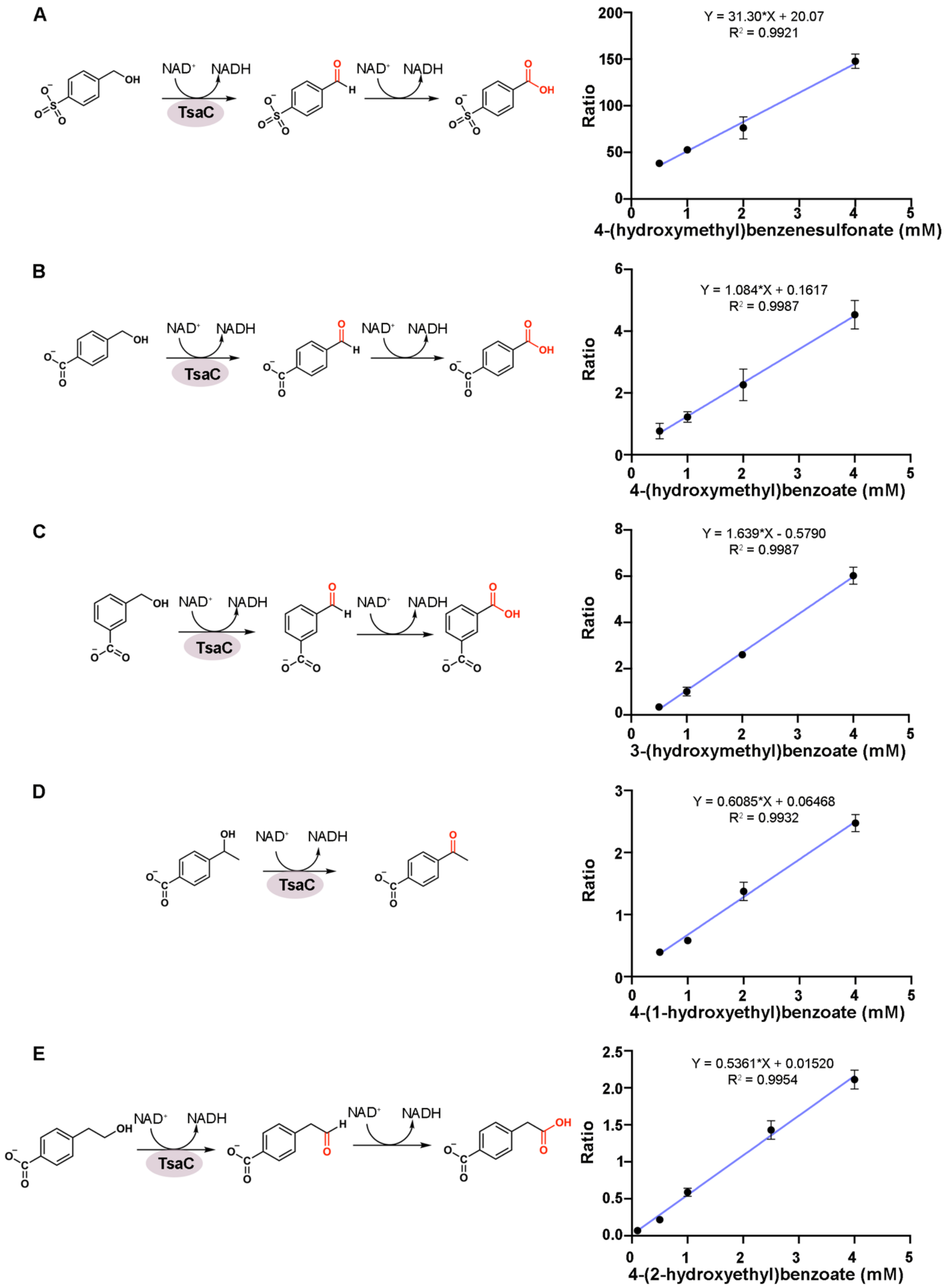
A
NAD+
NADH
TsaC
Y = 31.30*X + 20.07
R2 = 0.9921
Ratio
4-(hydroxymethyl)benzenesulfonate (mM)
B
NAD+
NADH
TsaC
Y = 1.084*X + 0.1617
R2 = 0.9987
Ratio
4-(hydroxymethyl)benzoate (mM)
C
NAD+
NADH
TsaC
Y = 1.639*X - 0.5790
R2 = 0.9987
Ratio
3-(hydroxymethyl)benzoate (mM)
D
NAD+
NADH
TsaC
Y = 0.6085*X + 0.06468
R2 = 0.9932
Ratio
4-(1-hydroxyethyl)benzoate (mM)
E
NAD+
NADH
TsaC
Y = 0.5361*X + 0.01520
R2 = 0.9954
Ratio
4-(2-hydroxyethyl)benzoate (mM)

F

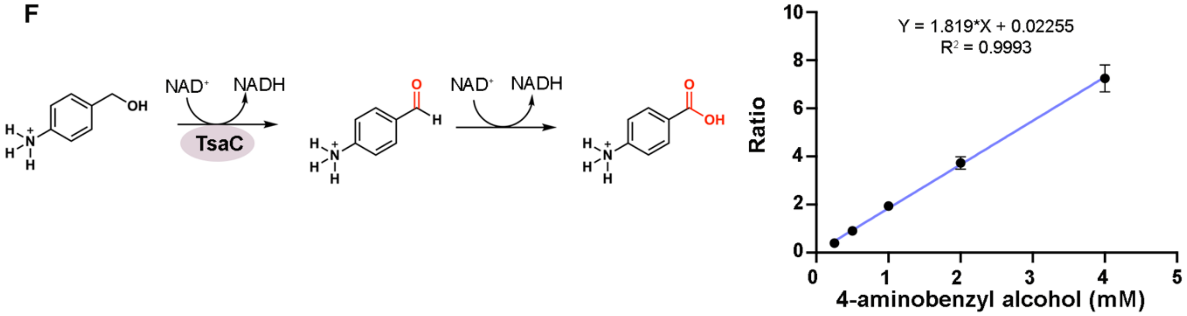

G

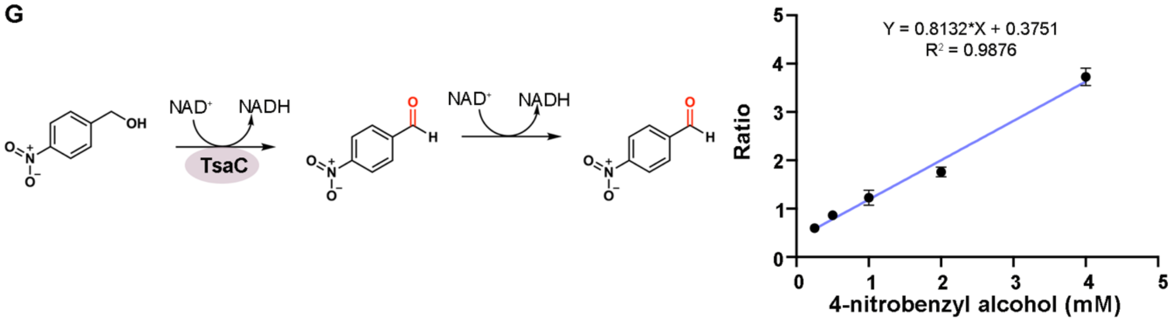

H

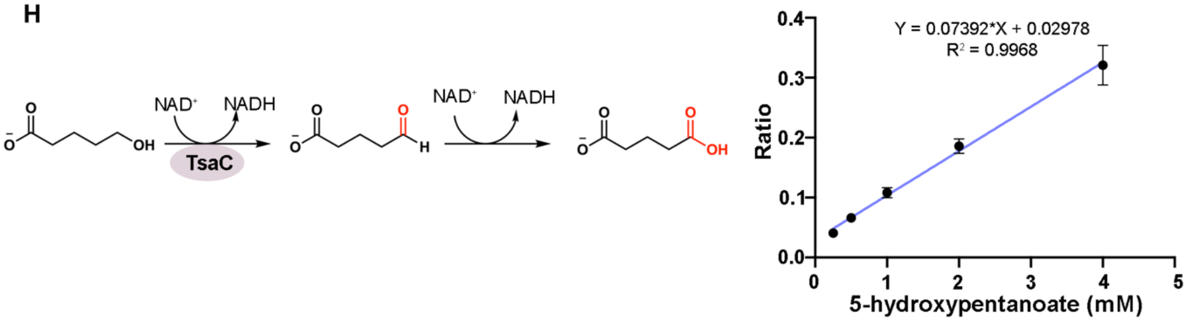

Figure S59. The constructed substrate standard curves were used to quantify the amount of substrate consumed from LC-MS based enzymatic assays. In all panels, the x-axis corresponds to the amount of substrate (mM) and y-axis represents the ratio of product peak to internal standard peak. In order, the standard curves shown are: (**A**) 4-(hydroxymethyl)benzenesulfonate, (**B**) 4-(hydroxymethyl)benzoate, (**C**) 3-(hydroxymethyl)benzoate, (**D**) 4-(2-hydroxyethyl)benzoate, (**E**) 4-(1-hydroxyethyl)benzoate, (**F**) 4-aminobenzyl alcohol, (**G**) 4-nitrobenzyl alcohol. In panels, the data were measure using n = 3 experiments and are presented as the mean value ± SD of these measurements.

References

1. Junker, F., Kiewitz, R., and Cook, A. M. (1997) Characterization of the p-toluenesulfonate operon tsaMBCD and tsaR in Comamonas testosteroni T-2 J Bacteriol **179**, 919-927 10.1128/jb.179.3.919-927.1997
2. Junker, F., Saller, E., Oppenberg, H. R. S., Kroneck, P. M. H., Leisinger, T., and Cook, A. M. (1996) Degradative pathways for p-toluenecarboxylate and p-toluenesulfonate and their multicomponent oxygenases in Comamonas testosteroni strains PSB-4 and T-2 Microbiol-Sgm **142**, 2419-2427 Doi 10.1099/00221287-142-9-2419
3. Locher, H. H., Leisinger, T., and Cook, A. M. (1991) 4-Toluene sulfonate methyl-monooxygenase from Comamonas testosteroni T-2: purification and some properties of the oxygenase component J Bacteriol **173**, 3741-3748 10.1128/jb.173.12.3741-3748.1991
4. Otagiri, M., Kurisu, G., Ui, S., Takusagawa, Y., Ohkuma, M., Kudo, T. *et al.* (2001) Crystal structure of meso-2,3-butanediol dehydrogenase in a complex with NAD+ and inhibitor mercaptoethanol at 1.7 A resolution for understanding of chiral substrate recognition mechanisms J Biochem **129**, 205-208 10.1093/oxfordjournals.jbchem.a002845
5. Filling, C., Nordling, E., Benach, J., Berndt, K. D., Ladenstein, R., Jornvall, H. *et al.* (2001) Structural role of conserved Asn179 in the short-chain dehydrogenase/reductase scaffold Biochem Biophys Res Commun **289**, 712-717 10.1006/bbrc.2001.6032
6. Krissinel, E., and Henrick, K. (2007) Inference of macromolecular assemblies from crystalline state J Mol Biol **372**, 774-797 10.1016/j.jmb.2007.05.022
7. Busing, I., Hoffken, H. W., Breuer, M., Wohlbrand, L., Hauer, B., and Rabus, R. (2015) Molecular Genetic and Crystal Structural Analysis of 1-(4-Hydroxyphenyl)-Ethanol Dehydrogenase from 'Aromatoleum aromaticum' EbN1 J Mol Microbiol Biotechnol **25**, 327-339 10.1159/000439113
8. Zaccai, N. R., Carter, L. G., Berrow, N. S., Sainsbury, S., Nettleship, J. E., Walter, T. S. *et al.* (2008) Crystal structure of a 3-oxoacyl-(acylcarrier protein) reductase (BA3989) from Bacillus anthracis at 2.4-A resolution Proteins **70**, 562-567 10.1002/prot.21624
9. Li, T. B., Zhao, F. J., Liu, Z., Jin, Y., Liu, Y., Pei, X. Q. *et al.* (2019) Structure-guided engineering of ChKRED20 from Chryseobacterium sp. CA49 for asymmetric reduction of aryl ketoesters Enzyme Microb Technol **125**, 29-36 10.1016/j.enzmictec.2019.03.001
10. Hou, J., Zheng, H., Chruszcz, M., Zimmerman, M. D., Shumilin, I. A., Osinski, T. *et al.* (2016) Dissecting the Structural Elements for the Activation of beta-Ketoacyl-(Acyl Carrier Protein) Reductase from Vibrio cholerae J Bacteriol **198**, 463-476 10.1128/JB.00360-15
11. Filling, C., Berndt, K. D., Benach, J., Knapp, S., Prozorovski, T., Nordling, E. *et al.* (2002) Critical residues for structure and catalysis in short-chain dehydrogenases/reductases J Biol Chem **277**, 25677-25684 10.1074/jbc.M202160200
12. Holm, L., Laiho, A., Toronen, P., and Salgado, M. (2023) DALI shines a light on remote homologs: One hundred discoveries Protein Sci **32**, e4519 10.1002/pro.4519
13. Sievers, F., Wilm, A., Dineen, D., Gibson, T. J., Karplus, K., Li, W. *et al.* (2011) Fast, scalable generation of high-quality protein multiple sequence alignments using Clustal Omega Mol Syst Biol **7**, 539 10.1038/msb.2011.75
14. Shortall, K., Djeghader, A., Magner, E., andSoulimane, T. (2021) Insights into Aldehyde Dehydrogenase Enzymes: A Structural Perspective Frontiers in Molecular Biosciences **8**, ARTN 65955010.3389/fmolb.2021.659550
15. Son, H. F., Park, S., Yoo, T. H., Jung, G. Y., and Kim, K. J. (2017) Structural insights into the production of 3-hydroxypropionic acid by aldehyde dehydrogenase from Azospirillum brasilense Sci Rep **7**, 46005 10.1038/srep46005

16. Otagiri, M., Ui, S., Takusagawa, Y., Ohtsuki, T., Kurisu, G., and Kusunoki, M. (2010) Structural basis for chiral substrate recognition by two 2,3-butanediol dehydrogenases FEBS Lett **584**, 219-223 10.1016/j.febslet.2009.11.068
17. Dhindwal, S., Patil, D. N., Mohammadi, M., Sylvestre, M., Tomar, S., and Kumar, P. (2011) Biochemical studies and ligand-bound structures of biphenyl dehydrogenase from Pandoraea pnomenusa strain B-356 reveal a basis for broad specificity of the enzyme J Biol Chem **286**, 37011-37022 10.1074/jbc.M111.291013
18. Park, A. K., Kim, H., Kim, I. S., Roh, S. J., Shin, S. C., Lee, J. H. *et al.* (2017) Crystal structure of cis-dihydrodiol naphthalene dehydrogenase (NahB) from Pseudomonas sp. MC1: Insights into the early binding process of the substrate Biochem Biophys Res Commun **491**, 403-408 10.1016/j.bbrc.2017.07.089
19. Eaton, R. W. (1997) p-Cymene catabolic pathway in Pseudomonas putida F1: cloning and characterization of DNA encoding conversion of p-cymene to p-cumate J Bacteriol **179**, 3171-3180 10.1128/jb.179.10.3171-3180.1997
